# Global burden, projections, and causal factors of maternal sepsis and other maternal infections: A comprehensive epidemiological and mendelian randomization study

**DOI:** 10.1371/journal.pntd.0014374

**Published:** 2026-05-27

**Authors:** Anqi Jiang, Siying Duan, Shuyun Wu, Wenkui Yu, Xiaohui Liang

**Affiliations:** 1 Department of Critical Care Medicine, Nanjing Drum Tower Hospital, Affiliated Hospital of Medical School, Nanjing University, Nanjing, Jiangsu, China; 2 Emergency Department, The Eighth Affiliated Hospital, Sun Yat-sen University, Shenzhen, Guangdong, China; UFSJ: Universidade Federal de Sao Joao del-Rei, BRAZIL

## Abstract

**Background:**

Maternal sepsis and other maternal infections (MSMI) remain major contributors to global maternal morbidity and mortality. However, the integration of epidemiological trends with causal inference evidence remains limited.

**Methods:**

Using data from the Global Burden of Disease (GBD) 2021 study, we assessed temporal trends in MSMI burden from 1990 to 2021 and projected future patterns using ARIMA and Bayesian age–period–cohort (BAPC) models. In parallel, we conducted a two-sample multivariable Mendelian randomization (MVMR) analysis to evaluate the causal effects of inflammatory biomarkers and related factors on MSMI risk.

**Results:**

Although age-standardized rates declined globally, absolute case numbers increased in low-SDI regions, largely driven by population growth. Forecasting results differed between ARIMA and BAPC models, reflecting distinct underlying assumptions regarding temporal dynamics. MVMR analysis identified inflammatory biomarkers, including CRP, IL-13, IL-10, RANTES, and NT-proBNP, as key causal factors associated with MSMI.

**Conclusions:**

This study provides the first integrated framework combining global disease burden analysis with multivariable MR. By linking population-level trends with causal inference, our findings offer dual evidence to support targeted prevention strategies and advance precision public health interventions for MSMI.

## Author summary

This study found that while global rates have improved overall, the total number of cases has actually grown in poorer regions due to population increases. This tells us that progress is uneven and that future solutions must fit local realities. More importantly, we identified several natural immune-related markers in the blood-such as C-reactive protein and specific signaling molecules-that appear to directly raise the risk of maternal infections. These findings matter to scientists because they offer clear targets for future research and drug development. For non-scientists, especially public health officials and expectant families, our work shows that monitoring inflammation levels could one day help predict or even prevent these dangerous infections. By linking global patterns with genetic evidence, we provide a more complete roadmap for saving mothers’ lives.

## Introduction

Maternal sepsis and other maternal infections (MSMI) are defined as sepsis occurring during the delivery or postpartum period, along with other infectious conditions closely associated with pregnancy. These encompass genitourinary tract infections (excluding sexually transmitted infections), obstetric surgical site infections, and breast infections related to childbirth and lactation. Such infections pose a serious threat to both maternal and neonatal health [1–2]. On the one hand, obstetric infections can lead to chronic pelvic inflammatory disease, ectopic pregnancy, infertility, and even maternal death. Among these outcomes, puerperal sepsis stands as one of the leading causes of maternal mortality. Global estimates indicate that approximately 10.7% of maternal deaths are attributable to puerperal sepsis [2]. On the other hand, these infections also contribute to short-term neonatal complications, such as intraventricular hemorrhage (IVH), respiratory distress syndrome (RDS), and necrotizing enterocolitis (NEC), and may result in long-term sequelae, including cerebral palsy and intellectual disability. Additionally, they increase the risks of preterm birth and fetal growth restriction. Epidemiological evidence suggests that nearly one million neonatal deaths annually are attributable to maternal infection or sepsis [3]. Although existing studies have delineated certain epidemiological characteristics of maternal sepsis and related infectious diseases, most findings are confined to specific geographic regions and frequently lack age-standardized methodologies, thereby limiting comparability across different countries and settings [4–7]. Furthermore, due to the complex interplay of age, period, and cohort effects, the independent contribution of each factor to morbidity and mortality risks remains poorly understood. Consequently, accurate prediction of MSMI incidence and mortality trends is of particular importance, as it will furnish essential evidence for optimizing the allocation of medical resource, formulating targeted prevention strategies, and implementing effective clinical interventions.

The Global Burden of Diseases, Injuries, and Risk Factors Study 2021 (GBD 2021) provides a standardized and systematic assessment of 369 diseases and injuries and 87 risk factors across 204 countries and territories, thus offering essential data support for global public health research [8]. GBD 2021 data play a central role in numerous studies by facilitating the characterization of disease burden, analysis of temporal trends, future projections, and monitoring of health inequities, thereby forming a critical evidence base for public health policies and intervention strategies [9–10]. Using GBD 2021 data, this study calculated the age-standardized incidence and mortality rates of MSMI among women of reproductive age. Trends from 1990 to 2021 were analyzed, the independent effects of age, period, and birth cohort were evaluated, and the disease burden for the next 25 years was projected. These findings are intended to serve as a comprehensive reference for formulating targeted prevention and control strategies for MSMI at global, regional, and national levels.

Disparities in disease burden provide policymakers with an evidence-based foundation for implementing targeted interventions and preventive measures in specific populations. Nevertheless, patients affected by MSMI, along with their families, often seek to understand the underlying causes of these conditions to adopt effective prevention strategies. Therefore, it is essential to thoroughly investigate the risk factors contributing to MSMI for developing effective interventions aimed at reducing associated risks. Unlike traditional observational studies, which are susceptible to residual confounding and various biases, Mendelian randomization (MR) is an epidemiological approach that uses genetic variants-randomly allocated at conception-as instrumental variables to infer causal relationships between exposures and outcomes [11]. This method substantially reduces reverse causation and confounding biases often introduced by sociodemographic or behavioral factors in conventional observational studies. In this study, we applied a two-sample MR design to examine bidirectional causal relationships between various potential risk factors and MSMI. Furthermore, multivariable MR was employed to assess the independent effects of multiple significantly associated exposures on MSMI outcomes. The findings reveal causal links between several modifiable factors and MSMI, which may inform future preventive strategies and clinical guidance.

## Materials and methods

### Ethics statement

The FinnGen study protocol was approved by Coordinating Ethics Committee of the Hospital District of Helsinki and Uusimaa (number HUS/990/2017), with all participants providing written informed consent [14].

### Overview and global data sources

**Fig 1:**
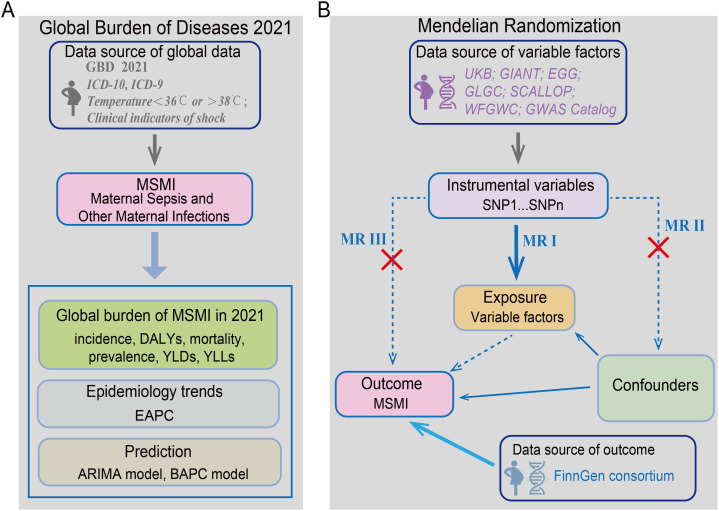
Study Overview: Global Burden Analysis and Mendelian Randomization Design. This flowchart outlines our two-pronged analytical approach. The Global Burden of Disease assessment was summarized in A, quantifying the worldwide impact of MSMI through metrics including incidence, DALYs, mortality, prevalence, YLDs, YLLs and epidemiology trends using EAPC. The MR framework was summarized in B, where genetic variants SNPs significantly associated with exposure phenotypes serve as instrumental variables **(IVs)**. The MR design rests on three core assumptions: **(I)** Relevance: SNPs must strongly associate with the exposure; **(II)** Independence: SNPs must be independent of exposure-outcome confounders; and **(III)** Exclusion restriction: SNPs must affect the outcome exclusively through the exposure, without alternative pathways. YLDs, Years Lived with Disability; DALYs, Disability-Adjusted Life Years; YLLs, Years of Life Lost. Methodological abbreviations: EAPC, Estimated Annual Percentage Change; ARIMA, autoregressive integrated moving average; BAPC, Bayesian age-period-cohort.

A schematic overview of the study design is shown in Fig 1. Data pertaining to MSMI were obtained from the Global Health Data Exchange (GHDx) data query tool (https://vizhub.healthdata.org/gbd-results/). Case definitions for MSMI were aligned with the diagnostic criteria set forth in the International Classification of Diseases, Ninth and Tenth Revisions (ICD-9 and ICD-10) [12]. As previously defined [12], MSMI comprises two distinct entities [12]. Maternal sepsis is characterized by abnormal core body temperature (＜36°C or >38°C) along with signs of shock, such as systolic hypotension (<90 mmHg) and tachycardia (>120 beats per minute). Other maternal infections refer to any non-AIDS-defining and non-sexually transmitted infectious conditions not considered epidemiologically linked to pregnancy. The latter category encompasses disorders such as gestational urinary tract infections, mastitis, candidiasis, and bacterial vaginosis.

We sourced comprehensive global data on health losses attributable to MSMI between 1990 and 2021, spanning 204 countries and territories and 21 regions. Key metrics included incidence, prevalence, mortality, years lived with disability (YLDs), years of life lost (YLLs), and disability-adjusted life years (DALYs), along with their corresponding age-standardized rates (ASIR, ASPR, ASMR, AS-YLD, AS-YLL, and ASDR). DALYs were derived as the sum of YLDs and YLLs. YLDs were calculated by multiplying the prevalence of each disease sequela by its disability weight, while YLLs were estimated based on cause-specific deaths multiplied by the standard life expectancy at the age of death [12]. Although global standardization efforts under the GBD framework have enhanced data comparability, disparities in socioeconomic status and variations in reproductive-age population structures may still influence data quality. To address potential confounding from these factors, stratified analyses of MSMI-related health loss were conducted by Socio-demographic Index (SDI) and by maternal age group. The SDI is a composite measure of sociodemographic development that integrates income, educational attainment, and fertility rates [12]. Based on 2021 SDI quintiles, all countries and territories were classified into five development strata: low, low-middle, middle, high-middle, and high, where elevated SDI reflects advanced socioeconomic conditions [12]. Additionally, temporal trends in MSMI burden were evaluated across detailed age categories as defined in GBD 2021: 10–14, 15–19, 20–24, 25–29, 30–34, 35–39, 40–44, 45–49, and 50–54 years [13]. Complete datasets supporting these analyses are available in S1-S6 Tables.

### MR study design

A two-sample MR analysis was employed to assess the potential causal effects of multiple exposures on MSMI. The analysis was grounded in three core MR assumptions: (i) the genetic instruments exhibit strong associations with the exposures of interest; (ii) these instruments are independent of potential confounders; and (iii) they influence the risk of MSMI only via the exposure pathways (Fig 1). As all genetic and outcome data were derived from previously published studies with ethical approvals [13], no additional informed consent was required for the present study.

### MR date sources

Genome-wide association study (GWAS) summary statistics for MSMI were obtained from the FinnGen Consortium [14]. The analyzed variable factors encompassed five major categories:

- **Basic characteristics**: birth weight, body mass index (BMI);
- **Medical history:** previous smoking status, history of stillbirth, spontaneous miscarriage or termination, number of pregnancy terminations, medication use for cholesterol, blood pressure, diabetes, or exogenous hormones (e.g., cholesterol-lowering medication);
- **Routine blood and serum metabolic biomarkers:** high-density lipoprotein (HDL) cholesterol, low-density lipoprotein (LDL) cholesterol, triglycerides (TG), total cholesterol levels or total cholesterol (TC), C-reactive protein levels or C-reactive protein (CRP), vitamin D levels (VitD), N-terminal prohormone of brain natriuretic peptide levels (NT-proBNP);
- **Serum inflammatory cytokines:** RANTES levels (RANTES), interleukin-13 levels (IL-13), interleukin-10 levels (IL-10), interleukin-1 receptor antagonist levels (IL-1Ra), interleukin-2 receptor antagonist levels (IL-2Ra), interferon gamma levels (IFN-γ);
- **Serum-specific biomarkers:** Pregnancy-specific beta-1-glycoprotein 11 (PSG11), Pregnancy-specific beta-1-glycoprotein 9 (PSG9).

Instrumental variables for these factors were sourced from multiple large-scale genetic consortia and databases, including the UK Biobank (UKB) [15], the Early Growth Genetics (EGG) Consortium [16], the Genetic Investigation of Anthropometric Traits (GIANT) Consortium [17], the Global Lipids Genetics Consortium (GLGC) [18], the NHGRI-EBI Genome-Wide Association Studies Catalog (GWAS Catalog) [19], the Systematic and Combined Analysis of Olink Proteins (SCALLOP) [20], and the Within Family GWAS Consortium (WFGWC) [21]. Detailed data source descriptions are provided in Table 1.

**Table 1:** Data source of variable factors.

id.exposure	Exposure	Date source	Population
ieu-a-27	Birth weight	EGG	European
ebi-a-GCST90095034	Body mass index	GWAS Catalog	Hispanic or Latin American
ieu-a-94	Body mass index	GIANT	European
ieu-b-4815	Body mass index	Within family GWAS consortium	European
ukb-a-224	Smoking status: Previous	UBK	European
ukb-b-12621	Ever had stillbirth, spontaneous miscarriage or termination	UBK	European
ukb-a-355	Number of pregnancy terminations	UBK	European
ukb-a-448	Medication for cholesterol blood pressure diabetes or take exogenous hormones: Cholesterol lowering medication	UBK	European
ukb-b-17805	Medication for cholesterol blood pressure diabetes or take exogenous hormones: Cholesterol lowering medication	UBK	European
ukb-b-18009	Medication for cholesterol, blood pressure, diabetes, or take exogenous hormones: Blood pressure medication	UBK	European
ebi-a-GCST000755	HDL cholesterol	GWAS Catalog	European
ebi-a-GCST000759	LDL cholesterol	GWAS Catalog	European
ebi-a-GCST005068	LDL cholesterol	GWAS Catalog	European
ebi-a-GCST90018741	LDL cholesterol	GWAS Catalog	East Asian
ieu-a-781	LDL cholesterol	GLGC	Mixed
ieu-b-4845	LDL cholesterol	Within family GWAS consortium	European
ebi-a-GCST000758	Triglycerides	GWAS Catalog	European
ieu-a-783	Triglycerides	GLGC	Mixed
ieu-b-4849	Triglycerides	Within family GWAS consortium	European
ieu-b-4850	Triglycerides	Within family GWAS consortium	European
ebi-a-GCST90018754	Total cholesterol levels	GWAS Catalog	East Asian
ebi-a-GCST90101747	Total cholesterol levels	GWAS Catalog	Sub-Saharan African
ieu-a-782	Total cholesterol	GLGC	Mixed
ebi-a-GCST005067	C-reactive protein levels	GWAS Catalog	European
ebi-a-GCST90018730	C-reactive protein	GWAS Catalog	East Asian
ebi-a-GCST005367	Vitamin D levels	GWAS Catalog	European
ebi-a-GCST90012082	N-terminal prohormone brain natriuretic peptide levels	GWAS Catalog	European
ebi-a-GCST004431	RANTES levels	GWAS Catalog	European
ebi-a-GCST004443	Interleukin-13 levels	GWAS Catalog	European
ebi-a-GCST004444	Interleukin-10 levels	GWAS Catalog	European
ebi-a-GCST004447	Interleukin-1-receptor antagonist levels	GWAS Catalog	European
ebi-a-GCST004454	Interleukin-2-receptor antagonist levels	GWAS Catalog	European
ebi-a-GCST004456	Interferon gamma levels	GWAS Catalog	European
prot-a-2400	Pregnancy-specific beta-1-glycoprotein 11	SCALLOP	European
prot-a-2408	Pregnancy-specific beta-1-glycoprotein 9	SCALLOP	European

### Instrumental variable selection

Based on GWAS summary statistics, single-nucleotide polymorphisms (SNPs) associated with each exposure of interest were selected as instrumental variables. Independent SNPs were identified through a clumping procedure under strict linkage disequilibrium (LD) criteria (*P*  <  5  ×  10 ⁻ ⁸, *r*²  <  0.001, clumping window  =  10,000 kb). For unavailable SNPs in the outcome dataset, proxy variants were identified from the 1000 Genomes European reference panel using a high LD threshold (*r*²  >  0.8), excluding SNPs without suitable proxies. Additionally, palindromic SNPs with intermediate allele frequencies were removed to prevent strand ambiguity. To enhance analytical robustness, SNPs with a minor allele frequency (MAF) < 0.01 were further excluded, thereby minimizing potential bias arising from low-frequency variants that often yield unreliable estimates in GWAS. The proportion of variance explained (R^2^) for each risk factor by individual SNPs was quantified [13], and the strength of instrumental variables was evaluated using the F-statistic [22]. The F-statistic was calculated as F = R² × (N − 2) / (1 − R²), where R² represents the proportion of variance explained by the genetic instruments and N is the sample size.

### Statistical analysis

Spearman’s rank correlation analysis was employed to investigate the association between the Socio-demographic Index (SDI) and the burden of MSMI. The SDI is a composite indicator of a region’s development level, ranging from 0 (lowest) to 1 (highest), based on lag-distributed income per capita, average educational attainment, and total fertility rate. Given the non-normal distribution of burden indicators across countries, Spearman’s rank correlation was selected as it is a non-parametric method that does not assume linearity or normality, and is robust to outliers. All correlation analyses were performed at the country level, including all 204 countries and territories in the GBD study for the year 1990 and 2021. For each pair of variables (SDI vs. each burden indicator), Spearman’s correlation coefficient (r) and the corresponding two-tailed p-value were calculated. A p-value < 0.05 was considered statistically significant. All statistical analyses were conducted using R software (version 4.5.3) with the “cor.test” function [12]. Decomposition analysis was performed to quantify the contributions of demographic factors and epidemiological changes to the disparities in the burden of MSMI across different SDI quintiles in 2021. Using the Das Gupta decomposition method, the difference in outcome measure between each SDI quintile (low, low-middle, middle, high-middle, and high SDI) and the global average was partitioned into three components: (1) population structure (aging), reflecting differences in the age composition of populations; (2) population size (growth), reflecting differences in total population across SDI quintiles; and (3) epidemiological changes, reflecting differences in age-specific rates of the outcome measure. Absolute contributions (the number of cases attributable to each factor) and relative contributions (the percentage of the total disparity explained by each factor) were calculated for each SDI quintile. All decomposition analyses were conducted using R software (version 4.5.3) with the “**Dasgupta”** function from the “**decompose”** package [12].

The Estimated Annual Percentage Change (EAPC) serve as a key indicator for quantifying temporal trends in epidemiological metrics-such as incidence, mortality, or DALYs-over a specified period. It reflects the average annual rate of change, expressed as a percentage. In this study, the EAPC was applied to analyze trends in the ASR of MSMI from 1990 to 2021, based on data from the GBD 2021 database. EAPC was derived using a linear regression model applied to the natural logarithm of ASR values collected over consecutive years. The use of ASR mitigates confounding from temporal shifts in population age structure over time. The regression model is specified as: **ln (ASR) = α + β × year + ε**, where **ln (ASR)** is the natural log-transformed ASR, **year** represents the time variable, **α** is the intercept, **β** is the regression coefficient for time, and **ε** denotes the random error term. EAPC is calculated using the estimated slope **β** with the formula: **EAPC = (e^β - 1) × 100**. The 95% confidence interval (CI) is obtained from the standard error (SE) of **β**: **Upper limit = {exp** (β + **1.96 × SE_**β**) - 1} × 100, Lower limit = {exp (**β - **1.96 × SE_**β**) - 1} × 100.** A statistically significant increasing trend in ASR is concluded if both the EAPC and the lower limit of its 95% CI exceed zero, whereas a decreasing trend is inferred when the EAPC and the upper limit of the 95% CI are below zero.

To project future disease burden, two complementary modeling approaches were employed, including the autoregressive integrated moving average (ARIMA) model and the Bayesian age-period-cohort (BAPC) model. As a well-established method for epidemiological forecasting [23], the ARIMA model was fitted to historical time-series data from GBD 2021. After ensuring stationarity through differencing, the “auto.arima()” function was used to identify the optimal model configuration based on the lowest Akaike Information Criterion (AIC) and Bayesian Information Criterion (BIC). Model adequacy was confirmed via white noise testing (P > 0.05 for residuals). The “forecast” package was employed to predict future MSMI-related metrics at α = 0.05 significance. Simultaneously, the BAPC model captured complex temporal structures by integrating age, period, and cohort effects through Bayesian methodology, INLA algorithm for efficient prior and posterior distribution updating. A log-linear regression model was fitted for MSMI metrics as follows: **ln (ASR) = α + β_age × age + β_period × period + β_cohort × cohort + ε**, with Bayesian parameter estimated via the integrated nested Laplace approximation (INLA) algorithm for efficient prior and posterior distribution updating [24].

A two-sample MR framework was applied to investigate the causal relationships between variable factors and MSMI. After harmonizing effect alleles and directions using the “**harmonise_data**” function from the TwoSampleMR package in R software (version 4.1.0), MR analyses were conducted through the “**mr**” function, incorporating five complementary methods: MR-Egger regression (MR-Egger) [25], weighted median (WM) [25], inverse-variance weighted (IVW) [26], simple mode, and weighted mode [26]. MR analyses were performed separately for each exposure factor, with the IVW method serving as the primary analytical approach for causal inference.

Heterogeneity among instrumental variables was assessed using Cochran’s Q test via the “**mr_heterogeneity**” function. P-values > 0.05 indicated absence of significant heterogeneity, supporting the use of fixed-effect inverse-variance weighted (IVW) estimates. In this study, all Q test results were non-significant (P > 0.05), confirming homogeneity across variables [27]. Horizontal pleiotropy was evaluated using MR-Egger regression and MR-PRESSO through the “**mr_pleiotropy_test**” function. P > 0.05 in both tests indicated no evidence of directional pleiotropy, thereby ensuring causal estimate reliability [25,28]. Finally, causal directionality was verified using MR-Steiger analysis, which confirmed the presumed direction (exposure → outcome) by demonstrating greater variance explained in exposure than outcome. Sensitivity analysis was conducted using the “**mr_leaveoneout**” function, which removes each SNP one by one and evaluates its impact on the overall results. Notably, if the results change significantly after removing a particular SNP, this indicates that the SNP is sensitive. In this study, no SNPs with significant deviation were found, indicating that the analysis results are stable and reliable.

Given that genetic variations may influence multiple correlated exposures, multivariable Mendelian randomization (MVMR) was performed to delineate the direct causal effects of individual variable factors on MSMI. This approach isolates the independent contribution of each exposure to disease risk [29]. To mitigate potential collinearity among exposures, the least absolute shrinkage and selection operator (LASSO) was applied for variable selection and dimensionality reduction. This method effectively identified a subset of influential exposures by retaining factors significantly associated with MSMI while excluding those with negligible effects [30]. The optimal tuning parameter (λ) was determined using 10-fold cross-validation, selecting the value that minimized the mean cross-validated error. Variables with non-zero coefficients were retained for subsequent multivariable MR analysis.

## Results

### The global and regional burden of MSMI in 2021

**Fig 2:**
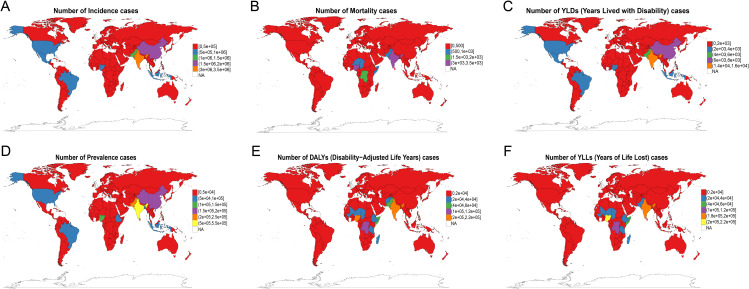
Global Burden of MSMI in 2021. Spatial distribution of MSMI case burden across 204 countries and territories is presented as follows: **(A)** Incidence cases, **(B)** Mortality cases, **(C)** YLDs, **(D)** Prevalence cases, **(E)** DALYs, and **(F)** YLLs. These maps visualize geographical disparities in disease impact, highlighting regions with the highest burden across different health metrics. YLDs, Years Lived with Disability; DALYs, Disability-Adjusted Life Years; YLLs, Years of Life Lost. The map layer (country/region boundaries) was obtained from Natural Earth (https://www.naturalearthdata.com/), which is in the public domain (Terms of use:https://www.naturalearthdata.com/about/terms-of-use/). The basemap was created using the “rnaturalearth” package in R software. Map boundaries are only for illustrative purposes and do not imply any political statement.

**Fig 3:**
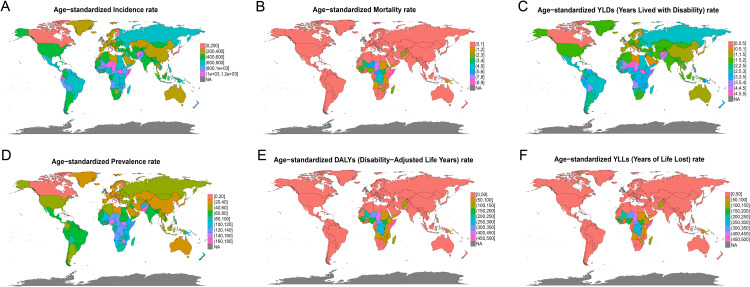
Global Age-Standardized Burden of MSMI in 2021. Geographic distribution of age-standardized MSMI burden metrics across 204 countries and territories: **(A)** Age-Standardized Incidence Rate (ASIR), **(B)** Age-Standardized Mortality Rate (ASMR), **(C)** Age-Standardized YLDs Rate (AS-YLD), **(D)** Age-Standardized Prevalence Rate (ASPR), **(E)** Age-Standardized DALYs Rate (ASDR), and **(F)** Age-Standardized YLLs Rate (AS-YLL). These maps enable cross-national comparisons of disease burden, independent of population age structure differences. YLDs, Years Lived with Disability; DALYs, Disability-Adjusted Life Years; YLLs, Years of Life Lost. The map layer (country/region boundaries) was obtained from Natural Earth (https://www.naturalearthdata.com/), which is in the public domain (Terms of use:https://www.naturalearthdata.com/about/terms-of-use/). The basemap was created using the “rnaturalearth” package in R software. Map boundaries are only for illustrative purposes and do not imply any political statement.

Globally, the country-specific burden of MSMI in 2021 is detailed in Figs 2–3 and 5. Fig 2 presents the absolute numbers of incidence, prevalence, mortality, DALYs, YLDs, and YLLs for each country in 2021. The findings reveal that China, India, and Pakistan reported the highest number of incidence cases and prevalence cases, each exceeding 1,000,000 incidence cases and 150,000 prevalence cases. In terms of mortality attributable to MSMI, India, Nigeria, and the Democratic Republic of the Congo recorded the highest mortality, each experiencing over 1,500 annual deaths. Countries with elevated mortality rates were largely concentrated in Asia and Africa. India, Nigeria, and the Democratic Republic of the Congo also reported the highest number of DALY cases. Furthermore, the assessment of ASR metrics revealed that the highest values for ASIR, ASMR, ASDR, and ASPR in 2021 were predominantly observed in African and South American countries (Fig 3). Within Asia, Afghanistan and Pakistan shouldered the most substantial MSMI burden, whereas the ASR metrics of other Asian countries were generally comparable to those of Europe, North America, and Oceania. At the regional level, Western Sub-Saharan Africa was observed to have the highest number of mortality, DALYs and YLLs in 2021, followed by Sourth Asia, Eastern Sub-Saharan Africa and Central Latin America, whereas Sourth Asia reported the highest number of YLDs, prevalence and incidence in 2021, followed by Western Sub-Saharan Africa, Eastern Sub-Saharan Africa and Central Latin America (Fig 5A). In contrast, six key metrics via age-standardized exhibited different regional trends. Specifically, Eastern Sub-Saharan Africa reported the highest age-standardized rates for mortality, DALYs and YLLs, Andean Latin America exhibited the highest age-standardized rates for YLDs and incidence, and Central Sub-Saharan Africa demonstrated the highest age-standardized rates for prevalence (Fig 5B).

**Fig 4:**
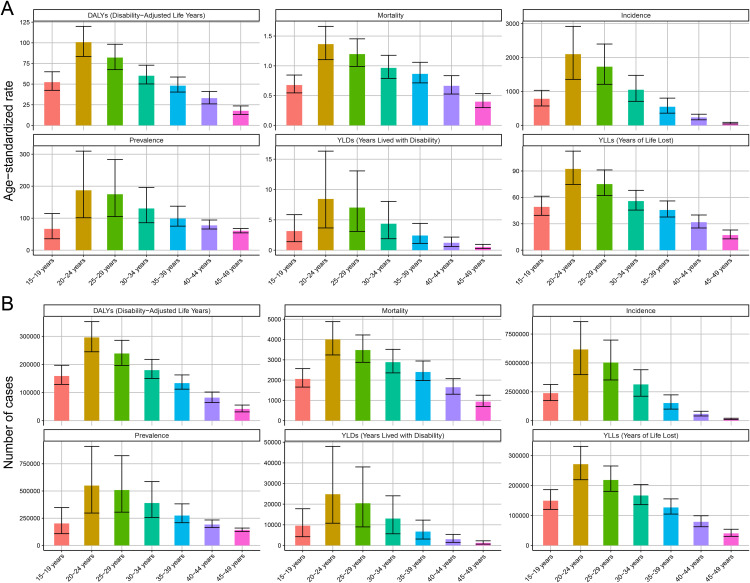
Age-Specific Burden of MSMI in 2021. **(A)** Age-stratified patterns of MSMI burden expressed as ASR, including Age-Standardized DALYs Rate, Age-Standardized Mortality Rate, Age-Standardized Incidence Rate, Age-Standardized Prevalence Rate, Age-Standardized YLDs Rate, and Age-Standardized YLLs Rate, highlighting variations in disease impact across the lifespan. **(B)** Absolute case numbers of MSMI burden, namely DALYs, Mortality, Incidence, Prevalence, YLDs and YLLs, across different age groups, representing the actual population-level disease distribution. DALYs, Disability-Adjusted Life Years; YLDs, Years Lived with Disability; YLLs, Years of Life Lost.

Assessment of the MSMI burden across different age groups in 2021 (Fig 4) revealed a progressive decline in all ASR metrics with advancing age, with the notable exception of the 15–19 age group. The highest burden was observed in the 20–24 age group, whereas the lowest values for all burden metrics were recorded in the 45–49 age group. Furthermore, given that the economic impact of disease varies with socioeconomic development, populations were stratified by the SDI to evaluate the MSMI burden across different development strata. As shown in Fig 6, a decline in the SDI was associated with a gradual increase in the ASIR, ASPR, ASMR, ASDR, AS-YLD, and AS-YLL. Further Spearman correlation analysis showed that SDI was significantly negatively correlated with ASIR, ASPR, ASMR, ASDR, AS-YLD, and AS-YLL (all P < 0.001), whether for 1990 (S1 Fig) or 2021(S2 Fig). Consequently, these results demonstrate a clear negative correlation between SDI and MSMI burden, indicating that the majority of the MSMI burden is concentrated in low-SDI and low-middle-SDI countries and territories.

Decomposition analysis of changes in disease burden among MSMI revealed marked heterogeneity across SDI quintiles (Fig 6C), as well as similar trends across Cause (S3 Fig) and Sex (S4 Fig). All of the total change in MSMI burden, including incidence, prevalence, mortality, DALYs, YLDs and YLLs, was predominantly driven by epidemiological change and population growth (S3 and S4 Figs). For SDI quintiles, distinct patterns were observed for mortality and morbidity indicators (incidence) (Fig 6C). The total change in DALYs among MSMI was predominantly driven by epidemiological change and population growth in lower SDI settings, with opposing effects across the development spectrum. In the Low SDI quintile, population growth contributed substantially to increased DALYs, while epidemiological change exerted a modest negative effect. Conversely, in High SDI and High-middle SDI quintiles, epidemiological change was the dominant driver of DALY reduction, offsetting small increases attributable to aging. The net change (black dots) demonstrated a gradient from high burden in Low SDI to minimal change in High SDI settings. A similar pattern was observed for mortality and YLLs. In Low SDI countries, mortality change was driven primarily by population growth with minimal epidemiological improvement. In contrast, High SDI quintiles experienced mortality reductions through favorable epidemiological change. Notably, aging contributed negligibly to mortality changes across most quintiles except Low SDI, where a marginal aging effect was observed. The decomposition of incidence revealed distinct dynamics. Low SDI countries exhibited the largest increase in incidence cases, driven overwhelmingly by population growth and partially offset by epidemiological improvement. This pattern was attenuated at higher SDI levels, where epidemiological change contributed more substantially to incidence reduction. Aging effects on incidence were minimal across all quintiles.

Across all disease burden metrics, three consistent patterns emerged: (1) population growth was the dominant driver of increased burden in Low SDI settings; (2) epidemiological change contributed to burden reduction across all SDI levels, with effect magnitude positively correlated with development status; and (3) aging exerted minimal influence on most indicators, with slight positive contributions to mortality, incidence and YLDs in lower SDI settings. The net change in disease burden (black dots) demonstrated a clear SDI gradient, with Low SDI countries experiencing the largest change in all metrics and High SDI countries approaching zero net change or reductions.

## Global trends in the burden of MSMI from 1990 to 2021

The global burden trends of MSMI from 1990 to 2021 is presented in Fig 7. The findings reveal a consistent overall decline in all ASR, including ASIR, ASPR, ASMR, AS-YLD, AS-YLL, and ASDR, over this period. By 2021, each of these ASR metrics showed a substantial decrease compared to its 1990 levels. A parallel reduction was observed in the absolute numbers of incidence, prevalence, mortality, YLLs, YLDs, and DALYs, which were all significantly lower in 2021 than in 1990.

As shown in Fig 8, the age distribution of MSMI burden from 1990 to 2021 revealed a general decline in ASR (ASIR, ASMR and ASDR) with advancing age, except in the 15–19 age group. The highest values for ASIR, ASMR, ASPR, and ASDR were consistently observed in the 20–24 age group, while all metrics remained at relatively low levels beyond age 40. Furthermore, compared with 1990, all ASR metrics in 2021showed significant reductions among individuals aged 15–29 years. In contrast, only minimal changes or gradual declines were observed for the 30–49 age group.

Despite consistent diagnostic criteria for MSMI across countries, disparities in socioeconomic development have led to incomplete uniformity in diagnostic quality, potentially affecting the accuracy of global burden assessments. To address this, health losses attributable to MSMI from 1990 to 2021 were further analyzed by SDI, as shown in Fig 9. The study found that, although all ASR indicators exhibited a gradual downward trend in low-SDI regions, the absolute numbers of incidence cases, prevalence cases, and YLDs rose significantly. Specifically, these indicators increased from 3,061,689 (95% UI 2,301,567-4,006,663), 353,651 (95% UI: 239,741–520,932), and 12,526 (95% UI: 5,919–22,393) in 1990–4,711,030 (95% UI: 3,557,613–6,151,574), 597,803 (95% UI: 423,736–852,979), and 19,630 (95% UI: 9,178–35,332) in 2021, representing increases of 54%, 69%, and 57%, respectively. In contrast, mortality rate, YLLs, and DALYs remained largely unchanged in this region. Population growth was identified as the primary driver of this phenomenon; approximately 70%–75% of the increase in MSMI cases in low-SDI regions was attributable to population growth. Meanwhile, the burden of MSMI remained consistently low in high-SDI and high-middle-SDI regions over the study period.

**Fig 5:**
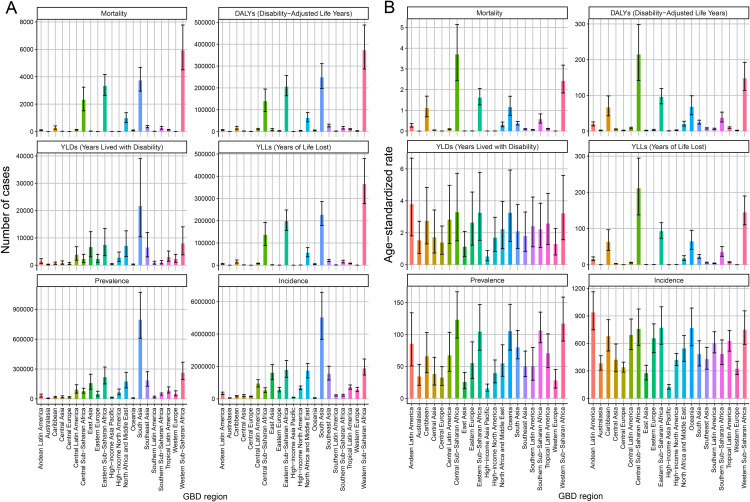
Regional Burden of MSMI in 2021. **(A)** Absolute number of MSMI cases by region, representing the actual magnitude of disease burden across different populations. **(B)** Age-standardized burden rates of MSMI across geographic regions, facilitating direct comparison of disease intensity while accounting for population age structures. 21 GBD regions: Andean Latin America, Australasia, Caribbean, Central Asia, Central Europe, Central Latin America, Central Sub−Saharan Africa, East Asia, Eastern Europe, Eastern Sub−Saharan Africa, High−income Asia Pacific, High−income North America, North Africa and Middle East, Oceania, South Asia, Southeast Asia, Southern Latin America, Southern Sub−Saharan Africa, Tropical Latin America, Western Europe and Western Sub−Saharan Africa. DALYs, Disability-Adjusted Life Years; YLDs, Years Lived with Disability; YLLs, Years of Life Lost.

**Fig 6:**
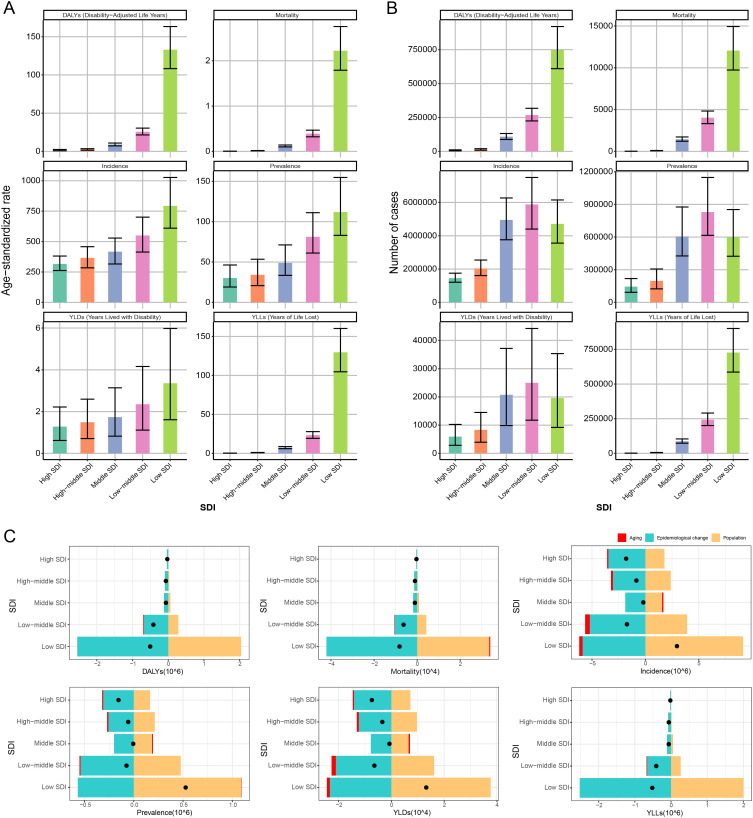
Burden of MSMI by SDI in 2021. **(A)** Age-standardized burden rates of MSMI across SDI categories, revealing gradients in disease intensity relative to socioeconomic development levels. **(B)** Absolute number of MSMI cases by SDI stratum, demonstrating the actual distribution of disease burden across populations with varying socioeconomic status. **(C)** Decomposition results of MSMI burden across SDI categories at the SDI components level. Three SDI components include population structure (aging), epidemiological changes, and population size (population). DALYs, Disability-Adjusted Life Years; YLDs, Years Lived with Disability; YLLs, Years of Life Lost.

**Fig 7:**
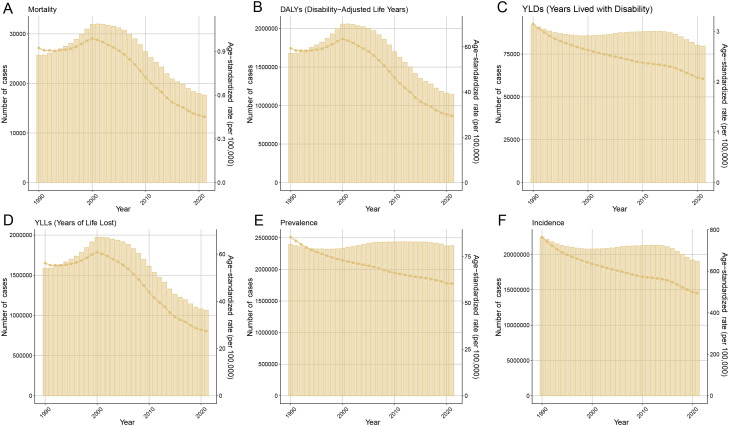
Temporal Trends in MSMI Burden from 1990 to 2021. **(A)** Mortality counts and ASMR. **(B)** DALYs and ASDR. **(C)** YLDs and AS-YLD. **(D)** YLLs and AS-YLL. **(E)** Prevalence counts and ASPR. **(F)** Incidence counts and ASIR. The bar chart represents the absolute cases, and the dotted line chart stands for age-standardized burden rates. DALYs, Disability-Adjusted Life Years; YLDs, Years Lived with Disability; YLLs, Years of Life Lost. ASMR, Age-Standardized Mortality Rate; ASDR, Age-Standardized DALYs Rate; AS-YLD, Age-Standardized YLDs Rate; AS-YLL, Age-Standardized YLLs Rate; ASIR, Age-Standardized Incidence Rate; ASPR, Age-Standardized Prevalence Rate.

**Fig 8:**
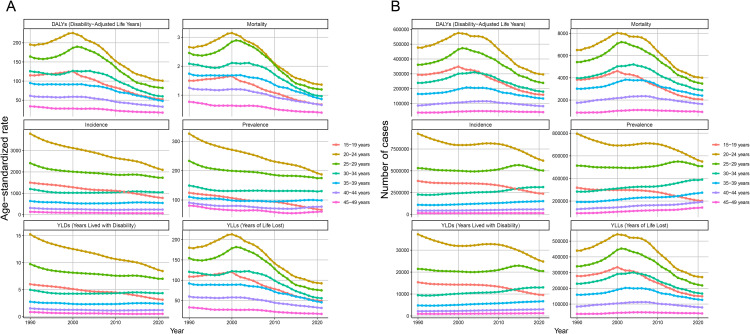
Age-Stratified Burden Trends of MSMI from 1990 to 2021. **(A)** Temporal patterns in age-standardized burden rates across age groups, showing how disease intensity has evolved over three decades while controlling for demographic changes. **(B)** Longitudinal trends in absolute case numbers by age group, revealing shifts in the actual population distribution of MSMI burden across different generations. DALYs, Disability-Adjusted Life Years; YLDs, Years Lived with Disability; YLLs, Years of Life Lost.

**Fig 9:**
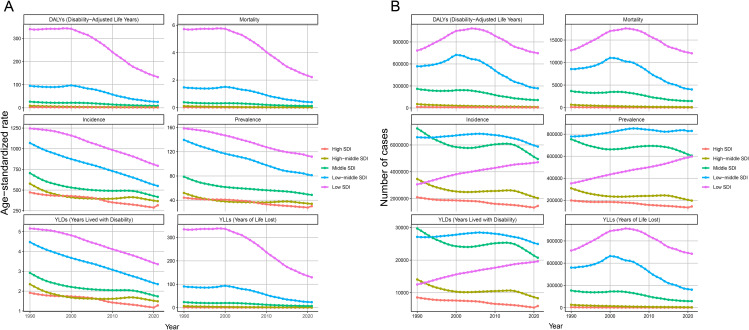
Socioeconomic Disparities in MSMI Burden from 1990 to 2021. **(A)** Trends in age-standardized burden rates across SDI categories, illustrating the evolution of disease intensity in relation to socioeconomic development over three decades. **(B)** Temporal patterns in absolute case numbers by SDI stratum, revealing how the actual distribution of MSMI burden has shifted across populations with varying socioeconomic status. DALYs, Disability-Adjusted Life Years; YLDs, Years Lived with Disability; YLLs, Years of Life Lost.

**Fig 10:**
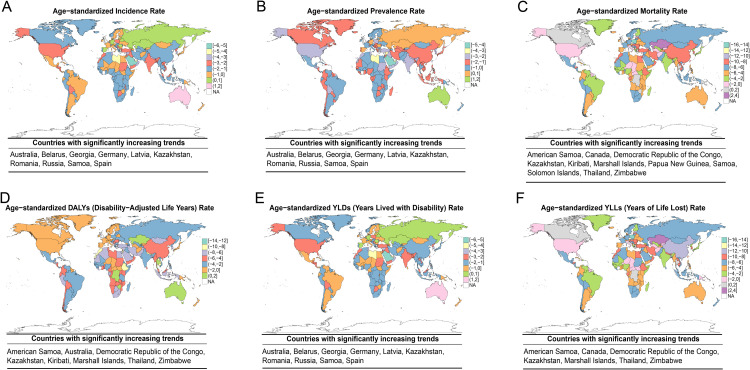
Global Trends in Age-Standardized Burden of MSMI from 1990 to 2021. Spatiotemporal patterns of EAPC across 204 countries and territories are shown for: **(A)** ASIR, **(B)** ASPR, **(C)** ASMR, **(D)** ASDR, **(E)** AS-YLD, and **(F)** AS-YLL. Countries with significantly increasing trends were highlighted below each small chart. These maps visualize geographical heterogeneity in long-term trends, highlighting regions with accelerating or decelerating disease burden. ASIR, Age-Standardized Incidence Rate; ASPR, Age-Standardized Prevalence Rate; ASMR, Age-Standardized Mortality Rate; ASDR, Age-Standardized DALYs Rate; AS-YLD, Age-Standardized YLDs Rate; AS-YLL, Age-Standardized YLLs Rate. The map layer (country/region boundaries) was obtained from Natural Earth (https://www.naturalearthdata.com/), which is in the public domain (Terms of use:https://www.naturalearthdata.com/about/terms-of-use/). The basemap was created using the “rnaturalearth” package in R software. Map boundaries are only for illustrative purposes and do not imply any political statement.

National-level trends in the MSMI burden from 1990 to 2021 revealed divergent patterns across countries, as shown in Fig 10. Globally, the ASIR of MSMI declined in most countries during this period, with the exception of ten nations, including Australia, Belarus, Georgia, Germany, Latvia, Kazakhstan, Romania, Russia, Samoa and Spain. Saudi Arabia demonstrated the most substantial decrease in ASIR. A similar overall downward trend was observed for ASMR worldwide. However, Kazakhstan experienced a significant increase in ASMR, while Canada, the Democratic Republic of the Congo, Zimbabwe, Thailand, and Papua New Guinea exhibited more modest elevations. Furthermore, assessment of trends in the ASDR revealed that all countries and regions displayed a decline in DALYs, with the exception of five countries, including Australia and Kazakhstan. The most substantial reductions in ASDR were observed in Asian and African nations.

### Future trends in the burden of MSMI

**Fig 11:**
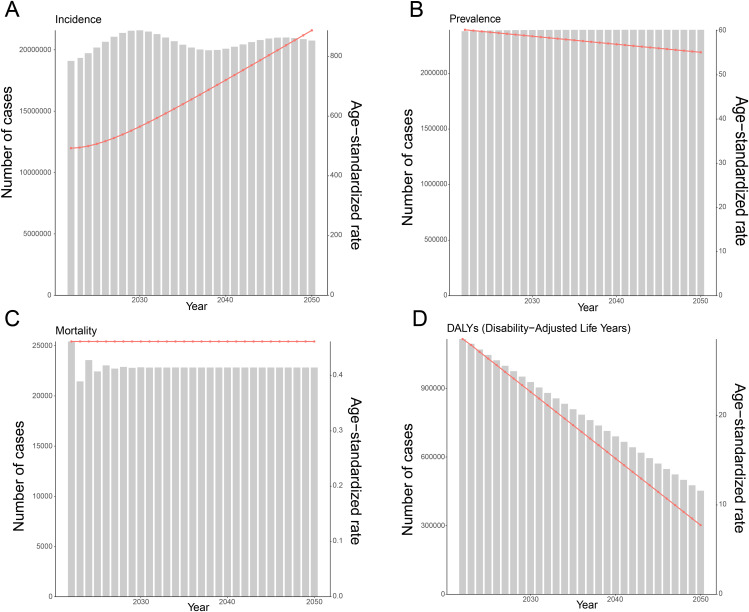
Projected Trends in MSMI Burden from 2022 to 2050 using ARIMA Model. **(A)** Projected trends in incidence counts and ASIR. **(B)** Projected trends in prevalence counts and ASPR. **(C)** Projected trends in mortality counts and ASMR. **(D)** Projected trends in DALYs and ASDR. ARIMA, autoregressive integrated moving average model. The bar chart represents the absolute cases, and the dotted line chart stands for age-standardized burden rates. DALYs, Disability-Adjusted Life Years; ASIR, Age-Standardized Incidence Rate; ASPR, Age-Standardized Prevalence Rate; ASMR, Age-Standardized Mortality Rate; ASDR, Age-Standardized DALYs Rate.

**Fig 12:**
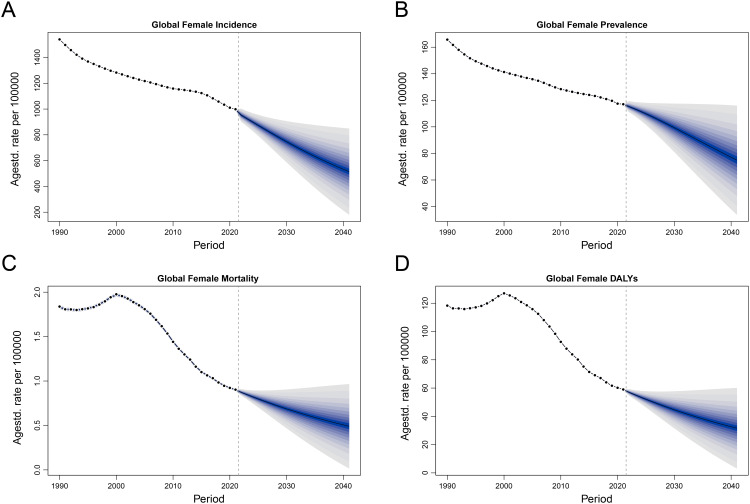
Trend in the burden of MSMI from 2022 to 2041 using BAPC model. **(A)** Trend of incidence and ASIR from 2022 to 2041. **(B)** Trend of prevalence and ASPR from 2022 to 2041. **(C)** Trend of mortality and ASMR from 2022 to 2041. **(D)** Trend of DALYs and ASDR from 2022 to 2050. The blue line represents the predicted trend, and the grey-shaded area represents the 95% confidence interval of the predicted values; the grey-dotted vertical line divides the data into real values (1990-2021) and predicted values (2022-2041). BAPC model, Bayesian age-period-cohort.

To forecast future trends in the burden of MSMI, both the ARIMA and BAPC models were employed. Projections from the ARIMA model suggest that ASPR and ASDR of MSMI will continue to decline in the coming years, while the ASIR is projected to rise, and the ASMR is expected to remain stable (Fig 11). Specifically, by 2050, ASPR and ASDR are estimated to decrease from 60.20 to 55.11 and from 28.57 to 7.74, respectively, whereas ASIR is projected to increase from 492.69 to 887.40. In contrast, projections from the BAPC model differ slightly from those of the ARIMA model. All four metrics, including ASIR, ASPR, ASMR, and ASDR, exhibited a consistent decline through 2041, with the magnitude of reduction illustrated by the blue lines (Fig 12), Based on these findings,it is concluded that ASPR and ASDR are likely to continue decreasing in the future. Nevertheless, the trajectory of ASIR remain uncertain, and ASMR is expected to either remain stable or undergo a slow, sustained decline.

### Two-sample MR analysis and multivariable MR analysis

To investigate potential causal links, a range of variable factors were systematically evaluated in relation to MSMI. For maternal sepsis, multiple factors showed statistically significant associations (*P* < 0.05), as detailed in Table 2. These included lipid profiles, inflammatory markers, medication use, and pregnancy history variables. Using the inverse variance weighted (IVW) method, we identified several factors with odds ratios (OR) > 1, such as LDL cholesterol, previous smoking, RANTES levels, IL-13 levels, IL-10 levels, birth weight, cholesterol-lowering medications, blood pressure medication, and PSG11, indicating their potential roles as risk factors for the development of maternal sepsis. Conversely, those factor with OR < 1, including HDL cholesterol, TG, TC, IL-1Ra levels, IFN-γ levels, PSG9, and number of pregnancy terminations, emerged as potential protective influences against the development of maternal sepsis.

**Table 2:** Results of the relationship between the variable factors and the risk of maternal sepsis.

id.exposure	Exposure	SNPs	Method	P value	OR	Lower 95%CI	Upper 95%CI
ieu-a-27	Birth weight	13	MR Egger	0.026189844	17.61498355	1.970634157	157.4557329
			Weighted median	0.014247002	1.837887361	1.129660277	2.990129
			Inverse variance weighted	0.014684896	1.653291065	1.103972225	2.475942133
			Simple mode	0.081865576	2.246910894	0.974331178	5.181614508
			Weighted mode	0.069196648	2.035202449	1.012728669	4.089988895
ukb-a-224	Smoking status: Previous	151	MR Egger	0.051806864	51.07375485	1.000928611	2606.108373
			Weighted median	0.002474608	4.336763944	1.676947379	11.21533194
			Inverse variance weighted	0.017712179	2.176941596	1.14454347	4.140580797
			Simple mode	0.175262762	8.157792703	0.39782714	167.2826589
			Weighted mode	0.149442164	11.34477924	0.424578392	303.1336929
ukb-a-448	Medication for cholesterol blood pressure diabetes or take exogenous hormones: Cholesterol lowering medication	10	MR Egger	0.169756592	19.87727453	0.40912769	965.7279435
			Weighted median	0.02758256	6.095166926	1.220792874	30.43191081
			Inverse variance weighted	0.00486639	6.209318177	1.741878343	22.13451494
			Simple mode	0.263178546	4.878170969	0.361405876	65.84439704
			Weighted mode	0.137482275	6.53350567	0.684122105	62.39631204
ukb-b-18009	Medication for cholesterol, blood pressure, diabetes, or take exogenous hormones: Blood pressure medication	20	MR Egger	0.748570165	2.801366397	0.005667237	1384.740787
			Weighted median	0.149057338	3.037928239	0.671493829	13.74399525
			Inverse variance weighted	0.038760688	3.432065962	1.065747486	11.05240868
			Simple mode	0.327438317	4.077770878	0.26312739	63.19454362
			Weighted mode	0.413179614	2.861438751	0.243767897	33.5886383
ukb-a-355	Number of pregnancy terminations	28	MR Egger	0.287195296	0.419467804	0.087520754	2.010417297
			Weighted median	0.233074067	0.61861041	0.280920143	1.362233534
			Inverse variance weighted	0.0210607	0.504499724	0.282100298	0.902232198
			Simple mode	0.85021559	1.154661131	0.26328662	5.063843838
			Weighted mode	0.967277967	1.026820841	0.29333479	3.594394788
ebi-a-GCST000755	HDL cholesterol	130	MR Egger	0.065685167	0.8253907	0.674018292	1.010758633
			Weighted median	0.516823887	0.949153684	0.81061175	1.111373868
			Inverse variance weighted	0.005521824	0.882204584	0.807464098	0.963863198
			Simple mode	0.039523617	0.605877579	0.377837325	0.971549435
			Weighted mode	0.977230465	1.003004879	0.81656924	1.232006714
ebi-a-GCST000758	Triglycerides	143	MR Egger	0.009163818	0.771160325	0.63596414	0.935097137
			Weighted median	0.000143682	0.80930529	0.72567046	0.902579186
			Inverse variance weighted	0.000008693679	0.859187247	0.796469044	0.926844215
			Simple mode	0.023429635	0.712645161	0.533345432	0.952221758
			Weighted mode	0.016152045	0.74230209	0.58396573	0.943569742
ieu-a-783	Triglycerides	165	MR Egger	0.026624249	0.811708821	0.676127916	0.974477159
			Weighted median	0.006503945	0.868133426	0.784066002	0.961214544
			Inverse variance weighted	0.00012649	0.87085792	0.811411194	0.934659915
			Simple mode	0.024788914	0.707683813	0.524721496	0.954442278
			Weighted mode	0.04316658	0.712038099	0.513623233	0.987101482
ieu-b-4850	Triglycerides	164	MR Egger	0.514636128	0.950092268	0.814772503	1.10788633
			Weighted median	0.119103355	0.932661869	0.854377866	1.018118793
			Inverse variance weighted	0.005936672	0.914919292	0.858760057	0.974751101
			Simple mode	0.874969231	1.019541065	0.801451326	1.296977059
			Weighted mode	0.677463558	1.040494333	0.863268529	1.254103932
ebi-a-GCST005068	LDL cholesterol	10	MR Egger	0.542652878	1.105400094	0.811641127	1.505479857
			Weighted median	0.067559961	1.174462584	0.988448546	1.395482211
			Inverse variance weighted	0.004129546	1.222608152	1.065704536	1.402612677
			Simple mode	0.282342085	1.156885747	0.9011695	1.485164146
			Weighted mode	0.222616731	1.166143322	0.92658473	1.467637232
ebi-a-GCST90101747	Total cholesterol levels	8	MR Egger	0.35339293	0.788616958	0.496436891	1.25276086
			Weighted median	0.03148423	0.727618402	0.544582631	0.972173016
			Inverse variance weighted	0.01640527	0.753491828	0.597975198	0.949453986
			Simple mode	0.08750503	0.672150117	0.454072204	0.994964624
			Weighted mode	0.060587159	0.715926922	0.534015199	0.959806684
ebi-a-GCST004431	RANTES levels	121	MR Egger	0.311158283	1.065319734	0.943031004	1.203466409
			Weighted median	0.00018884	1.140730975	1.064543343	1.222371232
			Inverse variance weighted	0.000002851483	1.125730823	1.071270839	1.182959378
			Simple mode	0.068437547	1.222763384	0.986815698	1.515126175
			Weighted mode	0.068815772	1.207212398	0.987374804	1.47599652
ebi-a-GCST004443	Interleukin-13 levels	139	MR Egger	0.471625071	1.035113669	0.942521091	1.136802474
			Weighted median	0.108217887	1.061069258	0.987030259	1.140662062
			Inverse variance weighted	0.039725047	1.045933268	1.00211159	1.091671238
			Simple mode	0.842634635	1.020647571	0.834471163	1.248361249
			Weighted mode	0.040195098	1.119939023	1.006104332	1.246653429
ebi-a-GCST004444	Interleukin-10 levels	7	MR Egger	0.193901758	1.254133972	0.932914526	1.685955119
			Weighted median	0.004550546	1.258570964	1.073695067	1.475280013
			Inverse variance weighted	0.002377289	1.229558639	1.076110241	1.404888077
			Simple mode	0.057193262	1.331528912	1.048481215	1.690988085
			Weighted mode	0.024490583	1.270998084	1.085806519	1.48777531
ebi-a-GCST004447	Interleukin-1-receptor antagonist levels	105	MR Egger	0.025021545	0.864016141	0.761756259	0.980003619
			Weighted median	0.075208055	0.925423377	0.849689706	1.007907264
			Inverse variance weighted	0.035943042	0.93825573	0.884011244	0.995828752
			Simple mode	0.484748145	0.917567495	0.721443909	1.167007022
			Weighted mode	0.388876835	0.907349869	0.727993942	1.130893731
ebi-a-GCST004456	Interferon gamma levels	121	MR Egger	0.74627903	0.975794177	0.841476345	1.131552042
			Weighted median	0.001997875	0.840715698	0.753116693	0.938503809
			Inverse variance weighted	0.000132407	0.868800803	0.808344967	0.93377811
			Simple mode	0.154090401	0.81715626	0.620108128	1.076819225
			Weighted mode	0.127668933	0.829193966	0.652710689	1.053395701
prot-a-2400	Pregnancy-specific beta-1-glycoprotein 11	20	MR Egger	0.619351044	1.071932022	0.818832584	1.403263966
			Weighted median	0.011313969	1.245484169	1.050902529	1.476093902
			Inverse variance weighted	0.000281446	1.261021025	1.112666064	1.429156579
			Simple mode	0.352150823	1.146872164	0.865403224	1.519887752
			Weighted mode	0.119983579	1.237217813	0.9575263	1.598606655
prot-a-2408	Pregnancy-specific beta-1-glycoprotein 9	33	MR Egger	0.527536053	0.880872417	0.59694879	1.299837154
			Weighted median	0.012263072	0.872188274	0.783667809	0.970707711
			Inverse variance weighted	0.0000103725911	0.837499446	0.774015506	0.906190271
			Simple mode	0.004113414	0.719683357	0.584185695	0.886608725
			Weighted mode	0.266778577	0.908061182	0.768210447	1.073371382

SNPs, single nucleotide polymorphisms; OR, Odds ratio; 95%CI, 95% Confidence Interval.

Analysis of other maternal infections revealed distinct causal profiles, as detailed in Table 3. Statistically significant causal relationships (*P* < 0.05) were identified between multiple factors and other maternal infections, including spontaneous miscarriage or termination, BMI, CRP, LDL cholesterol, TC, TG, IL-13 levels, IL-2Ra levels, NT-proBNP, cholesterol-lowering medications, blood pressure medication, and vitamin D levels. These findings indicated their potential causal roles in the development of other maternal infections. Using the IVW method, factors with OR > 1 were classified as potential risk factors of other maternal infections, such as spontaneous miscarriage or termination, CRP, IL-13 levels, NT-proBNP, blood pressure medication, and vitamin D levels. Conversely, the IVW method found that BMI, LDL cholesterol, total cholesterol, triglycerides, IL-2Ra levels, and cholesterol-lowering medication (all with OR < 1) emerged as potential protective factors, suggesting a negative association with infection risk.

**Table 3:** Results of the relationship between the variable factors and the risk of other maternal infection.

id.exposure	Exposure	SNPs	Method	P value	OR	Lower 95%CI	Upper 95%CI
ebi-a-GCST90095034	Body mass index	51	MR Egger	0.891030985	0.915329861	0.259856995	3.224191654
			Weighted median	0.512392841	0.864028786	0.557996018	1.337905147
			Inverse variance weighted	0.030158307	0.709525287	0.520276669	0.967612356
			Simple mode	0.936353471	1.034170713	0.455224881	2.34940819
			Weighted mode	0.793326083	0.925320026	0.519354171	1.648618993
ieu-a-94	Body mass index	67	MR Egger	0.89395571	1.08006344	0.349579277	3.336974216
			Weighted median	0.52106033	0.875543194	0.583410457	1.313956367
			Inverse variance weighted	0.015545607	0.717571317	0.548402385	0.938924792
			Simple mode	0.004967441	0.243077147	0.093661257	0.630853154
			Weighted mode	0.906160939	0.961885166	0.505350699	1.830853454
ieu-b-4815	Body mass index	59	MR Egger	0.044589208	0.826465969	0.689019628	0.991330248
			Weighted median	0.258357482	0.95324337	0.877286591	1.0357766
			Inverse variance weighted	0.000997478	0.908197398	0.857582139	0.961800016
			Simple mode	0.577389309	1.056845939	0.871014527	1.282324581
			Weighted mode	0.979885109	0.997981666	0.853504959	1.166914609
ukb-b-12621	Ever had stillbirth, spontaneous miscarriage or termination	215	MR Egger	0.01644041	16.68877445	1.704534707	163.3966099
			Weighted median	0.544530301	1.651096394	0.326136004	8.358841922
			Inverse variance weighted	0.023127631	3.359175266	1.180669579	9.557338195
			Simple mode	0.413928906	0.132250775	0.00104164	16.79108411
			Weighted mode	0.51919405	0.226339319	0.002488983	20.5824982
ukb-a-448	Medication for cholesterol, blood pressure, diabetes, or take exogenous hormones: Cholesterol lowering medication	10	MR Egger	0.390635006	0.063640621	0.000166036	24.3931167
			Weighted median	0.017017921	0.047486031	0.003886562	0.580184555
			Inverse variance weighted	0.008457575	0.073335426	0.010488928	0.5127392
			Simple mode	0.12270679	0.02929597	0.000504179	1.702280609
			Weighted mode	0.124975226	0.043023747	0.001123678	1.647307126
ukb-b-17805	Medication for cholesterol, blood pressure, diabetes, or take exogenous hormones: Cholesterol lowering medication	28	MR Egger	0.03210947	0.011029112	0.000223011	0.545449518
			Weighted median	0.017312032	0.074679913	0.008815745	0.632628247
			Inverse variance weighted	0.002837364	0.101992556	0.022779337	0.456663046
			Simple mode	0.070572233	0.02811325	0.000682422	1.158161477
			Weighted mode	0.11964277	0.069290551	0.002672177	1.796729965
ukb-b-18009	Medication for cholesterol, blood pressure, diabetes, or take exogenous hormones: Blood pressure medication	20	MR Egger	0.882245411	0.482545482	0.000035898	6486.521616
			Weighted median	0.060928905	10.40175695	0.898111116	120.4712266
			Inverse variance weighted	0.009595695	10.71577348	1.780601582	64.4882058
			Simple mode	0.101129173	37.18033426	0.608107904	2273.243359
			Weighted mode	0.111957585	29.21290536	0.552304716	1545.150377
ebi-a-GCST005067	C-reactive protein levels	17	MR Egger	0.796189563	1.124476056	0.468952759	2.696319355
			Weighted median	0.010931496	1.357544545	1.072779351	1.717899575
			Inverse variance weighted	0.00502943	1.301375771	1.082599023	1.564363964
			Simple mode	0.131685432	1.431396876	0.919601533	2.228026971
			Weighted mode	0.111779545	1.431396876	0.942682966	2.173474107
ebi-a-GCST90018730	C-reactive protein	53	MR Egger	0.375498015	1.316761217	0.720300244	2.407135243
			Weighted median	0.018942073	1.569382231	1.077087337	2.28668605
			Inverse variance weighted	0.000706729	1.571755344	1.209870209	2.041884197
			Simple mode	0.305283359	1.362239862	0.758765945	2.445678349
			Weighted mode	0.082079074	1.515821229	0.957086853	2.400737184
ebi-a-GCST000759	LDL cholesterol	166	MR Egger	0.437452869	0.905822499	0.706120345	1.162003624
			Weighted median	0.00448905	0.77493768	0.649961703	0.923944294
			Inverse variance weighted	0.0000026794	0.766586593	0.686055225	0.856570992
			Simple mode	0.095937475	0.706177982	0.469961911	1.061122892
			Weighted mode	0.263659628	0.846616324	0.63287721	1.132540704
ebi-a-GCST005068	LDL cholesterol	10	MR Egger	0.093097307	0.631242684	0.393311362	1.013109118
			Weighted median	0.017423333	0.732439333	0.566626777	0.946773781
			Inverse variance weighted	0.003830062	0.733211185	0.594135036	0.904842517
			Simple mode	0.111775718	0.703794215	0.476235031	1.04008791
			Weighted mode	0.088567329	0.716807367	0.509285883	1.008888756
ebi-a-GCST90018741	LDL cholesterol	85	MR Egger	0.321153962	0.88310166	0.691807659	1.127290991
			Weighted median	0.03014286	0.755764737	0.586747845	0.973468148
			Inverse variance weighted	0.000857192	0.770547508	0.66106398	0.898163384
			Simple mode	0.000345903	0.443534541	0.289361235	0.679852258
			Weighted mode	0.022201985	0.764203947	0.609491006	0.958189156
ieu-a-781	LDL cholesterol	133	MR Egger	0.002382081	0.726461582	0.593492795	0.889221292
			Weighted median	0.00842772	0.784753714	0.655263274	0.939833524
			Inverse variance weighted	0.00001852412	0.78594139	0.703893288	0.877553287
			Simple mode	0.298023451	0.825466716	0.576012649	1.182951974
			Weighted mode	0.045221485	0.797397264	0.640255824	0.993106776
ieu-b-4845	LDL cholesterol	66	MR Egger	0.029327673	0.753041996	0.586823025	0.966342873
			Weighted median	0.000611801	0.723599391	0.601347347	0.870704894
			Inverse variance weighted	0.0000131739	0.75144733	0.660805923	0.854521835
			Simple mode	0.293977546	0.804099418	0.53689448	1.204288548
			Weighted mode	0.053483286	0.735456442	0.541467394	0.99894506
ebi-a-GCST90018754	Total cholesterol levels	139	MR Egger	0.412450582	0.832239099	0.537165041	1.289402445
			Weighted median	0.001077177	0.600022601	0.441759261	0.814984888
			Inverse variance weighted	0.002170221	0.73657837	0.605805346	0.895580896
			Simple mode	0.00502523	0.418196564	0.229669718	0.761477689
			Weighted mode	0.004359167	0.553293067	0.370814469	0.825569777
ieu-a-782	Total cholesterol	135	MR Egger	0.179557034	0.818838391	0.612494903	1.094696965
			Weighted median	0.202771291	0.887220701	0.738008358	1.066601161
			Inverse variance weighted	0.014166294	0.85500402	0.754413863	0.969006416
			Simple mode	0.671864424	1.102694593	0.702169846	1.731682686
			Weighted mode	0.637025394	0.914112915	0.630045562	1.326257133
ieu-b-4849	Triglycerides	89	MR Egger	0.005978125	0.62604703	0.452010731	0.867091989
			Weighted median	0.019493486	0.828472757	0.707471658	0.970169054
			Inverse variance weighted	0.001251977	0.826188393	0.735717911	0.927783939
			Simple mode	0.208611554	1.318322326	0.859619297	2.021794719
			Weighted mode	0.631005081	1.092184966	0.763075994	1.563236178
ebi-a-GCST90012082	N-terminal prohormone brain natriuretic peptide levels	6	MR Egger	0.788167808	1.491910137	0.097353956	22.86292151
			Weighted median	0.007506519	1.420002782	1.098101689	1.836267008
			Inverse variance weighted	0.001624768	1.410324814	1.138810379	1.746573545
			Simple mode	0.08443591	1.424625963	1.031492598	1.967594473
			Weighted mode	0.095355076	1.42523893	1.016074909	1.99916954
ebi-a-GCST005367	Vitamin D levels	35	MR Egger	0.683150889	1.264172287	0.414234557	3.858035363
			Weighted median	0.043827867	1.910543097	1.018060885	3.585419085
			Inverse variance weighted	0.0000760096	2.394771114	1.553775729	3.690962977
			Simple mode	0.270035856	1.9766279	0.600670873	6.50449028
			Weighted mode	0.191898937	1.765414446	0.764661666	4.075904815
ebi-a-GCST004443	Interleukin-13 levels	139	MR Egger	0.119615633	1.121765321	0.971519749	1.295246378
			Weighted median	0.002939323	1.190584222	1.061282521	1.335639438
			Inverse variance weighted	0.00000046607	1.184044101	1.108752649	1.264448328
			Simple mode	0.094575574	1.248341314	0.964196587	1.61622231
			Weighted mode	0.008498061	1.229915264	1.056567366	1.431703841
ebi-a-GCST004454	Interleukin-2 receptor antagonist levels	107	MR Egger	0.010658579	0.839400881	0.735628793	0.957811663
			Weighted median	0.00059236	0.819946681	0.732138476	0.918286064
			Inverse variance weighted	0.001203954	0.889222525	0.82821925	0.954719054
			Simple mode	0.316223361	0.882840218	0.692706704	1.125161408
			Weighted mode	0.009743201	0.824355756	0.713933087	0.951857289

SNPs, single nucleotide polymorphisms; OR, Odds ratio; 95%CI, 95% Confidence Interval.

All instrumental variables showed F-statistics greater than 10 (S7 Table), indicating a low risk of weak instrument bias. The results of harmonization analyses in the Mendelian randomization analysis were available in S8 Table. The results of causal effects using five Mendelian randomization methods were summarized in S9 Table. The validity of MR estimates was systematically evaluated through pleiotropy (S10 Table), heterogeneity (S11 Table), and directionality tests (S12 Table). As summarized in Table 4, all Cochran’s Q statistics returned P-values greater than 0.05, indicating negligible heterogeneity among instrumental variables and supporting the use of fixed-effects IVW models. Similarly, pleiotropy assessment via MR-Egger and MR-PRESSO showed no evidence of horizontal pleiotropy (all P > 0.05, Table 5), confirming the robustness of causal estimates. The detailed results of MR-PRESSO global test were available in S13 Table. Finally, directionality testing verified the assumed causal direction, with outcome variance consistently lower than exposure variance across all analyses (Table 6).The results of the sensitivity analysis showed that no significantly deviating SNPs were found in this study (S5 Fig for maternal sepsis and S6 Fig for other maternal infections), indicating that the analysis results are stable and reliable.

**Table 4:** MR heterogeneity analyses of associations between variable factors and MSMI.

id.exposure	Outcome	Exposure	Method	Q-statistic	Q-df	Q P-value
ebi-a-GCST000755	Maternal sepsis	HDL cholesterol	MR Egger	132.742861	128	0.36903889
			Inverse variance weighted	133.274913	129	0.38030986
ebi-a-GCST005068	Maternal sepsis	LDL cholesterol	MR Egger	1.92050296	8	0.98335275
			Inverse variance weighted	2.43014945	9	0.98270334
ebi-a-GCST90101747	Maternal sepsis	Total cholesterol levels	MR Egger	3.42634417	6	0.7537421
			Inverse variance weighted	3.47594974	7	0.83776626
ebi-a-GCST000758	Maternal sepsis	Triglycerides	MR Egger	78.9947419	141	0.99999448
			Inverse variance weighted	80.4237597	142	0.99999272
ieu-a-783	Maternal sepsis	Triglycerides	MR Egger	107.083447	163	0.99977325
			Inverse variance weighted	107.752613	164	0.99978168
ieu-b-4850	Maternal sepsis	Triglycerides	MR Egger	94.6804383	162	0.99999455
			Inverse variance weighted	94.9594172	163	0.99999544
ukb-a-224	Maternal sepsis	Smoking status: Previous	MR Egger	132.46237	149	0.83069857
			Inverse variance weighted	135.00376	150	0.80445374
ebi-a-GCST004431	Maternal sepsis	RANTES levels	MR Egger	93.9900647	119	0.95609204
			Inverse variance weighted	94.9319698	120	0.95572199
ebi-a-GCST004443	Maternal sepsis	Interleukin-13 levels	MR Egger	127.041642	137	0.71779248
			Inverse variance weighted	127.101413	138	0.73686716
ebi-a-GCST004444	Maternal sepsis	Interleukin-10 levels	MR Egger	2.94596968	5	0.70831557
			Inverse variance weighted	2.967531	6	0.81291087
ebi-a-GCST004447	Maternal sepsis	Interleukin-1-receptor antagonist levels	MR Egger	122.007611	103	0.09741256
			Inverse variance weighted	124.509633	104	0.08323955
ebi-a-GCST004456	Maternal sepsis	Interferon gamma levels	MR Egger	110.356099	119	0.70228183
			Inverse variance weighted	113.453303	120	0.65072466
ieu-a-27	Maternal sepsis	Birth weight	MR Egger	12.8567604	11	0.30279053
			Inverse variance weighted	18.2361736	12	0.10870539
ukb-a-448	Maternal sepsis	Medication for cholesterol blood pressure diabetes or take exogenous hormones: Cholesterol lowering medication	MR Egger	3.50251837	8	0.89899411
			Inverse variance weighted	3.88877634	9	0.91857919
ukb-b-18009	Maternal sepsis	Medication for cholesterol,	MR Egger	4.40320971	18	0.99952805
		blood pressure, diabetes, or take exogenous hormones: Blood pressure medication				
			Inverse variance weighted	4.40747778	19	0.99977691
prot-a-2400	Maternal sepsis	Pregnancy-specific beta-1-glycoprotein 11	MR Egger	5.61340087	18	0.9975288
			Inverse variance weighted	7.39602758	19	0.9917841
prot-a-2408	Maternal sepsis	Pregnancy-specific beta-1-glycoprotein 9	MR Egger	17.3492977	31	0.97700756
			Inverse variance weighted	17.4167633	32	0.98310086
ukb-a-355	Maternal sepsis	Number of pregnancy terminations	MR Egger	25.8012946	26	0.47406812
			Inverse variance weighted	25.8630926	27	0.52620851
ukb-b-12621	Other maternal infections	Ever had stillbirth, spontaneous miscarriage or termination	MR Egger	175.286843	213	0.97225125
			Inverse variance weighted	177.687787	214	0.96659426
ebi-a-GCST90095034	Other maternal infections	Body mass index	MR Egger	26.7993475	49	0.99590367
			Inverse variance weighted	26.9666765	50	0.99683189
ieu-a-94	Other maternal infections	Body mass index	MR Egger	61.3858909	65	0.60421154
			Inverse variance weighted	61.9210704	66	0.61952872
ieu-b-4815	Other maternal infections	Body mass index	MR Egger	41.2917887	57	0.94171176
			Inverse variance weighted	42.4384028	58	0.93768164

ebi-a-GCST005067	Other maternal infections	C-reactive protein levels	MR Egger	7.22842614	15	0.95099822
			Inverse variance weighted	7.34060883	16	0.96612389
ebi-a-GCST90018730	Other maternal infections	C-reactive protein	MR Egger	32.8333563	51	0.97744468
			Inverse variance weighted	33.2407969	52	0.98009883
ebi-a-GCST000759	Other maternal infections	LDL cholesterol	MR Egger	93.3720615	164	0.99999813
			Inverse variance weighted	95.524593	165	0.99999679
ebi-a-GCST005068	Other maternal infections	LDL cholesterol	MR Egger	1.43418275	8	0.99374762
			Inverse variance weighted	1.91387229	9	0.99275746
ebi-a-GCST90018741	Other maternal infections	LDL cholesterol	MR Egger	81.2222415	83	0.53470902
			Inverse variance weighted	83.1995153	84	0.50416949
ieu-a-781	Other maternal infections	LDL cholesterol	MR Egger	60.1375626	131	0.99999998
			Inverse variance weighted	60.9661753	132	0.99999998
ieu-b-4845	Other maternal infections	LDL cholesterol	MR Egger	32.6061457	64	0.99962198
			Inverse variance weighted	32.6065236	65	0.99973666
ieu-a-782	Other maternal infections	Total cholesterol	MR Egger	66.6264069	133	0.99999973
			Inverse variance weighted	66.7309577	134	0.9999998
ebi-a-GCST90018754	Other maternal infections	Total cholesterol levels	MR Egger	128.530834	137	0.68512589
			Inverse variance weighted	128.904021	138	0.69834992
ieu-b-4849	Other maternal infections	Triglycerides	MR Egger	46.7446912	87	0.99987418
			Inverse variance weighted	49.9355313	88	0.99964051
ebi-a-GCST004443	Other maternal infections	Interleukin-13 levels	MR Egger	118.312122	137	0.87370069
			Inverse variance weighted	118.997614	138	0.87704775
ebi-a-GCST004454	Other maternal infections	Interleukin-2 receptor antagonist levels	MR Egger	94.79927	105	0.75228244
			Inverse variance weighted	95.832303	106	0.75041729
ebi-a-GCST90012082	Other maternal infections	N-terminal prohormone brain natriuretic peptide levels	MR Egger	0.02316797	4	0.99993342
			Inverse variance weighted	0.02480886	5	0.99999489
ukb-a-448	Other maternal infections	Medication for cholesterol blood pressure diabetes or take exogenous hormones: Cholesterol lowering medication	MR Egger	3.90319485	8	0.86575018
			Inverse variance weighted	3.90563851	9	0.91751258
ukb-b-17805	Other maternal infections	Medication for cholesterol, blood pressure, diabetes, or take exogenous hormones: Cholesterol lowering medication	MR Egger	18.1140805	26	0.87158352
			Inverse variance weighted	19.5794325	27	0.84793373
ukb-b-18009	Other maternal infections	Medication for cholesterol, blood pressure, diabetes, or take exogenous hormones: Blood pressure medication	MR Egger	11.2938293	18	0.88147437
			Inverse variance weighted	11.7175694	19	0.89734009
ebi-a-GCST005367	Other maternal infections	Vitamin D levels	MR Egger	15.8644933	33	0.99485326
			Inverse variance weighted	17.346877	34	0.99203558

**MSMI, maternal sepsis and maternal other infections; Q,Cochran’s Q; Q_df,degrees of freedom for Cochran’s Q; Q_pval, p-value of statistical significance for Cochran’s Q**.

**Table 5:** MR pleiotropy test of associations between variable factors and MSMI.

id.exposure	Outcome	Exposure	Egger intercept	Egger P-value	MR-PRESSO RSSobs	MR-PRESSO global P-value
ebi-a-GCST000755	Maternal sepsis	HDL cholesterol	0.005672223	0.475129483	135.5979561	0.396
ebi-a-GCST000758	Maternal sepsis	Triglycerides	0.009410656	0.233931454	81.63661094	1
ebi-a-GCST005068	Maternal sepsis	LDL cholesterol	0.038542093	0.495589402	2.898157208	0.984
ebi-a-GCST90101747	Maternal sepsis	Total cholesterol levels	-0.008498967	0.831139435	4.173755419	0.852
ieu-a-783	Maternal sepsis	Triglycerides	0.006300554	0.414536915	109.5645232	1
ieu-b-4850	Maternal sepsis	Triglycerides	-0.003925279	0.598094071	96.24572275	1
ukb-a-224	Maternal sepsis	Smoking status: Previous	-0.025849254	0.113016177	137.0172496	0.786
ebi-a-GCST004431	Maternal sepsis	RANTES levels	0.009938388	0.333756893	96.53206568	0.971
ebi-a-GCST004443	Maternal sepsis	Interleukin-13 levels	0.001997058	0.807223541	129.4788713	0.734
ebi-a-GCST004444	Maternal sepsis	Interleukin-10 levels	-0.003905133	0.888997096	3.894627352	0.833
ebi-a-GCST004447	Maternal sepsis	Interleukin-1-receptor antagonist levels	0.016208237	0.149166153	126.8630402	0.106
ebi-a-GCST004456	Maternal sepsis	Interferon gamma levels	-0.014643297	0.080996492	115.2668184	0.681
ieu-a-27	Maternal sepsis	Birth weight	-0.136888781	0.055090675	21.09228962	0.148
ukb-a-448	Maternal sepsis	Medication for cholesterol blood pressure diabetes or take exogenous hormones: Cholesterol lowering medication	-0.024727881	0.551567389	4.801907271	0.926
ukb-b-18009	Maternal sepsis	Medication for cholesterol, blood pressure, diabetes, or take exogenous hormones: Blood pressure medication	0.00287889	0.948631014	4.92575117	0.999
prot-a-2400	Maternal sepsis	Pregnancy-specific beta-1-glycoprotein 11	0.041587445	0.198468248	8.238128917	0.993
prot-a-2408	Maternal sepsis	Pregnancy-specific beta-1-glycoprotein 9	-0.008816342	0.796780532	18.53511745	0.982
ukb-a-355	Maternal sepsis	Number of pregnancy terminations	0.005594817	0.805629574	27.5872085	0.556
ukb-b-12621	Other maternal infections	Ever had stillbirth, spontaneous miscarriage or termination	-0.015614573	0.122746547	179.5733172	0.961
ebi-a-GCST90095034	Other maternal infections	Body mass index	-0.014233343	0.684279045	28.00369296	0.996
ieu-a-94	Other maternal infections	Body mass index	-0.021032711	0.467066726	63.53281603	0.65
ieu-b-4815	Other maternal infections	Body mass index	0.024722583	0.288772319	43.8810214	0.936
ebi-a-GCST005067	Other maternal infections	C-reactive protein levels	0.019603322	0.742310664	8.452549367	0.965
ebi-a-GCST90018730	Other maternal infections	C-reactive protein	0.011413996	0.526127458	34.24911822	0.985
ebi-a-GCST000759	Other maternal infections	LDL cholesterol	-0.013729077	0.144250098	96.71864105	1
ebi-a-GCST005068	Other maternal infections	LDL cholesterol	0.057373017	0.508165114	2.204985768	0.999
ebi-a-GCST90018741	Other maternal infections	LDL cholesterol	-0.015208192	0.163410733	84.91942696	0.526
ebi-a-GCST90018754	Other maternal infections	Total cholesterol levels	-0.006943867	0.542284304	130.483698	0.71
ieu-a-781	Other maternal infections	LDL cholesterol	0.008484727	0.364345832	61.83519629	1
ieu-a-782	Other maternal infections	Total cholesterol	0.003766704	0.746943237	67.788827	1
ieu-b-4845	Other maternal infections	LDL cholesterol	-0.000289938	0.984549717	33.56187092	1
ieu-b-4849	Other maternal infections	Triglycerides	0.031192006	0.077535697	51.07842566	1
ebi-a-GCST004443	Other maternal infections	Interleukin-13 levels	0.010376436	0.409141706	120.6098671	0.894
ebi-a-GCST004454	Other maternal infections	Interleukin-2 receptor antagonist levels	0.012829042	0.311783622	97.39915124	0.763
ebi-a-GCST90012082	Other maternal infections	N-terminal prohormone brain natriuretic peptide levels	-0.010254851	0.969629419	0.035985447	1
ukb-a-448	Other maternal infections	Medication for cholesterol blood pressure diabetes or take exogenous hormones: Cholesterol lowering medication	0.003016256	0.96178583	4.878109826	0.922
ukb-b-17805	Other maternal infections	Medication for cholesterol, blood pressure, diabetes, or take exogenous hormones: Cholesterol lowering medication	0.036947558	0.236970417	20.83373081	0.866
ukb-b-18009	Other maternal infections	Medication for cholesterol, blood pressure, diabetes, or take exogenous hormones: Blood pressure medication	0.043963533	0.523300433	12.97198381	0.915
ebi-a-GCST005367	Other maternal infections	Vitamin D levels	0.026331916	0.232036799	18.41384171	0.988

**MR-PRESSO, MR pleiotropy residual sum and outlier; MSMI, maternal sepsis and maternal other infections; RSSobs, residual sum of square observed;MR-PRESSO global P-value, p-value of statistical significance for MR-PRESSO global test**.

**Table 6:** MR Steiger directionality test.

id.exposure	Outcome	Exposure	snp_r2.exposure	snp_r2.outcome	correct_causal_direction	Steiger_pval
ebi-a-GCST000755	Maternal sepsis	HDL cholesterol	0.172766974	0.00116295	TRUE	0
ebi-a-GCST000758	Maternal sepsis	Triglycerides	0.260936468	0.000789068	TRUE	0
ebi-a-GCST005068	Maternal sepsis	LDL cholesterol	0.13670689	8.77472251754687e-05	TRUE	4.48934246922421e-289
ebi-a-GCST90101747	Maternal sepsis	Total cholesterol levels	0.032949807	7.60439910408359e-05	TRUE	4.82117443703251e-138
ieu-a-783	Maternal sepsis	Triglycerides	0.375332955	0.001008277	TRUE	0
ieu-b-4850	Maternal sepsis	Triglycerides	0.417524705	0.000844268	TRUE	0
ukb-a-224	Maternal sepsis	Smoking status: Previous	0.018538819	0.001157995	TRUE	1.14026032633963e-207
ebi-a-GCST004431	Maternal sepsis	RANTES levels	0.672725925	0.000962158	TRUE	0
ebi-a-GCST004443	Maternal sepsis	Interleukin-13 levels	0.872902105	0.001081431	TRUE	0
ebi-a-GCST004444	Maternal sepsis	Interleukin-10 levels	0.090442191	0.000100461	TRUE	1.19379412532228e-143
ebi-a-GCST004447	Maternal sepsis	Interleukin-1-receptor antagonist levels	0.526073036	0.001068639	TRUE	0
ebi-a-GCST004456	Maternal sepsis	Interferon gamma levels	0.294069903	0.001054506	TRUE	0
ieu-a-27	Maternal sepsis	Birth weight	0.018501074	0.000224668	TRUE	5.71534446473855e-73
ukb-a-448	Maternal sepsis	Medication for cholesterol blood pressure diabetes or take exogenous hormones: Cholesterol lowering medication	0.010561014	9.73078824485904e-05	TRUE	2.93924236218571e-139
ukb-b-18009	Maternal sepsis	Medication for cholesterol, blood pressure, diabetes, or take exogenous hormones: Blood pressure medication	0.010065199	7.14657434896656e-05	TRUE	4.24151069540903e-153
prot-a-2400	Maternal sepsis	Pregnancy-specific beta-1-glycoprotein 11	0.13747845	0.000169513	TRUE	6.93527095697716e-101
prot-a-2408	Maternal sepsis	Pregnancy-specific beta-1-glycoprotein 9	0.285192338	0.000303507	TRUE	1.50002556130939e-235
ukb-a-355	Maternal sepsis	Number of pregnancy terminations	0.011390177	0.000256786	TRUE	6.17487302983546e-71
ukb-b-12621	Other maternal infections	Ever had stillbirth, spontaneous miscarriage or termination	0.015735548	0.00152237	TRUE	2.81990950863449e-135
ebi-a-GCST90095034	Other maternal infections	Body mass index	0.039895709	0.000263659	TRUE	1.53542408021117e-290
ieu-a-94	Other maternal infections	Body mass index	0.052128823	0.000564291	TRUE	0
ieu-b-4815	Other maternal infections	Body mass index	0.072172921	0.00044353	TRUE	0
ebi-a-GCST005067	Other maternal infections	C-reactive protein levels	0.078534254	0.000126633	TRUE	3.90595461583497e-149
ebi-a-GCST90018730	Other maternal infections	C-reactive protein	0.070119209	0.000372273	TRUE	0
ebi-a-GCST000759	Other maternal infections	LDL cholesterol	0.240555508	0.000978787	TRUE	0
ebi-a-GCST005068	Other maternal infections	LDL cholesterol	0.13670689	8.5562584656784e-05	TRUE	5.19993733888866e-289
ebi-a-GCST90018741	Other maternal infections	LDL cholesterol	0.203102753	0.000785247	TRUE	0
ebi-a-GCST90018754	Other maternal infections	Total cholesterol levels	0.134441683	0.001151513	TRUE	0
ieu-a-781	Other maternal infections	LDL cholesterol	0.325527243	0.000660266	TRUE	0
ieu-a-782	Other maternal infections	Total cholesterol	0.250530156	0.000605703	TRUE	0
ieu-b-4845	Other maternal infections	LDL cholesterol	0.317324796	0.000429552	TRUE	0
ieu-b-4849	Other maternal infections	Triglycerides	0.288618339	0.000502453	TRUE	0
ebi-a-GCST004443	Other maternal infections	Interleukin-13 levels	0.872902105	0.00120224	TRUE	0
ebi-a-GCST004454	Other maternal infections	Interleukin-2 receptor antagonist levels	0.722869916	0.000885187	TRUE	0
ebi-a-GCST90012082	Other maternal infections	N-terminal prohormone brain natriuretic peptide levels	0.035021417	8.28959349926104e-05	TRUE	3.48384787578417e-132
ukb-a-448	Other maternal infections	Medication for cholesterol blood pressure diabetes or take exogenous hormones: Cholesterol lowering medication	0.010561014	9.02500143124042e-05	TRUE	2.02207235991357e-139
ukb-b-17805	Other maternal infections	Medication for cholesterol, blood pressure, diabetes, or take exogenous hormones: Cholesterol lowering medication	0.018753757	0.000237196	TRUE	3.13531143849049e-266
ukb-b-18009	Other maternal infections	Medication for cholesterol, blood pressure, diabetes, or take exogenous hormones: Blood pressure medication	0.010065199	0.000153414	TRUE	1.85145527787838e-139
ebi-a-GCST005367	Other maternal infections	Vitamin D levels	0.130750127	0.000274771	TRUE	0

**snp, single nucleotide polymorphisms; Steiger_pval, p value of Steiger directionality test**.

LASSO regression retained key inflammatory biomarkers, including RANTES, IL-10, CRP, IL-13, and NT-proBNP, for subsequent multivariable MR analysis (MVMR). MVMR analysis was employed to evaluate the independent causal roles of specific inflammatory biomarkers in MSMI. For maternal sepsis, genetically predicted elevations in both RANTES and IL-10 levels demonstrated significant positive associations with disease risk (Table 7). Relatively, in the analysis of other maternal infections, higher levels of CRP, IL-13, and NT-proBNP were causally linked to increased infection risk (Table 7). These results highlight distinct inflammatory pathways contributing to different forms of maternal infection and underscore the potential value of these biomarkers in risk stratification.

**Table 7:** Results of multivariate mendelian randomization (dimension reduction with LASSO) for maternal sepsis and other maternal infections.

Outcome	Exposure	SNPs	P value	OR	Lower 95%CI	Upper 95%CI	Exposure1	Exposure2
Maternal sepsis	RANTES levels	1	0	1.44802918	1.43913011	1.45698328	RANTES levels	Interleukin-10 levels
	Interleukin-10 levels	2	0	1.30709749	1.30369878	1.31050506	RANTES levels	Interleukin-10 levels
Maternal other infections	Interleukin-13 levels	1	0.00656167	1.16173727	1.04270804	1.29435416	C-reactive protein levels	Interleukin-13 levels
	C-reactive protein levels	4	0.00000023385433	1.59615066	1.33687051	1.90571707	C-reactive protein levels	Interleukin-13 levels
Maternal other infections	C-reactive protein levels	4	1.37781487119777e-10	1.60932196	1.39167948	1.86100118	C-reactive protein levels	NT-proBNP
	NT-proBNP	1	0.00000300542091	1.53884312	1.28421102	1.84396342	C-reactive protein levels	NT-proBNP

**LASSO, least absolute shrinkage and selection operator; SNPs, single nucleotide polymorphisms; OR, Odds ratio; 95%CI, 95% Confidence Interval; NT-proBNP, N-terminal prohormone brain natriuretic peptide levels**.

## Discussion

This comprehensive study firstly provides a detailed assessment of the global burden, trends, and causal determinants of MSMI from 1990 to 2021, with projections extending to 2041. Our findings reveal persistent disparities, identify modifiable risk factors, and highlight critical areas for future intervention and research.

The disproportionate burden in low- and middle-income countries, particularly in South Asia and sub-Saharan Africa, aligns with the inverse equity hypothesis proposed by Victora et al. [31], which posits that health interventions often benefit higher-resource populations first, thereby widening disparities. For example, increasing mortality was observed in Kazakhstan, a phenomenon that may be related to limited healthcare access and uneven resource distribution. In contrast, a marked decline was demonstrated in Saudi Arabia, likely reflecting improvements in maternal healthcare services. Additionally, increasing trends observed in Australia may be associated with advanced maternal age and a higher prevalence of comorbidities. The strong negative correlation between SDI and MSMI burden (Fig 6) underscores how structural determinants, including limited healthcare access, sanitation, and education-perpetuate inequitable maternal health outcomes [32]. Moreover, as a composite indicator, the SDI encompasses components such as educational attainment and fertility rate, which may also influence the burden of infections among populations with MSMI [33–34]. Notably, with respect to fertility, primiparous women tend to face greater difficulties in newborn care compared with multiparous women [35]. The observed age distribution pattern, characterized by a peak burden in the 20–24 age group and elevated risk in adolescents (15–19 years), reflects complex biological and social vulnerabilities. Younger mothers face increased physiological risks due to reproductive immaturity while simultaneously experiencing greater barriers to healthcare utilization [36]. Thus, this pattern emphasizes the need for life-course approaches to maternal health that address specific risk factors across different age groups. The overall global decline in MSMI burden since 1990 likely reflects improvements in maternal healthcare, antibiotic accessibility, and infection control practices [37]. Nevertheless, divergent trends between high- and low-SDI regions (Fig 9)-where absolute case numbers increased in low-SDI areas despite declining ASR-suggest that population growth is the main driver of the increasing absolute burden, offsetting the decline in ASR, and highlights how persistent structural barriers can undermine progress. Consequently, this finding supports the concept of the epidemiological polarization described by Bawah et al [38], where improvements in health indicators mask persistent or worsening absolute burdens in disadvantaged populations.

The MR analyses provide novel causal evidence linking specific biomarkers to MSMI risk. The identification of inflammatory markers (RANTES, IL-10, CRP) as risk factors reinforces the central role of dysregulated immunity in sepsis pathogenesis [39]. Importantly, the differential associations observed in the MR analysis may reflect distinct immunopathological mechanisms underlying MSMI. RANTES and IL-10, identified as independent risk factors for maternal sepsis, are closely involved in immune regulation and anti-inflammatory processes. Elevated IL-10 levels, in particular, may indicate an excessive compensatory anti-inflammatory response, leading to immune suppression and impaired pathogen clearance during sepsis progression [40]. Similarly, RANTES plays a key role in leukocyte recruitment and immune cell activation, and its dysregulation may contribute to maladaptive immune responses in severe infections [41]. In contrast, CRP and IL-13, which were associated with other maternal infections, are more strongly linked to systemic inflammatory responses and host defense mechanisms. CRP is a well-established marker of acute inflammation, reflecting the intensity of the innate immune response [42], while IL-13 is involved in Th2-mediated immune pathways and may influence susceptibility to certain infectious processes [43]. Although not retained as primary factors in the MVMR analysis, some protective biomarkers such as HDL and IL-1Ra may still possess potential translational relevance. HDL has been widely recognized for its anti-inflammatory and immunomodulatory properties [44], while IL-1Ra functions as a natural inhibitor of IL-1–mediated inflammatory signaling [45]. These findings suggest that modulation of inflammatory pathways may represent a potential avenue for prevention or intervention. Nevertheless, further studies are required to validate these effects in clinical settings.

Importantly, the MR findings can be partially contextualized within the observed global epidemiological patterns. Regions with higher MSMI burden, particularly low- and middle-SDI countries in South Asia and sub-Saharan Africa, are characterized by increased exposure to infection, poor nutritional status, and limited access to healthcare, all of which may contribute to chronic low-grade inflammation [46–47]. This observation is consistent with our MR findings, which identified inflammatory biomarkers, including CRP, IL-13, and RANTES, as previously reported causal risk factors for MSMI [48–50]. The concordance between epidemiological patterns and genetic evidence suggests that systemic inflammation may represent a key biological pathway linking adverse socioeconomic environments to increased maternal infection risk. Furthermore, in low- and middle-income countries where diagnostic capacity is often limited, the integration of biomarker-based approaches may improve early detection and risk stratification of maternal infections [51]. Thus, these findings suggest that maternal sepsis may be characterized by immune dysregulation and suppression, whereas other maternal infections may be driven more by heightened inflammatory activation. This distinction highlights the potential need for tailored prevention and therapeutic strategies targeting different immune pathways.

The burden estimates presented herein are largely consistent with previous Global Burden of Disease studies, which have shown declining but unevenly distributed maternal infection rates [52]. However, this study adds significant granularity by revealing how socioeconomic stratification affects both absolute and relative burden metrics-a nuance often overlooked in aggregate global analyses. The causal role of inflammatory biomarkers identified through MR extends previous observational studies that reported associations but could not establish causality [53]. Our finding that HDL cholesterol may be protective offers new insights into potential modifiable factors, complementing experimental evidence suggesting that HDL particles can neutralize bacterial toxins and modulate immune responses. Notably, the divergent projections between ARIMA and BAPC models (Figs 11-12) echo ongoing methodological debates about forecasting infectious diseases. The difference between ARIMA and BAPC results is mainly attributable to their model structures. ARIMA only considers time trends, whereas BAPC incorporates age, period, and cohort effects. Therefore, BAPC may better reflect long-term changes in population structure. Thus, ARIMA is more suitable for short-term prediction, while BAPC provides more reliable long-term estimates. Both models demonstrated good performance in diagnostic tests. The ARIMA model fit the data well based on AIC/BIC and residual tests, while the BAPC model showed stable results in posterior checks. Future studies should prioritize several key areas. First, prospective validation of the identified causal biomarkers in diverse populations is essential, particularly in high-burden regions of Africa and Asia [54]. Research exploring the mechanisms through which lipid metabolism and specific cytokines influence maternal infection susceptibility could reveal new therapeutic targets. Methodologically, developing integrated forecasting models that incorporate climate change, antimicrobial resistance patterns, and healthcare investment scenarios would enhance prediction accuracy. Implementation research examining context-specific strategies for reducing the burden in low-SDI regions is urgently needed-particularly interventions that address both clinical and social determinants of MSMI [55]. Finally, expanding genomic studies to include underrepresented populations will improve the generalizability of causal inferences and help advance precision public health approaches to maternal infection prevention. Longitudinal studies tracking how changing SDI modifies MSMI burden could also provide valuable insights for targeting interventions most effectively.

Several limitations warrant consideration when interpreting our results. First, heterogeneity in diagnostic capacity and reporting systems across countries may affect the accuracy of MSMI burden estimates, particularly in low-SDI regions. In low-resource settings, limited laboratory capacity and incomplete surveillance systems may lead to underreporting and misclassification, especially for other maternal infections. Such systematic bias may result in underestimation of the true disease burden and may weaken the observed inverse relationship between SDI and MSMI burden. Despite robust quality control, heterogeneity in diagnostic capability and reporting completeness across countries may affect burden estimates, particularly in low-resource settings [56]. This concern is especially relevant given our finding that diagnostic quality varies with socioeconomic development (Fig 9). Second, the MR analyses assume linearity of causal effects and may not capture threshold or non-linear relationships. Although MR reduces confounding, it relies on key assumptions, including the absence of unmeasured confounding of the instrument–outcome association. For complex exposures such as inflammatory cytokines and behavioral traits, genetic instruments may influence outcomes through multiple biological pathways, introducing potential bias [57]. Therefore, despite the application of sensitivity analyses to detect pleiotropy, residual bias from unknown or unmeasured pathways cannot be completely excluded. This limitation has been widely recognized in MR studies involving complex traits. Additionally, the majority of genetic instruments were derived from European-ancestry populations, which may limit the generalizability of the findings to other populations with different genetic architectures [58]. Differences in environmental exposures and gene–environment interactions across populations may further influence the validity of causal estimates. This limitation is particularly relevant for high-burden regions such as Africa and South Asia, where the applicability of the findings may be reduced. The statistical power of the MR analyses may have been insufficient to detect small but clinically meaningful effects, particularly for exposures with a limited number of genome-wide significant SNPs. Although a strict F-statistic threshold (>10) was applied to ensure strong instruments and minimize weak instrument bias, it is important to note that MR studies are inherently constrained by the variance in the exposure explained by the genetic instruments. With a limited number of SNPs, the statistical power to detect modest causal effects is reduced. Thus, null findings should not be interpreted as definitive evidence of no causal relationship, but rather as an indication that any true effect may be smaller than the study was designed to detect. Future investigations leveraging expanded GWAS datasets or employing multi-variable MR approaches to aggregate genetic signals may help overcome these power limitations. It is acknowledged that overlap between exposure and outcome GWAS samples may introduce bias in two-sample MR analyses, particularly by inflating Type I error rates and underestimating standard errors. In this study, this risk was mitigated by selecting exposure and outcome datasets from independent, large-scale consortium efforts. Specifically, the use of UK Biobank-based GWAS for exposures and data from distinct sources (e.g., the FinnGen consortium for outcomes) represents a strategy to minimize overlap, given their differing study populations, ascertainment strategies, and geographic distributions. Therefore, substantial sample overlap is unlikely in this study. Nevertheless, the possibility of partial overlap due to shared control populations or unreported study affiliations cannot be entirely excluded. Future studies utilizing strictly non-overlapping datasets or applying methods to correct for sample overlap (e.g., MRlap, pseudo-IVW) will be valuable to further validate our findings. Third, the forecasting models, though statistically validated, rely on historical trends and may not account for future healthcare breakthroughs, emerging antimicrobial resistance, or climate change impacts on infection patterns [59].

## Conclusions

In summary, this study systematically characterized the global burden and future trends of MSMI and identified key causal risk factors using MVMR. Importantly, by integrating population-level epidemiological analysis with genetic causal inference, this study provides complementary lines of evidence that strengthen the robustness of the findings. The observed discrepancy between temporal trends and absolute burden highlights the critical role of demographic changes, while the identification of inflammatory biomarkers offers potential targets for intervention. To the best of our knowledge, this is the first study to combine GBD-based trend analysis with MVMR in the context of MSMI, thereby providing a novel framework for linking macro-level disease patterns with underlying biological mechanisms. Thus, these findings may inform more precise and evidence-based public health strategies, particularly in high-burden settings.

## Supporting information

S1 Fig Spearman’s correlation analyses between MSMI Burden and SDI in 1990. (A) ASIR-SDI, (B) ASPR-SDI, (C) ASMR-SDI, (D) ASDR-SDI, (E) AS-YLD-SDI, and (F) AS-YLL-SDI. DALYs, Disability-Adjusted Life Years; YLDs, Years Lived with Disability; YLLs, Years of Life Lost. ASIR, Age-Standardized Incidence Rate; ASPR, Age-Standardized Prevalence Rate; ASMR, Age-Standardized Mortality Rate; ASDR, Age-Standardized DALYs Rate; AS-YLD, Age-Standardized YLDs Rate; AS-YLL, Age-Standardized YLLs Rate.
(PDF)

S2 Fig Spearman’s correlation analyses between MSMI Burden and SDI in 2021. (A) ASIR-SDI, (B) ASPR-SDI, (C) ASMR-SDI, (D) ASDR-SDI, (E) AS-YLD-SDI, and (F) AS-YLL-SDI. DALYs, Disability-Adjusted Life Years; YLDs, Years Lived with Disability; YLLs, Years of Life Lost. ASIR, Age-Standardized Incidence Rate; ASPR, Age-Standardized Prevalence Rate; ASMR, Age-Standardized Mortality Rate; ASDR, Age-Standardized DALYs Rate; AS-YLD, Age-Standardized YLDs Rate; AS-YLL, Age-Standardized YLLs Rate.
(PDF)

S3 Fig Decomposition results of MSMI burden across MSMI at the SDI components level. Three SDI components include population structure (aging), epidemiological changes, and population size (population). DALYs, Disability-Adjusted Life Years; YLDs, Years Lived with Disability; YLLs, Years of Life Lost.
(PDF)

S4 Fig Decomposition results of MSMI burden across sex at the SDI components level. Three SDI components include population structure (aging), epidemiological changes, and population size (population). DALYs, Disability-Adjusted Life Years; YLDs, Years Lived with Disability; YLLs, Years of Life Lost.
(PDF)

S5 Fig Leave-one-out plots for the causal association between metabolites and maternal sepsis. The consistency of results remains robust even after excluding individual genetic variants in each analysis, indicating a high level of reliability and stability in our findings.
(PDF)

S6 Fig Leave-one-out plots for the causal association between metabolites and other maternal infections. The consistency of results remains robust even after excluding individual genetic variants in each analysis, indicating a high level of reliability and stability in our findings.
(PDF)

S1 Table All original data of MSMI from the GBD database. (ZIP)

S2 Table All original data of MSMI based on age categories from the GBD database. (XLSX)

S3 Table All original data of MSMI at the country level from the GBD database. (XLSX)

S4 Table All original data of MSMI based on age categories and all ages from the GBD database. (XLSX)

S5 Table All original data of MSMI based on SDI quintiles from the GBD database. (XLSX)

S6 Table All original data of MSMI based on GBD region categories from the GBD database. (XLSX)

S7 Table Summary of F-statistics for all exposures in this study. (XLSX)

S8 Table Summary of coordinated results of instrumental variables (SNPs) for each exposure-outcome pair in this study. (XLSX)

S9 Table Summary of causal effects using five Mendelian randomization methods in this study. (XLSX)

S10 Table Summary of horizontal pleiotropy test in Mendelian randomization analysis. (XLSX)

S11 Table Summary of heterogeneity test in Mendelian randomization analysis. (XLSX)

S12 Table Summary of Steiger test for causal direction in Mendelian randomization analysis. (XLSX)

S13 Table Summary of MR-PRESSO global test in Mendelian randomization analysis. (XLSX)

S1 Checklist The filled checklist is based on the STROBE Statement-Checklist of items that should be included in reports of Mendelian randomization studies (https://www.strobe-mr.org). *Skrivankova VW, Richmond RC, Woolf BAR, et al. Strengthening the Reporting of Observational Studies in Epidemiology Using Mendelian Randomization: The STROBE-MR Statement. JAMA. 2021;326(16):1614–1621. doi:10.1001/jama.2021.18236*.
(DOCX)
