# Supplementary figures and images for: Global burden, projections, and causal factors of maternal sepsis and other maternal infections: A comprehensive epidemiological and mendelian randomization study
Source: PLoS Negl Trop Dis. 2026 May 27;20(5):e0014374. doi: 10.1371/journal.pntd.0014374 (PMC13229374; doi:10.1371/journal.pntd.0014374)

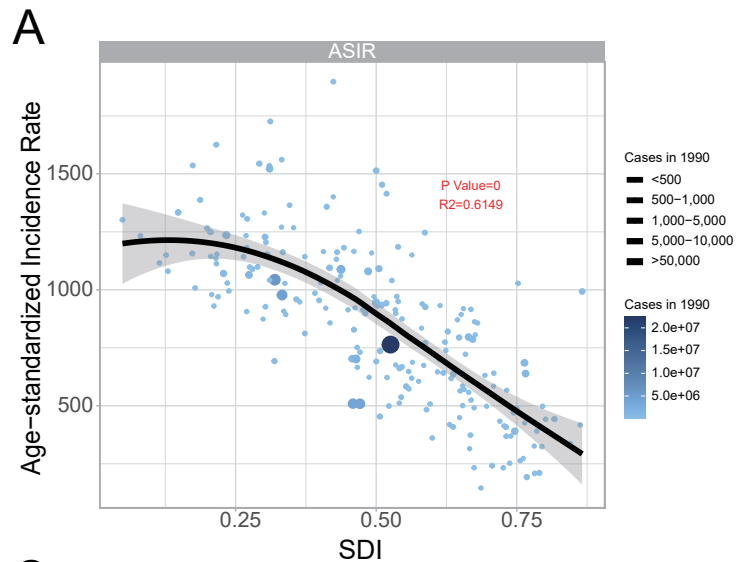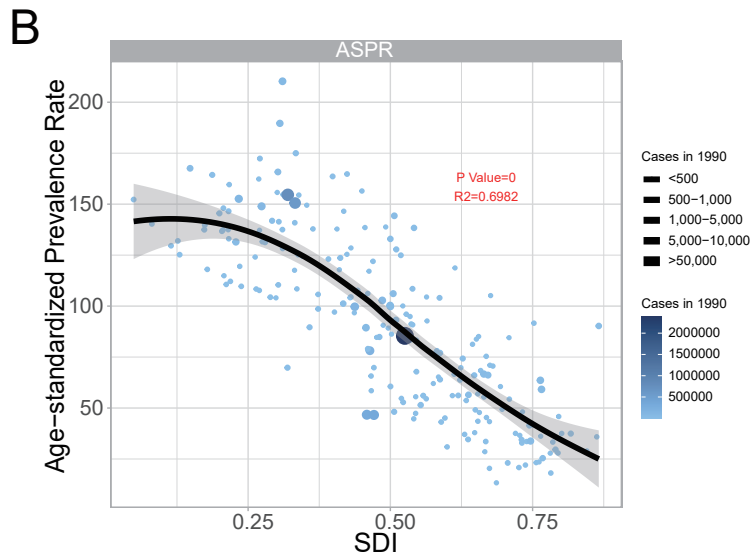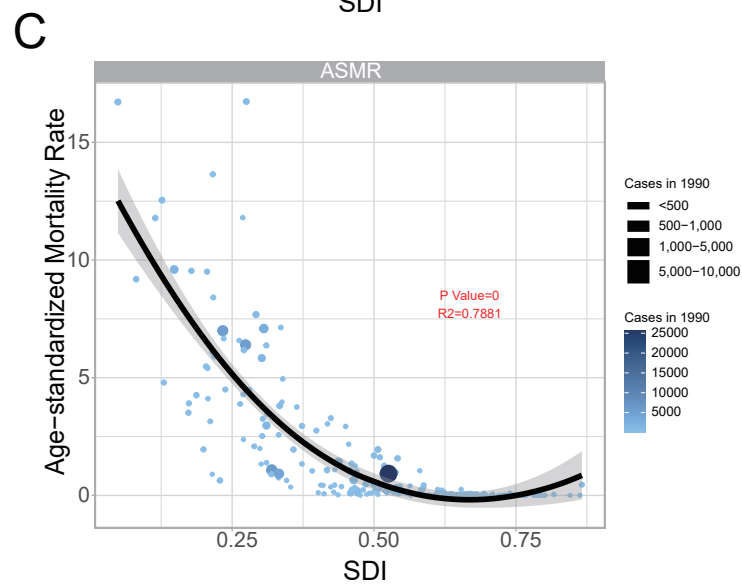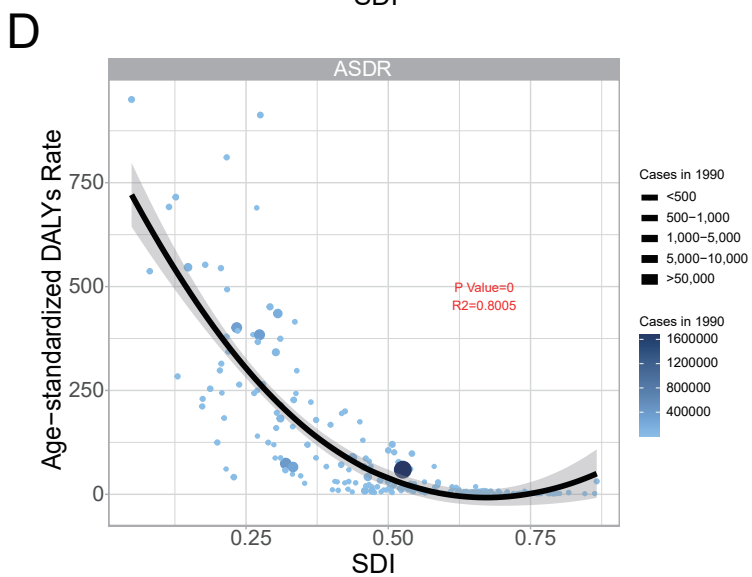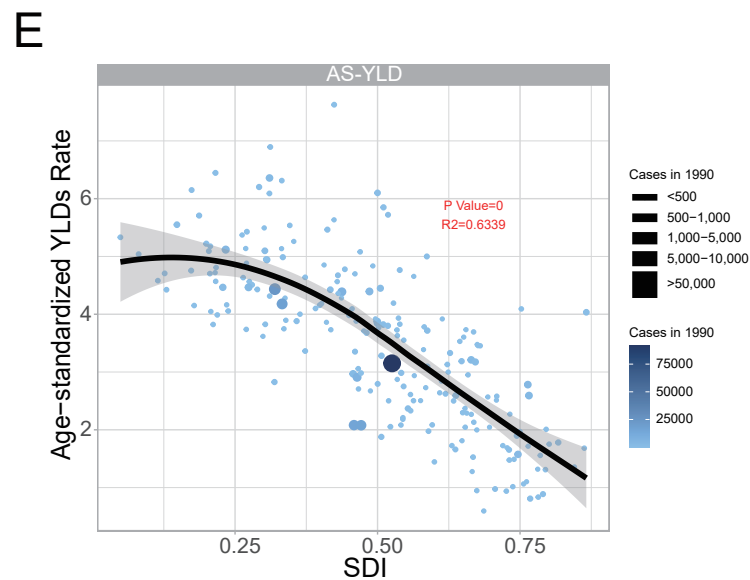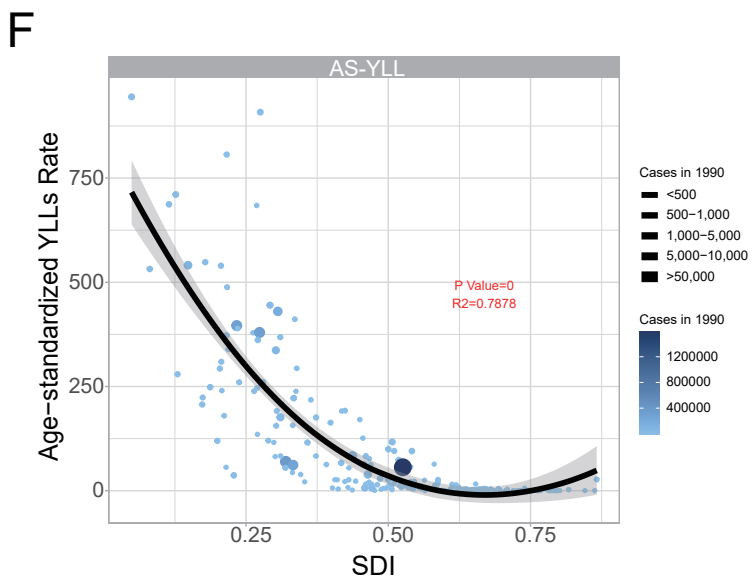

Supplement: S1 Fig — (A) ASIR-SDI, (B) ASPR-SDI, (C) ASMR-SDI, (D) ASDR-SDI, (E) AS-YLD-SDI, and (F) AS-YLL-SDI. DALYs, Disability-Adjusted Life Years; YLDs, Years Lived with Disability; YLLs, Years of Life Lost. ASIR, Age-Standardized Incidence Rate; ASPR, Age-Standardized Prevalence Rate; ASMR, Age-Standardized Mortality Rate; ASDR, Age-Standardized DALYs Rate; AS-YLD, Age-Standardized YLDs Rate; AS-YLL, Age-Standardized YLLs Rate. (PDF) [file pntd.0014374.s001.pdf]

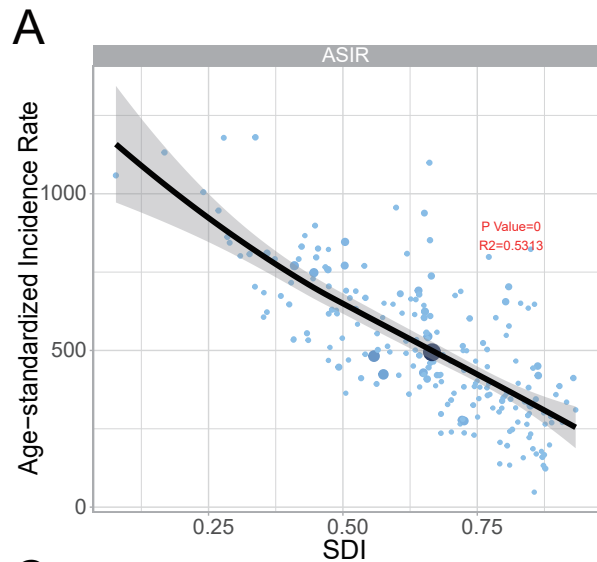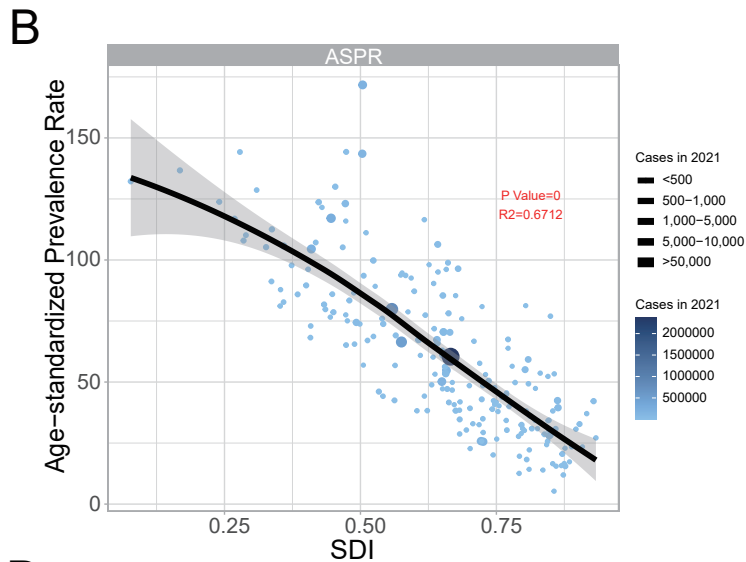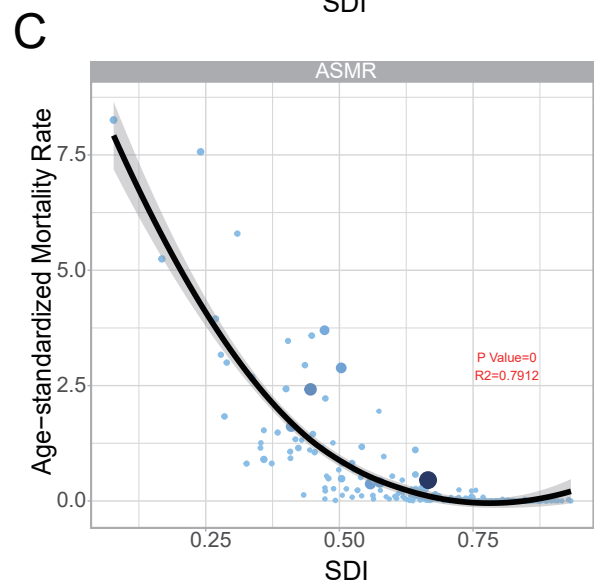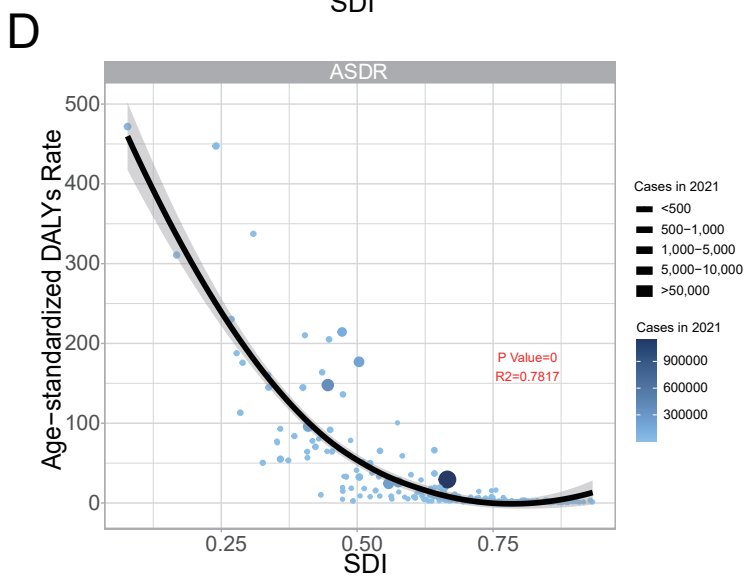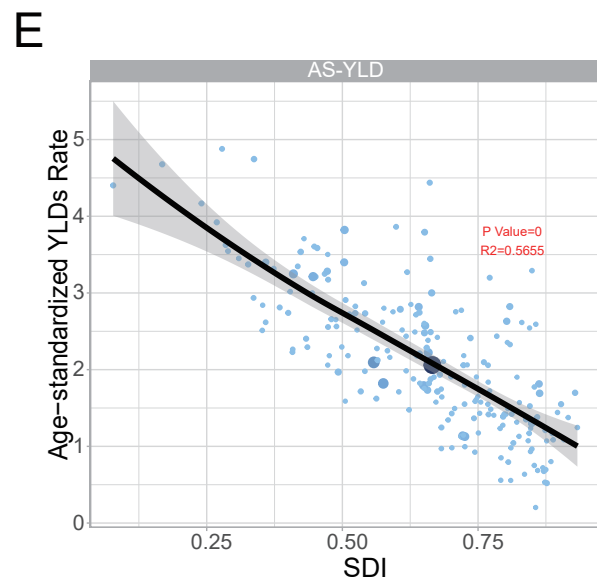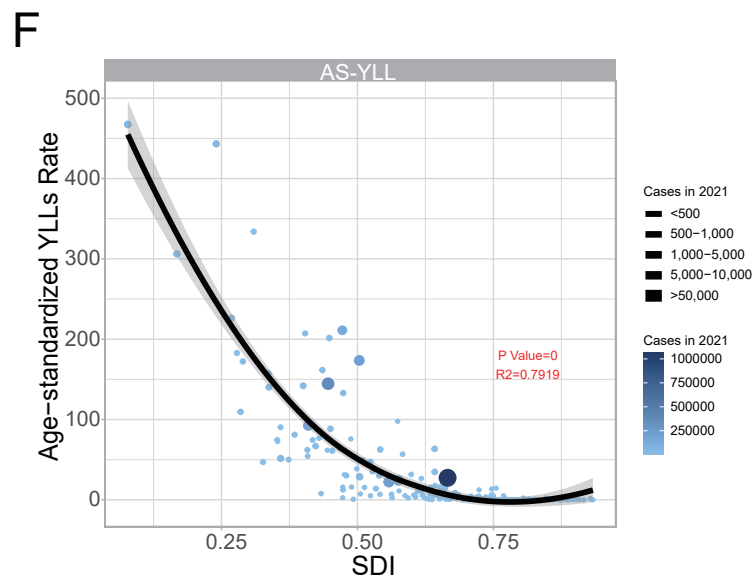

Supplement: S2 Fig — (A) ASIR-SDI, (B) ASPR-SDI, (C) ASMR-SDI, (D) ASDR-SDI, (E) AS-YLD-SDI, and (F) AS-YLL-SDI. DALYs, Disability-Adjusted Life Years; YLDs, Years Lived with Disability; YLLs, Years of Life Lost. ASIR, Age-Standardized Incidence Rate; ASPR, Age-Standardized Prevalence Rate; ASMR, Age-Standardized Mortality Rate; ASDR, Age-Standardized DALYs Rate; AS-YLD, Age-Standardized YLDs Rate; AS-YLL, Age-Standardized YLLs Rate. (PDF) [file pntd.0014374.s002.pdf]

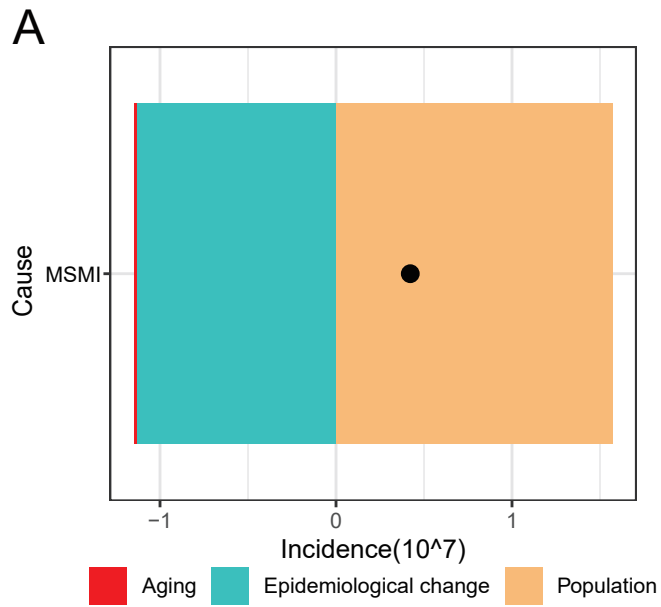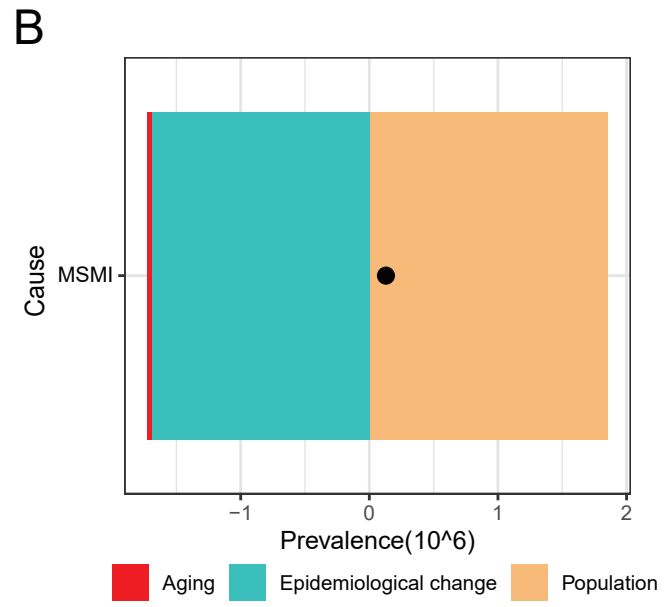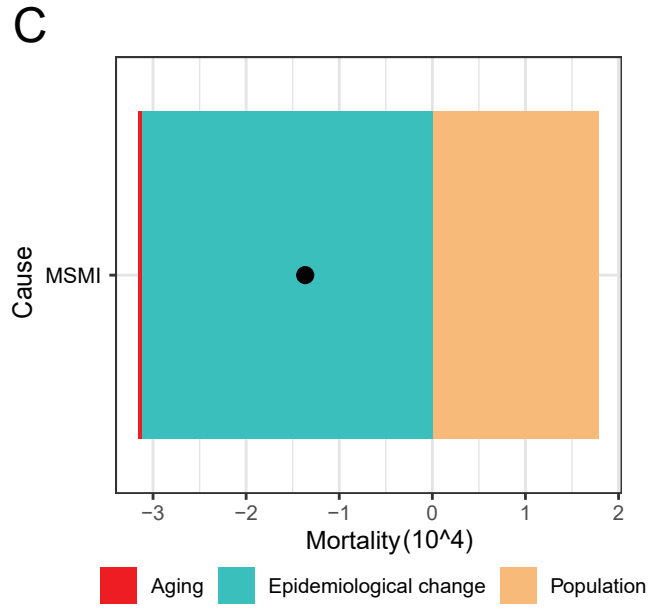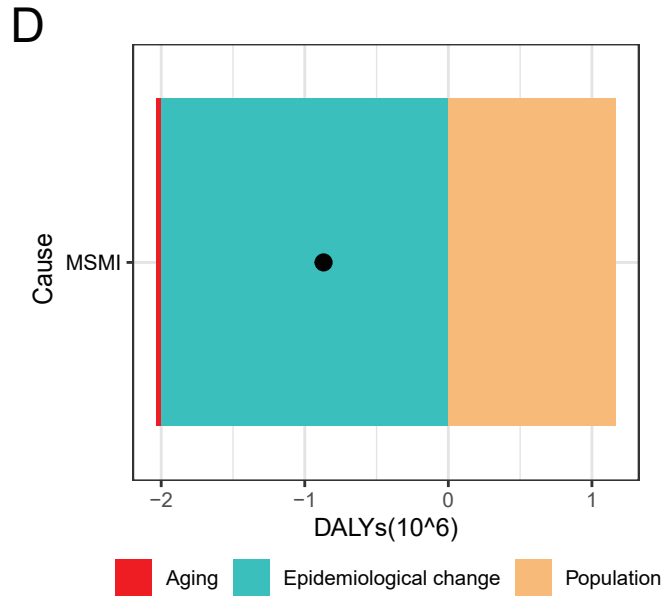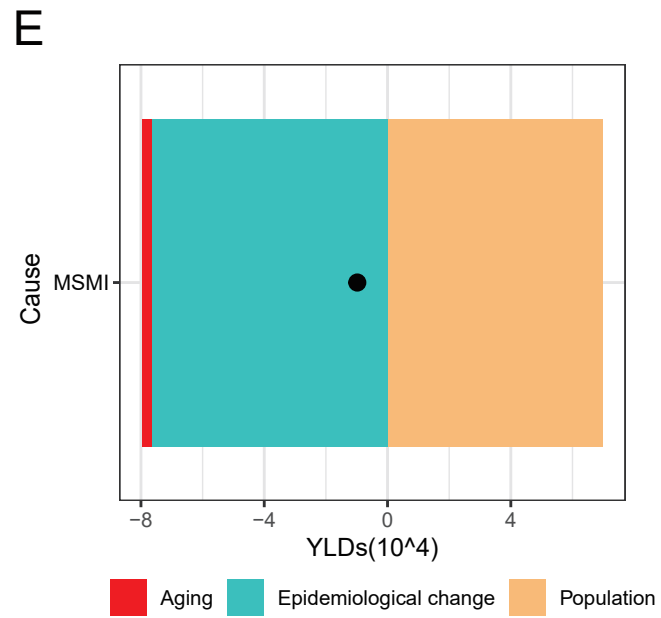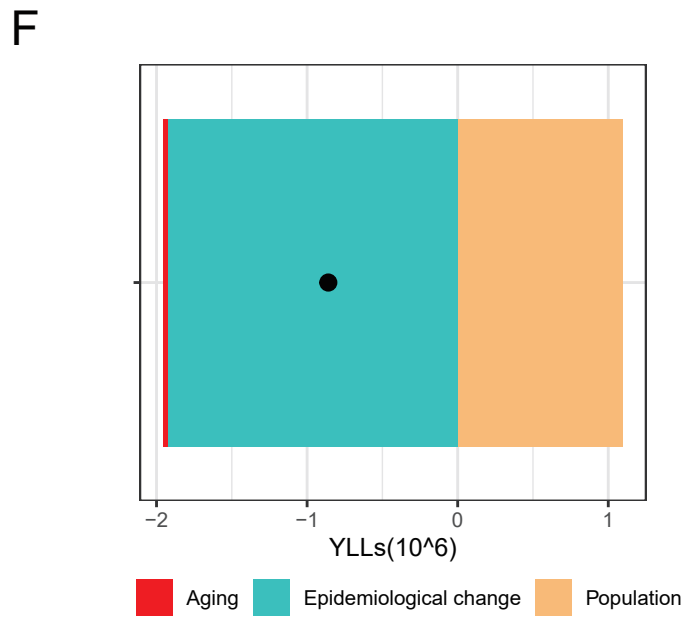

Supplement: S3 Fig — Three SDI components include population structure (aging), epidemiological changes, and population size (population). DALYs, Disability-Adjusted Life Years; YLDs, Years Lived with Disability; YLLs, Years of Life Lost. (PDF) [file pntd.0014374.s003.pdf]

A

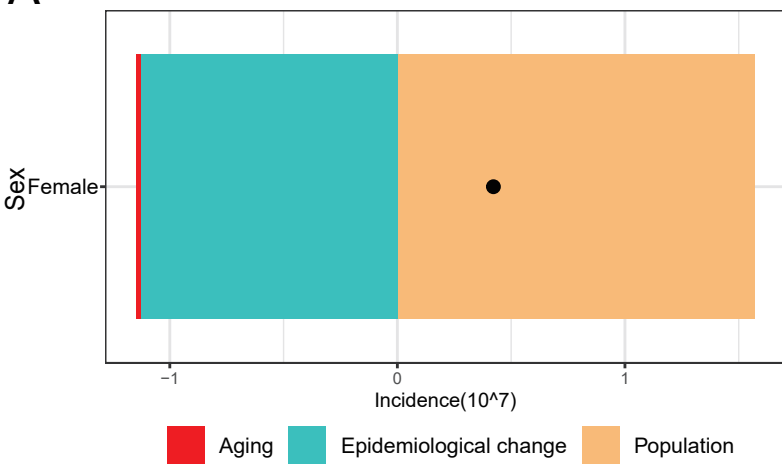

B

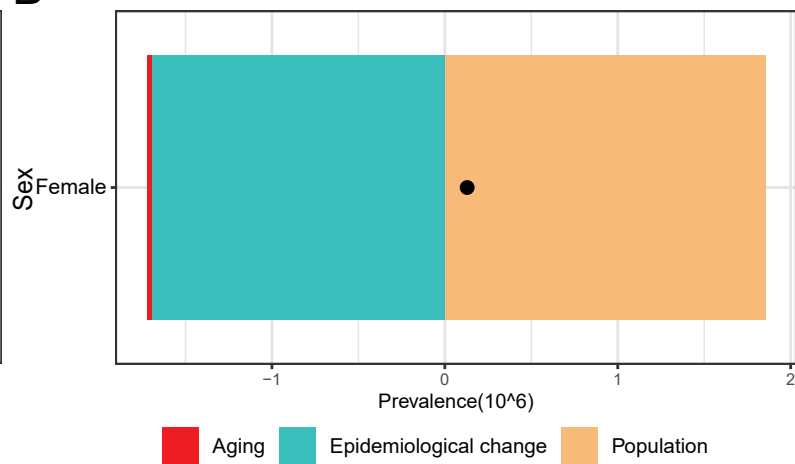

C

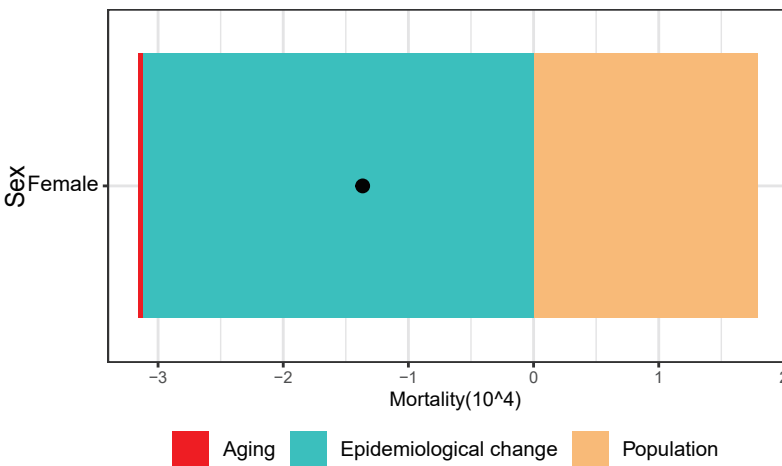

D

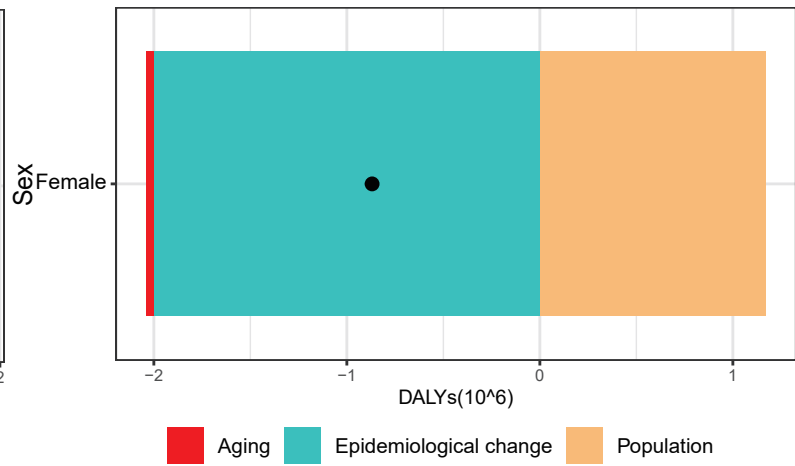

E

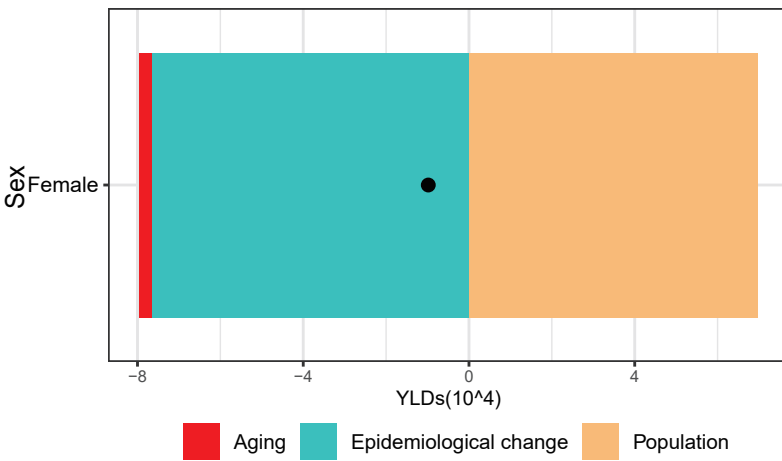

F

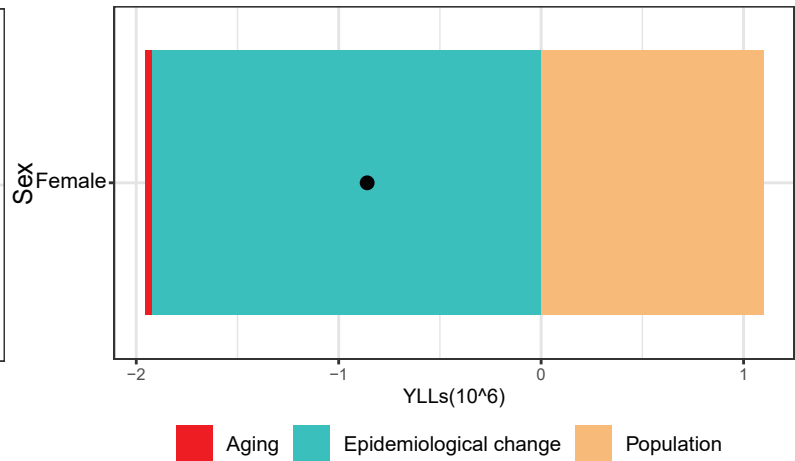

Supplement: S4 Fig — Three SDI components include population structure (aging), epidemiological changes, and population size (population). DALYs, Disability-Adjusted Life Years; YLDs, Years Lived with Disability; YLLs, Years of Life Lost. (PDF) [file pntd.0014374.s004.pdf]
