# Supplementary material for: Global burden, projections, and causal factors of maternal sepsis and other maternal infections: A comprehensive epidemiological and mendelian randomization study
Source: PLoS Negl Trop Dis. 2026 May 27;20(5):e0014374. doi: 10.1371/journal.pntd.0014374 (PMC13229374; doi:10.1371/journal.pntd.0014374)
Supplement: S5 Fig — The consistency of results remains robust even after excluding individual genetic variants in each analysis, indicating a high level of reliability and stability in our findings. (PDF) [file pntd.0014374.s005.pdf]

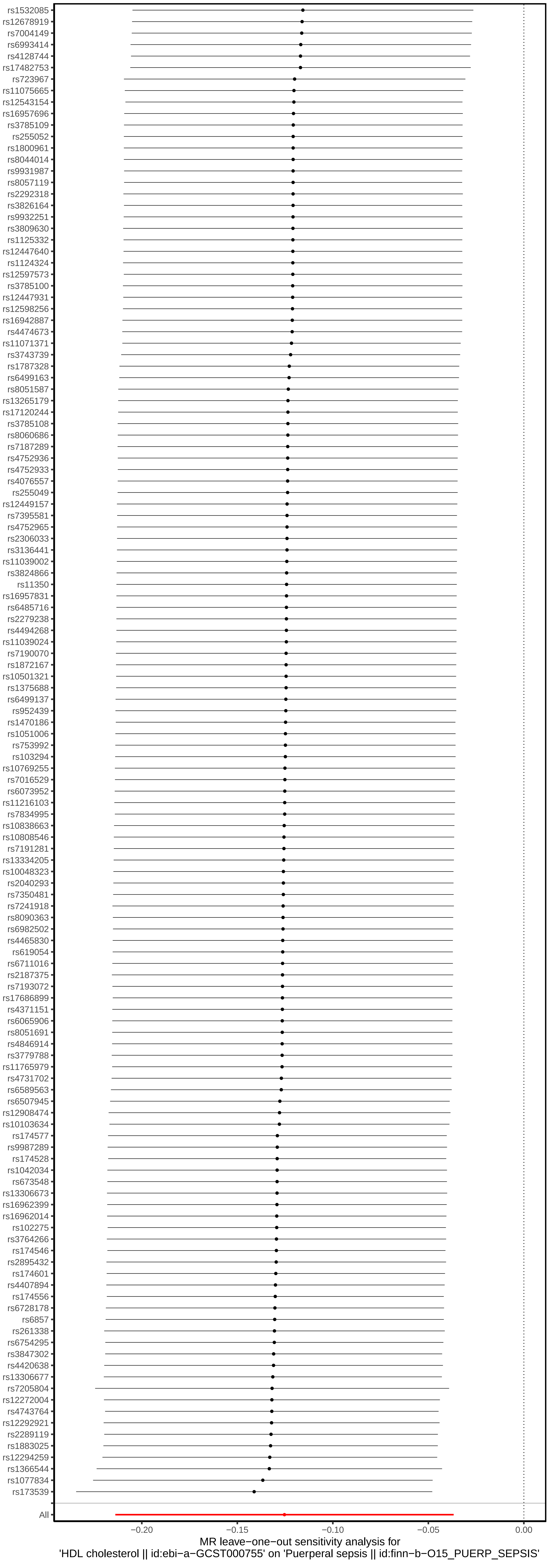
rs1532085
rs12678919
rs7004149
rs6993414
rs4128744
rs17482753
rs723967
rs11075665
rs12543154
rs16957696
rs3785109
rs255052
rs1800961
rs8044014
rs9931987
rs8057119
rs2292318
rs3826164
rs9932251
rs3809630
rs1125332
rs12447640
rs1124324
rs12597573
rs3785100
rs12447931
rs12598256
rs16942887
rs4474673
rs11071371
rs3743739
rs1787328
rs6499163
rs8051587
rs13265179
rs17120244
rs3785108
rs8060686
rs7187289
rs4752936
rs4752933
rs4076557
rs255049
rs12449157
rs7395581
rs4752965
rs2306033
rs3136441
rs11039002
rs3824866
rs11350
rs16957831
rs6485716
rs2279238
rs4494268
rs11039024
rs7190070
rs1872167
rs10501321
rs1375688
rs6499137
rs952439
rs1470186
rs1051006
rs753992
rs103294
rs10769255
rs7016529
rs6073952
rs11216103
rs7834995
rs10838663
rs10808546
rs7191281
rs13334205
rs10048323
rs2040293
rs7350481
rs7241918
rs8090363
rs6982502
rs4465830
rs619054
rs6711016
rs2187375
rs7193072
rs17686899
rs4371151
rs6065906
rs8051691
rs4846914
rs3779788
rs11765979
rs4731702
rs6589563
rs6507945
rs12908474
rs10103634
rs174577
rs9987289
rs174528
rs1042034
rs673548
rs13306673
rs16962399
rs16962014
rs102275
rs3764266
rs174546
rs2895432
rs174601
rs4407894
rs174556
rs6728178
rs6857
rs261338
rs6754295
rs3847302
rs4420638
rs13306677
rs7205804
rs12272004
rs4743764
rs12292921
rs2289119
rs1883025
rs12294259
rs1366544
rs1077834
rs173539
All
−0.20
−0.15
−0.10
−0.05
0.00
MR leave−one−out sensitivity analysis for
'HDL cholesterol || id:ebi−a−GCST000755' on 'Puerperal sepsis || id:finn−b−O15_PUERP_SEPSIS'

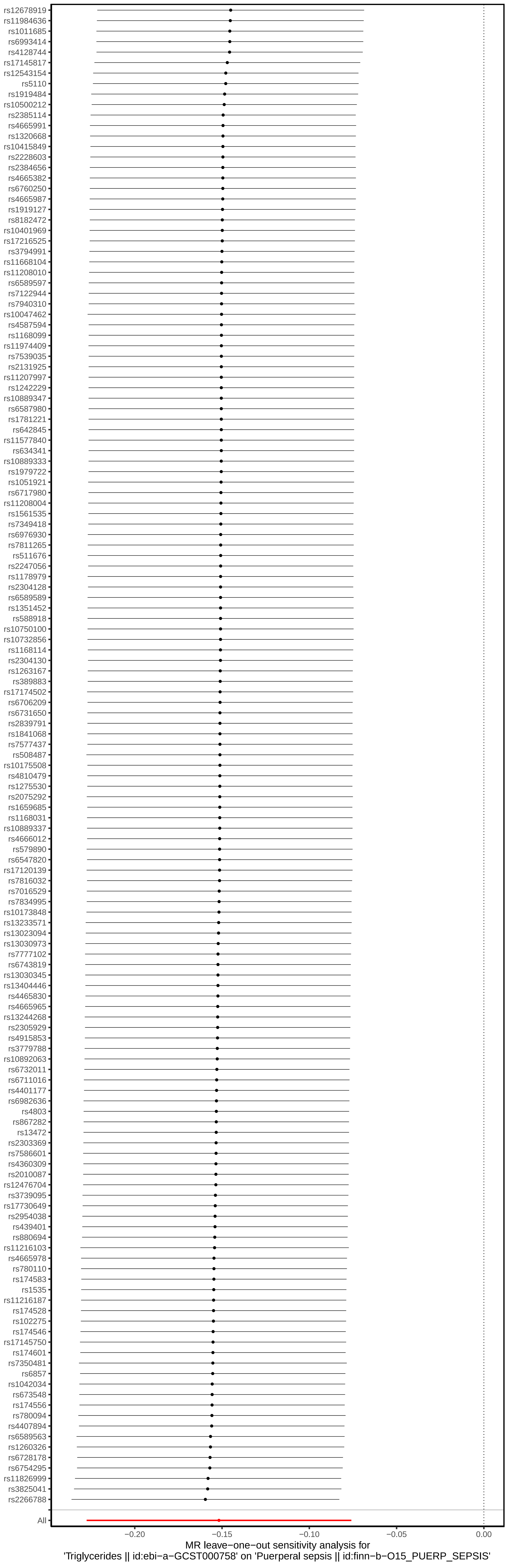
rs12678919
rs11984636
rs1011685
rs6993414
rs4128744
rs17145817
rs12543154
rs5110
rs1919484
rs10500212
rs2385114
rs4665991
rs1320668
rs10415849
rs2228603
rs2384656
rs4665382
rs6760250
rs4665987
rs1919127
rs8182472
rs10401969
rs17216525
rs3794991
rs11668104
rs11208010
rs6589597
rs7122944
rs7940310
rs10047462
rs4587594
rs1168099
rs11974409
rs7539035
rs2131925
rs11207997
rs1242229
rs10889347
rs6587980
rs1781221
rs642845
rs11577840
rs634341
rs10889333
rs1979722
rs1051921
rs6717980
rs11208004
rs1561535
rs7349418
rs6976930
rs7811265
rs511676
rs2247056
rs1178979
rs2304128
rs6589589
rs1351452
rs588918
rs10750100
rs10732856
rs1168114
rs2304130
rs1263167
rs389883
rs17174502
rs6706209
rs6731650
rs2839791
rs1841068
rs7577437
rs508487
rs10175508
rs4810479
rs1275530
rs2075292
rs1659685
rs1168031
rs10889337
rs4666012
rs579890
rs6547820
rs17120139
rs7816032
rs7016529
rs7834995
rs10173848
rs13233571
rs13023094
rs13030973
rs7777102
rs6743819
rs13030345
rs13404446
rs4465830
rs4665965
rs13244268
rs2305929
rs4915853
rs3779788
rs10892063
rs6732011
rs6711016
rs4401177
rs6982636
rs4803
rs867282
rs13472
rs2303369
rs7586601
rs4360309
rs2010087
rs12476704
rs3739095
rs17730649
rs2954038
rs439401
rs880694
rs11216103
rs4665978
rs780110
rs174583
rs1535
rs11216187
rs174528
rs102275
rs174546
rs17145750
rs174601
rs7350481
rs6857
rs1042034
rs673548
rs174556
rs780094
rs4407894
rs6589563
rs1260326
rs6728178
rs6754295
rs11826999
rs3825041
rs2266788
All
−0.20
−0.15
−0.10
−0.05
0.00
MR leave−one−out sensitivity analysis for
'Triglycerides || id:ebi−a−GCST000758' on 'Puerperal sepsis || id:finn−b−O15_PUERP_SEPSIS'

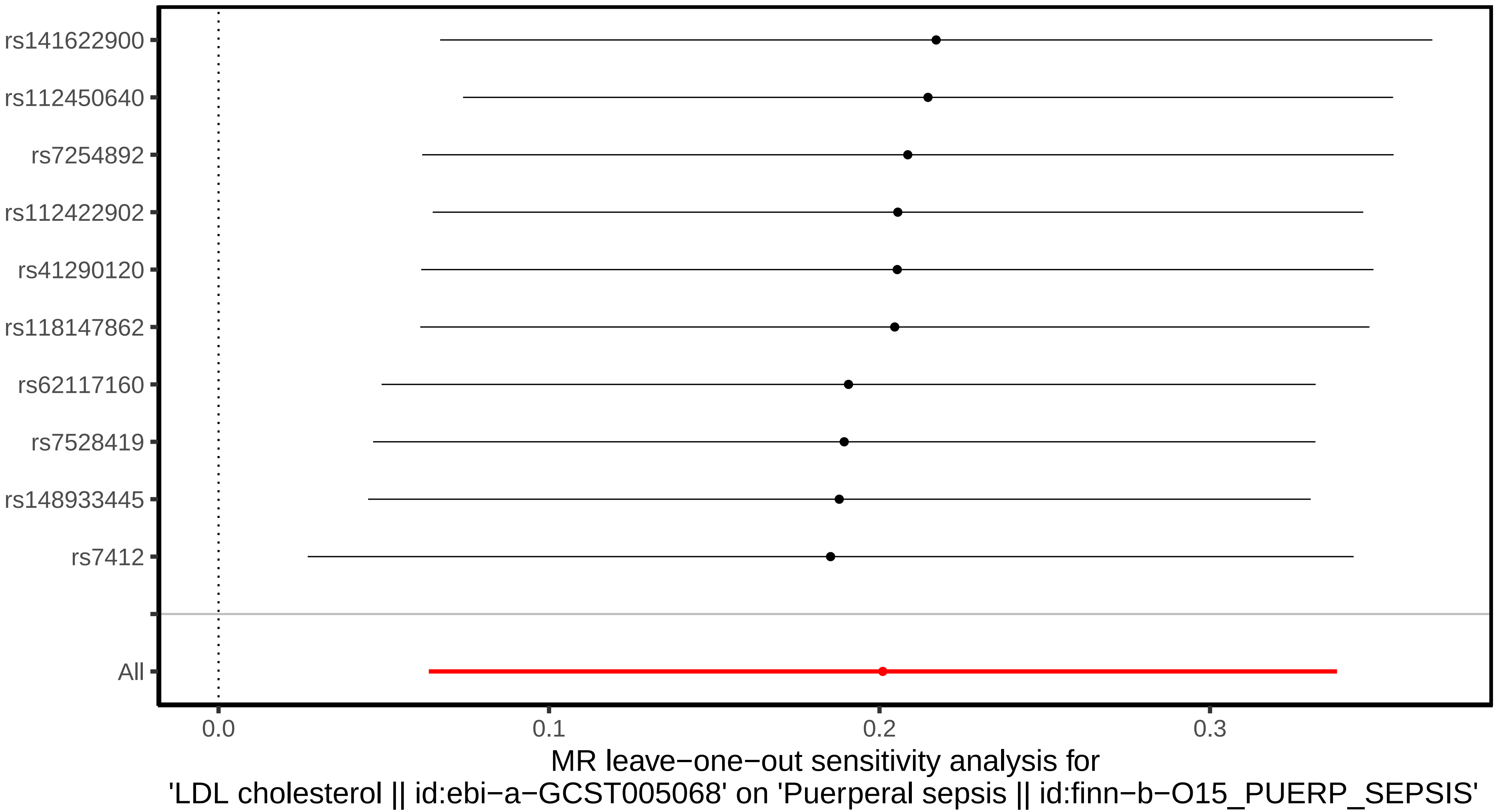
rs141622900
rs112450640
rs7254892
rs112422902
rs41290120
rs118147862
rs62117160
rs7528419
rs148933445
rs7412
All
0.0
0.1
0.2
0.3
MR leave−one−out sensitivity analysis for
'LDL cholesterol || id:ebi−a−GCST005068' on 'Puerperal sepsis || id:finn−b−O15_PUERP_SEPSIS'

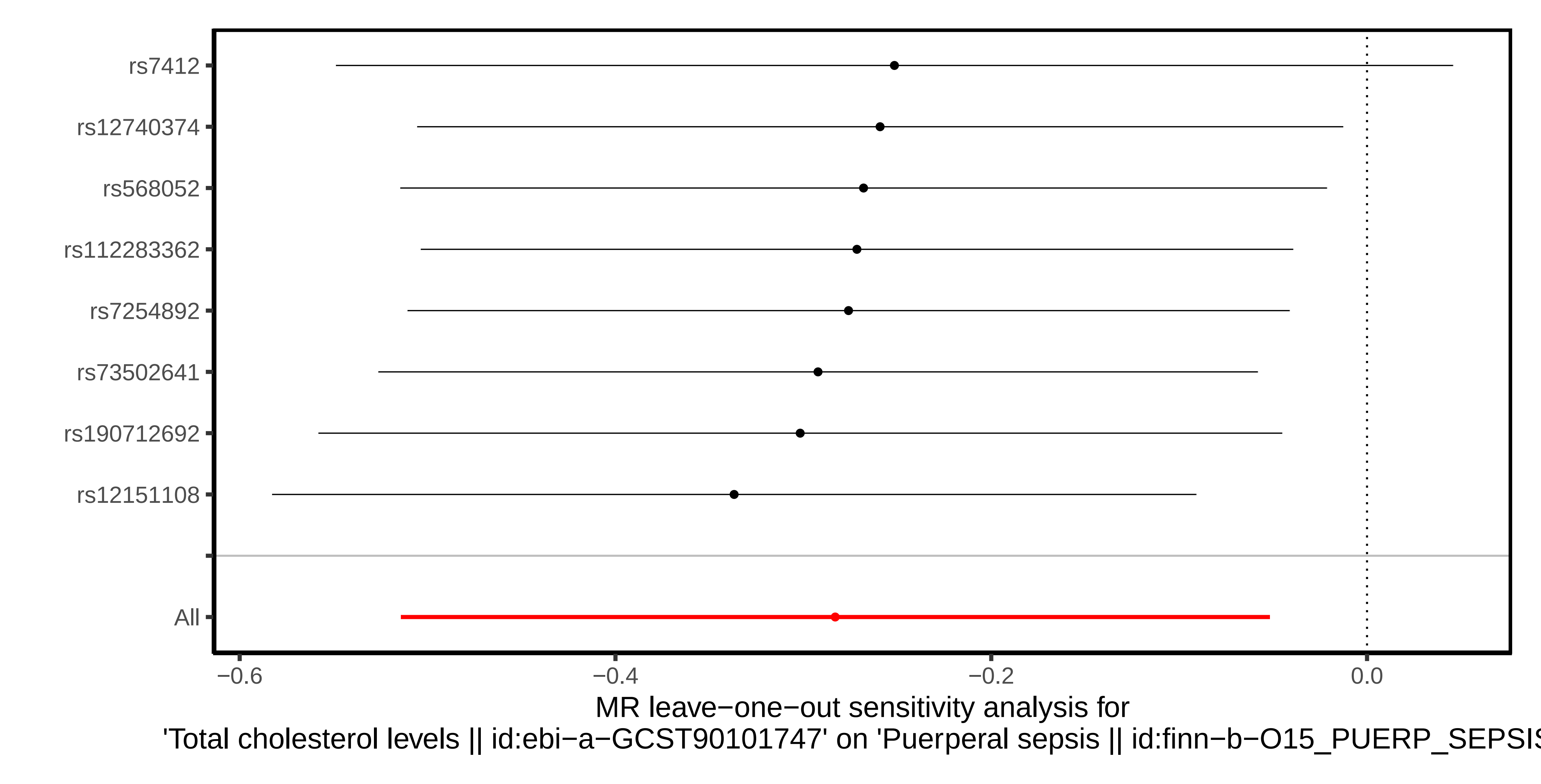
rs7412
rs12740374
rs568052
rs112283362
rs7254892
rs73502641
rs190712692
rs12151108
All
−0.6
−0.4
−0.2
0.0
MR leave−one−out sensitivity analysis for
'Total cholesterol levels || id:ebi−a−GCST90101747' on 'Puerperal sepsis || id:finn−b−O15_PUERP_SEPSIS

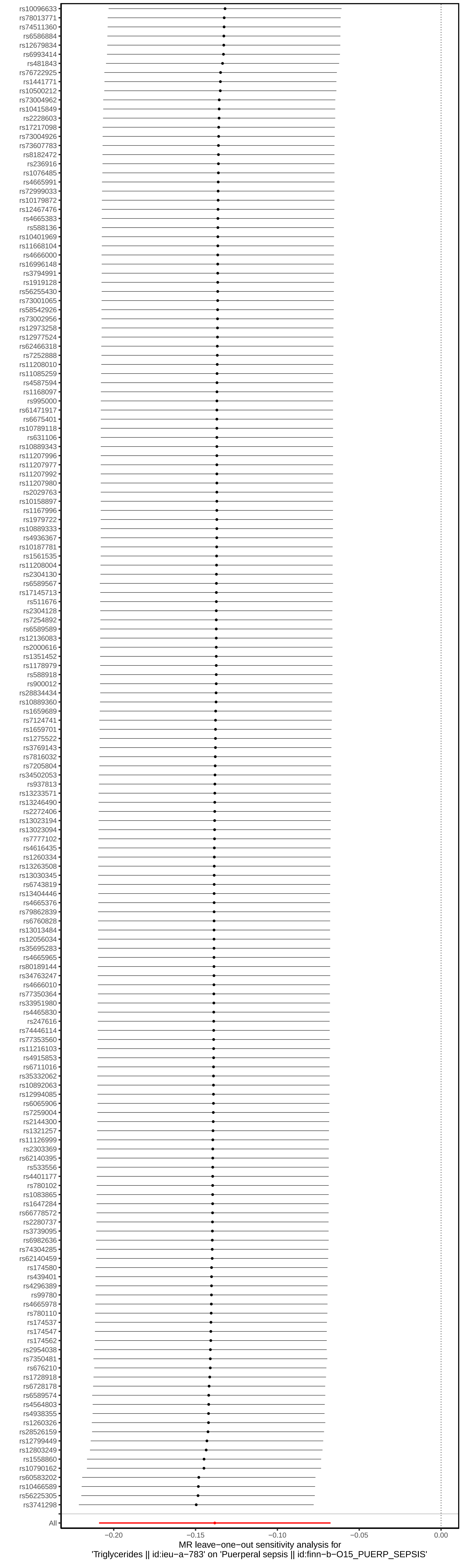
rs10096633
rs78013771
rs74511360
rs6586884
rs12679834
rs6993414
rs481843
rs76722925
rs1441771
rs10500212
rs73004962
rs10415849
rs2228603
rs17217098
rs73004926
rs73607783
rs8182472
rs236916
rs1076485
rs4665991
rs72999033
rs10179872
rs12467476
rs4665383
rs588136
rs10401969
rs11668104
rs4666000
rs16996148
rs3794991
rs1919128
rs56255430
rs73001065
rs58542926
rs73002956
rs12973258
rs12977524
rs62466318
rs7252888
rs11208010
rs11085259
rs4587594
rs1168097
rs995000
rs61471917
rs6675401
rs10789118
rs631106
rs10889343
rs11207996
rs11207977
rs11207992
rs11207980
rs2029763
rs10158897
rs1167996
rs1979722
rs10889333
rs4936367
rs10187781
rs1561535
rs11208004
rs2304130
rs6589567
rs17145713
rs511676
rs2304128
rs7254892
rs6589589
rs12136083
rs2000616
rs1351452
rs1178979
rs588918
rs900012
rs28834434
rs10889360
rs1659689
rs7124741
rs1659701
rs1275522
rs3769143
rs7816032
rs7205804
rs34502053
rs937813
rs13233571
rs13246490
rs2272406
rs13023194
rs13023094
rs7777102
rs4616435
rs1260334
rs13263508
rs13030345
rs6743819
rs13404446
rs4665376
rs79862839
rs6760828
rs13013484
rs12056034
rs35695283
rs4665965
rs80189144
rs34763247
rs4666010
rs77350364
rs33951980
rs4465830
rs247616
rs74446114
rs77353560
rs11216103
rs4915853
rs6711016
rs35332062
rs10892063
rs12994085
rs6065906
rs7259004
rs2144300
rs1321257
rs11126999
rs2303369
rs62140395
rs533556
rs4401177
rs780102
rs1083865
rs1647284
rs66778572
rs2280737
rs3739095
rs6982636
rs74304285
rs62140459
rs174580
rs439401
rs4296389
rs99780
rs4665978
rs780110
rs174537
rs174547
rs174562
rs2954038
rs7350481
rs676210
rs1728918
rs6728178
rs6589574
rs4564803
rs4938355
rs1260326
rs28526159
rs12799449
rs12803249
rs1558860
rs10790162
rs60583202
rs10466589
rs56225305
rs3741298
All
−0.20
−0.15
−0.10
−0.05
0.00
MR leave−one−out sensitivity analysis for
'Triglycerides || id:ieu−a−783' on 'Puerperal sepsis || id:finn−b−O15_PUERP_SEPSIS'

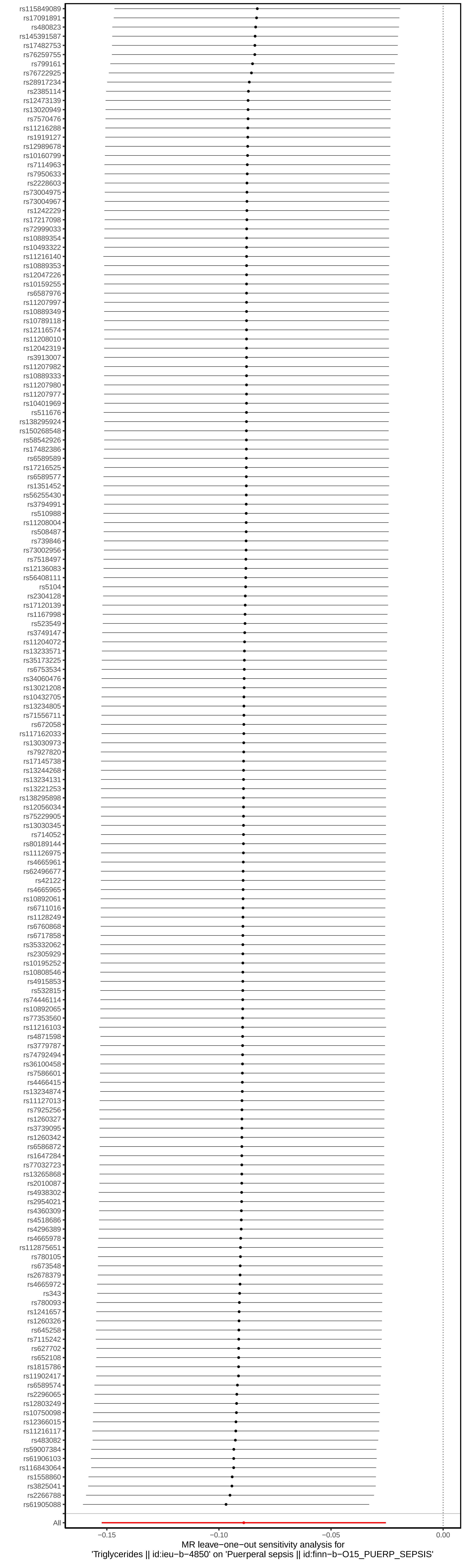
rs115849089
rs17091891
rs480823
rs145391587
rs17482753
rs76259755
rs799161
rs76722925
rs28917234
rs2385114
rs12473139
rs13020949
rs7570476
rs11216288
rs1919127
rs12989678
rs10160799
rs7114963
rs7950633
rs2228603
rs73004975
rs73004967
rs1242229
rs17217098
rs72999033
rs10889354
rs10493322
rs11216140
rs10889353
rs12047226
rs10159255
rs6587976
rs11207997
rs10889349
rs10789118
rs12116574
rs11208010
rs12042319
rs3913007
rs11207982
rs10889333
rs11207980
rs11207977
rs10401969
rs511676
rs138295924
rs150268548
rs58542926
rs17482386
rs6589589
rs17216525
rs6589577
rs1351452
rs56255430
rs3794991
rs510988
rs11208004
rs508487
rs739846
rs73002956
rs7518497
rs12136083
rs56408111
rs5104
rs2304128
rs17120139
rs1167998
rs523549
rs3749147
rs11204072
rs13233571
rs35173225
rs6753534
rs34060476
rs13021208
rs10432705
rs13234805
rs71556711
rs672058
rs117162033
rs13030973
rs7927820
rs17145738
rs13244268
rs13234131
rs13221253
rs138295898
rs12056034
rs75229905
rs13030345
rs714052
rs80189144
rs11126975
rs4665961
rs62496677
rs42122
rs4665965
rs10892061
rs6711016
rs1128249
rs6760868
rs6717858
rs35332062
rs2305929
rs10195252
rs10808546
rs4915853
rs532815
rs74446114
rs10892065
rs77353560
rs11216103
rs4871598
rs3779787
rs74792494
rs36100458
rs7586601
rs4466415
rs13234874
rs11127013
rs7925256
rs1260327
rs3739095
rs1260342
rs6586872
rs1647284
rs77032723
rs13265868
rs2010087
rs4938302
rs2954021
rs4360309
rs4518686
rs4296389
rs4665978
rs112875651
rs780105
rs673548
rs2678379
rs4665972
rs343
rs780093
rs1241657
rs1260326
rs645258
rs7115242
rs627702
rs652108
rs1815786
rs11902417
rs6589574
rs2296065
rs12803249
rs10750098
rs12366015
rs11216117
rs483082
rs59007384
rs61906103
rs116843064
rs1558860
rs3825041
rs2266788
rs61905088
All
-0.15
-0.10
-0.05
0.00
MR leave−one−out sensitivity analysis for
'Triglycerides || id:ieu−b−4850' on 'Puerperal sepsis || id:finn−b−O15_PUERP_SEPSIS'

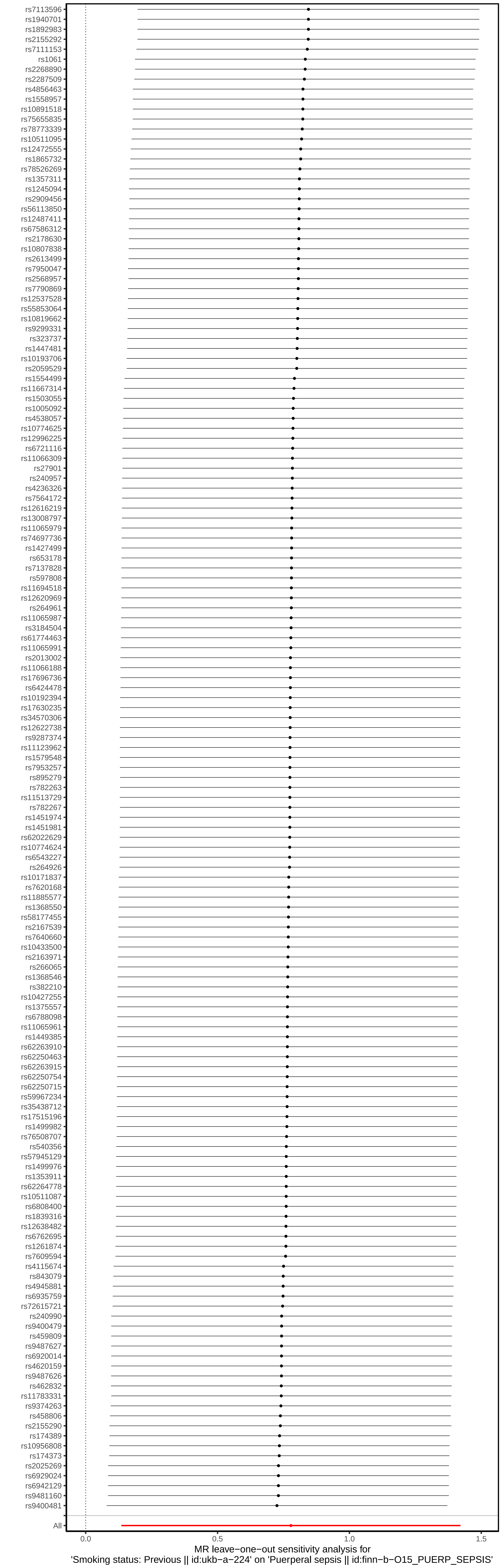
rs7113596
rs1940701
rs1892983
rs2155292
rs7111153
rs1061
rs2268890
rs2287509
rs4856463
rs1558957
rs10891518
rs75655835
rs78773339
rs10511095
rs12472555
rs1865732
rs78526269
rs1357311
rs1245094
rs2909456
rs56113850
rs12487411
rs67586312
rs2178630
rs10807838
rs2613499
rs7950047
rs2568957
rs7790869
rs12537528
rs55853064
rs10819662
rs9299331
rs323737
rs1447481
rs10193706
rs2059529
rs1554499
rs11667314
rs1503055
rs1005092
rs4538057
rs10774625
rs12996225
rs6721116
rs11066309
rs27901
rs240957
rs4236326
rs7564172
rs12616219
rs13008797
rs11065979
rs74697736
rs1427499
rs653178
rs7137828
rs597808
rs11694518
rs12620969
rs264961
rs11065987
rs3184504
rs61774463
rs11065991
rs2013002
rs11066188
rs17696736
rs6424478
rs10192394
rs17630235
rs34570306
rs12622738
rs9287374
rs11123962
rs1579548
rs7953257
rs895279
rs782263
rs11513729
rs782267
rs1451974
rs1451981
rs62022629
rs10774624
rs6543227
rs264926
rs10171837
rs7620168
rs11885577
rs1368550
rs58177455
rs2167539
rs7640660
rs10433500
rs2163971
rs266065
rs1368546
rs382210
rs10427255
rs1375557
rs6788098
rs11065961
rs1449385
rs62263910
rs62250463
rs62263915
rs62250754
rs62250715
rs59967234
rs35438712
rs17515196
rs1499982
rs76508707
rs540356
rs57945129
rs1499976
rs1353911
rs62264778
rs10511087
rs6808400
rs1839316
rs12638482
rs6762695
rs1261874
rs7609594
rs4115674
rs843079
rs4945881
rs6935759
rs72615721
rs240990
rs9400479
rs459809
rs9487627
rs6920014
rs4620159
rs9487626
rs462832
rs11783331
rs9374263
rs458806
rs2155290
rs174389
rs10956808
rs174373
rs2025269
rs6929024
rs6942129
rs9481160
rs9400481
All
0.0
0.5
1.0
1.5
MR leave−one−out sensitivity analysis for
'Smoking status: Previous || id:ukb−a−224' on 'Puerperal sepsis || id:finn−b−O15_PUERP_SEPSIS'

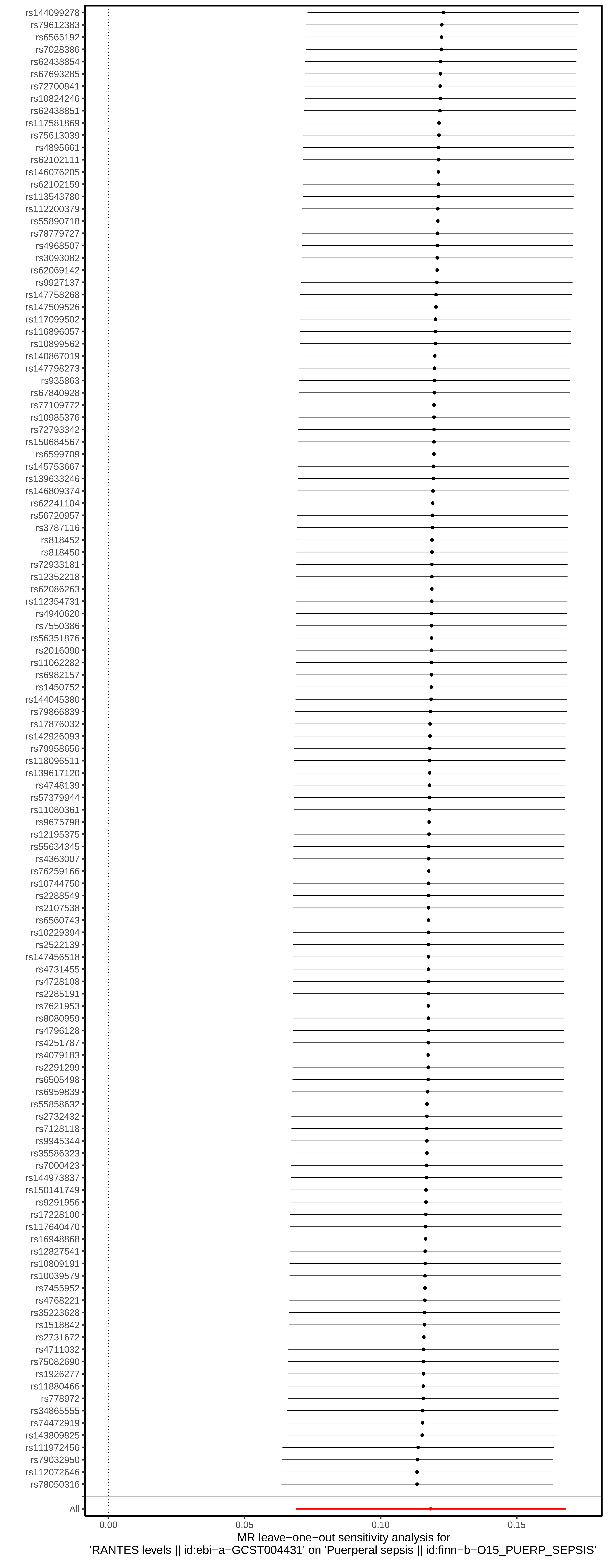

rs144099278
rs79612383
rs6565192
rs7028386
rs62438854
rs67693285
rs72700841
rs10824246
rs62438851
rs117581869
rs75613039
rs4895661
rs62102111
rs146076205
rs62102159
rs113543780
rs112200379
rs55890718
rs78779727
rs4968507
rs3093082
rs62069142
rs9927137
rs147758268
rs147509526
rs117099502
rs116896057
rs10899562
rs140867019
rs147798273
rs935863
rs67840928
rs77109772
rs10985376
rs72793342
rs150684567
rs6599709
rs145753667
rs139633246
rs146809374
rs62241104
rs56720957
rs3787116
rs818452
rs818450
rs72933181
rs12352218
rs62086263
rs112354731
rs4940620
rs7550386
rs56351876
rs2016090
rs11062282
rs6982157
rs1450752
rs144045380
rs79866839
rs17876032
rs142926093
rs79958656
rs118096511
rs139617120
rs4748139
rs57379944
rs11080361
rs9675798
rs12195375
rs55634345
rs4363007
rs76259166
rs10744750
rs2288549
rs2107538
rs6560743
rs10229394
rs2522139
rs147456518
rs4731455
rs4728108
rs2285191
rs7621953
rs8080959
rs4796128
rs4251787
rs4079183
rs2291299
rs6505498
rs6959839
rs55858632
rs2732432
rs7128118
rs9945344
rs35586323
rs7000423
rs144973837
rs150141749
rs9291956
rs17228100
rs117640470
rs16948868
rs12827541
rs10809191
rs10039579
rs7455952
rs4768221
rs35223628
rs1518842
rs2731672
rs4711032
rs75082690
rs1926277
rs11880466
rs778972
rs34865555
rs74472919
rs143809825
rs111972456
rs79032950
rs112072646
rs78050316
All
0.00
0.05
0.10
0.15
MR leave−one−out sensitivity analysis for
'RANTES levels || id:ebi−a−GCST004431' on 'Puerperal sepsis || id:finn−b−O15_PUERP_SEPSIS'

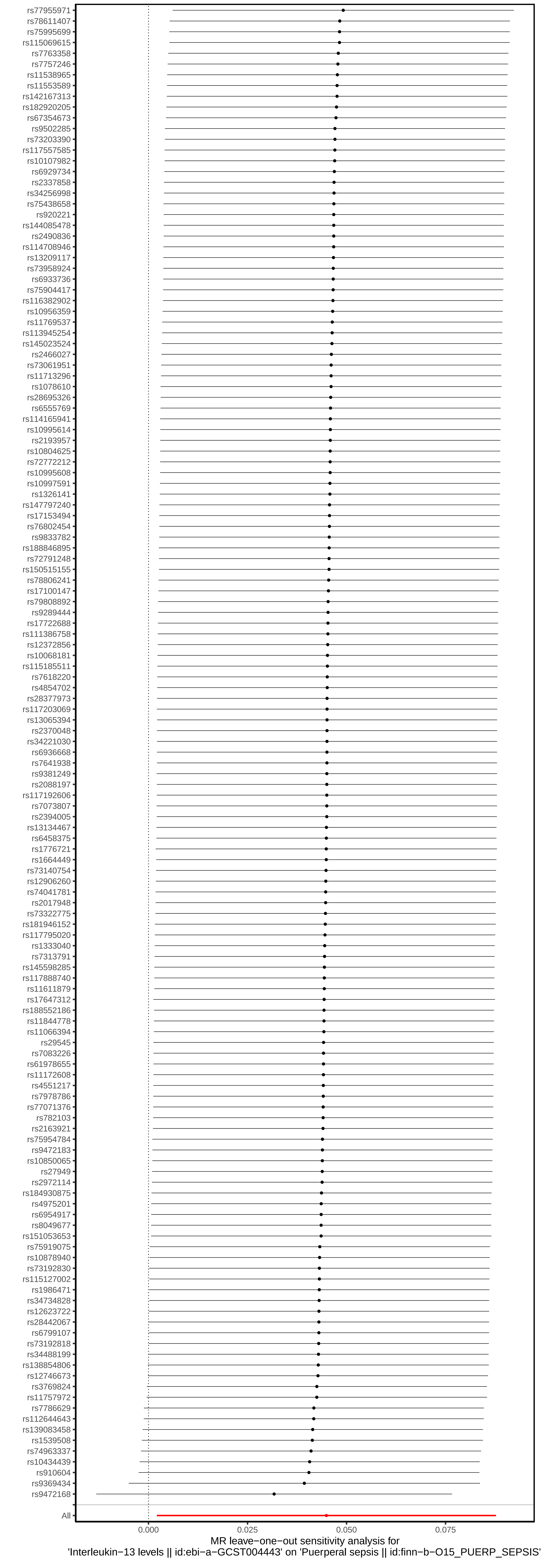

rs77955971
rs78611407
rs75995699
rs115069615
rs7763358
rs7757246
rs11538965
rs11553589
rs142167313
rs182920205
rs67354673
rs9502285
rs73203390
rs117557585
rs10107982
rs6929734
rs2337858
rs34256998
rs75438658
rs920221
rs144085478
rs2490836
rs114708946
rs13209117
rs73958924
rs6933736
rs75904417
rs116382902
rs10956359
rs11769537
rs113945254
rs145023524
rs2466027
rs73061951
rs11713296
rs1078610
rs28695326
rs6555769
rs114165941
rs10995614
rs2193957
rs10804625
rs72772212
rs10995608
rs10997591
rs1326141
rs147797240
rs17153494
rs76802454
rs9833782
rs188846895
rs72791248
rs150515155
rs78806241
rs17100147
rs79808892
rs9289444
rs17722688
rs111386758
rs12372856
rs10068181
rs115185511
rs7618220
rs4854702
rs28377973
rs117203069
rs13065394
rs2370048
rs34221030
rs6936668
rs7641938
rs9381249
rs2088197
rs117192606
rs7073807
rs2394005
rs13134467
rs6458375
rs1776721
rs1664449
rs73140754
rs12906260
rs74041781
rs2017948
rs73322775
rs181946152
rs117795020
rs1333040
rs7313791
rs145598285
rs117888740
rs11611879
rs17647312
rs188552186
rs11844778
rs11066394
rs29545
rs7083226
rs61978655
rs11172608
rs4551217
rs7978786
rs77071376
rs782103
rs2163921
rs75954784
rs9472183
rs10850065
rs27949
rs2972114
rs184930875
rs4975201
rs6954917
rs8049677
rs151053653
rs75919075
rs10878940
rs73192830
rs115127002
rs1986471
rs34734828
rs12623722
rs28442067
rs6799107
rs73192818
rs34488199
rs138854806
rs12746673
rs3769824
rs11757972
rs7786629
rs112644643
rs139083458
rs1539508
rs74963337
rs10434439
rs910604
rs9369434
rs9472168
All
0.000
0.025
0.050
0.075
MR leave−one−out sensitivity analysis for
'Interleukin−13 levels || id:ebi−a−GCST004443' on 'Puerperal sepsis || id:finn−b−O15_PUERP_SEPSIS'

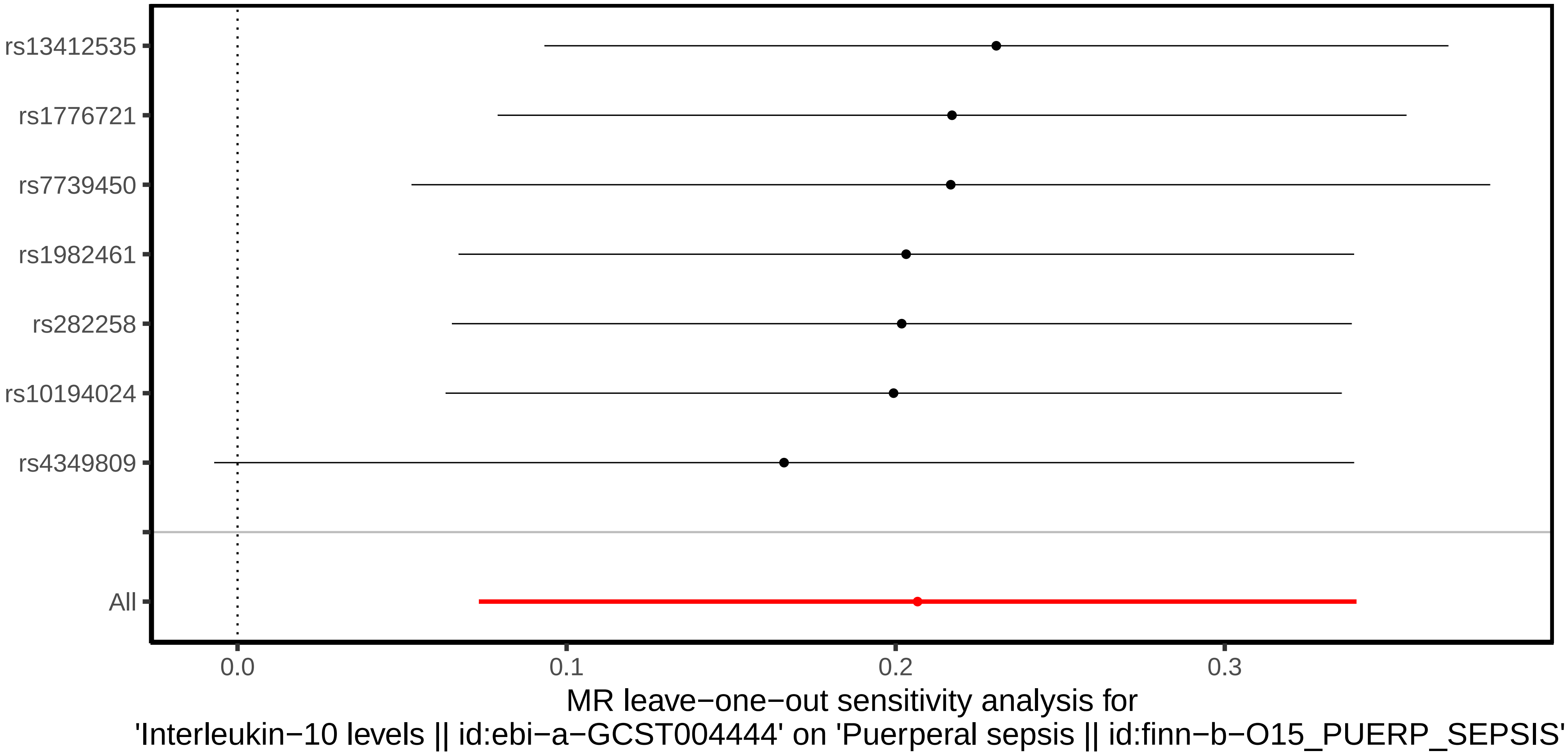
rs13412535
rs1776721
rs7739450
rs1982461
rs282258
rs10194024
rs4349809
All
0.0
0.1
0.2
0.3
MR leave−one−out sensitivity analysis for
'Interleukin−10 levels || id:ebi−a−GCST004444' on 'Puerperal sepsis || id:finn−b−O15_PUERP_SEPSIS'

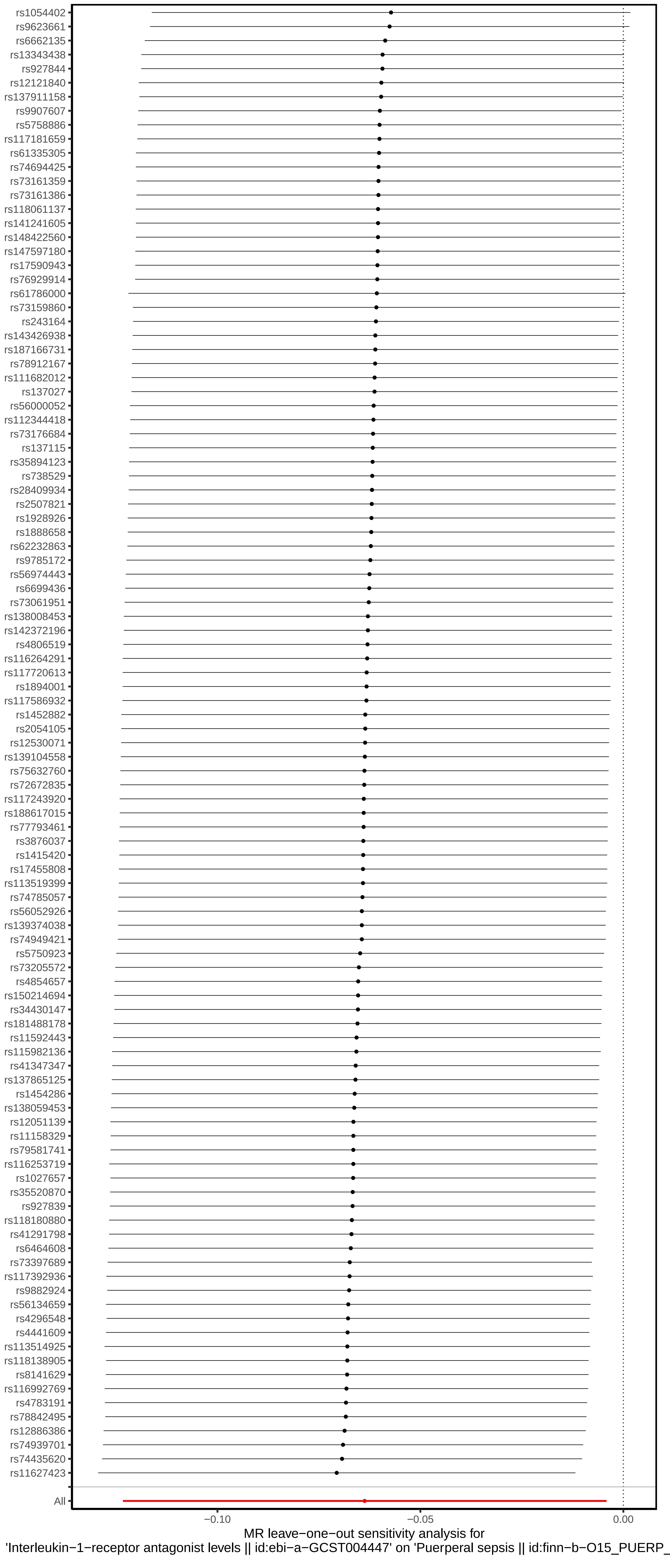
rs1054402
rs9623661
rs6662135
rs13343438
rs927844
rs12121840
rs137911158
rs9907607
rs5758886
rs117181659
rs61335305
rs74694425
rs73161359
rs73161386
rs118061137
rs141241605
rs148422560
rs147597180
rs17590943
rs76929914
rs61786000
rs73159860
rs243164
rs143426938
rs187166731
rs78912167
rs111682012
rs137027
rs56000052
rs112344418
rs73176684
rs137115
rs35894123
rs738529
rs28409934
rs2507821
rs1928926
rs1888658
rs62232863
rs9785172
rs56974443
rs6699436
rs73061951
rs138008453
rs142372196
rs4806519
rs116264291
rs117720613
rs1894001
rs117586932
rs1452882
rs2054105
rs12530071
rs139104558
rs75632760
rs72672835
rs117243920
rs188617015
rs77793461
rs3876037
rs1415420
rs17455808
rs113519399
rs74785057
rs56052926
rs139374038
rs74949421
rs5750923
rs73205572
rs4854657
rs150214694
rs34430147
rs181488178
rs11592443
rs115982136
rs41347347
rs137865125
rs1454286
rs138059453
rs12051139
rs11158329
rs79581741
rs116253719
rs1027657
rs35520870
rs927839
rs118180880
rs41291798
rs6464608
rs73397689
rs117392936
rs9882924
rs56134659
rs4296548
rs4441609
rs113514925
rs118138905
rs8141629
rs116992769
rs4783191
rs78842495
rs12886386
rs74939701
rs74435620
rs11627423
All
−0.10
−0.05
0.00
MR leave−one−out sensitivity analysis for
'Interleukin−1−receptor antagonist levels || id:ebi−a−GCST004447' on 'Puerperal sepsis || id:finn−b−O15_PUERP_

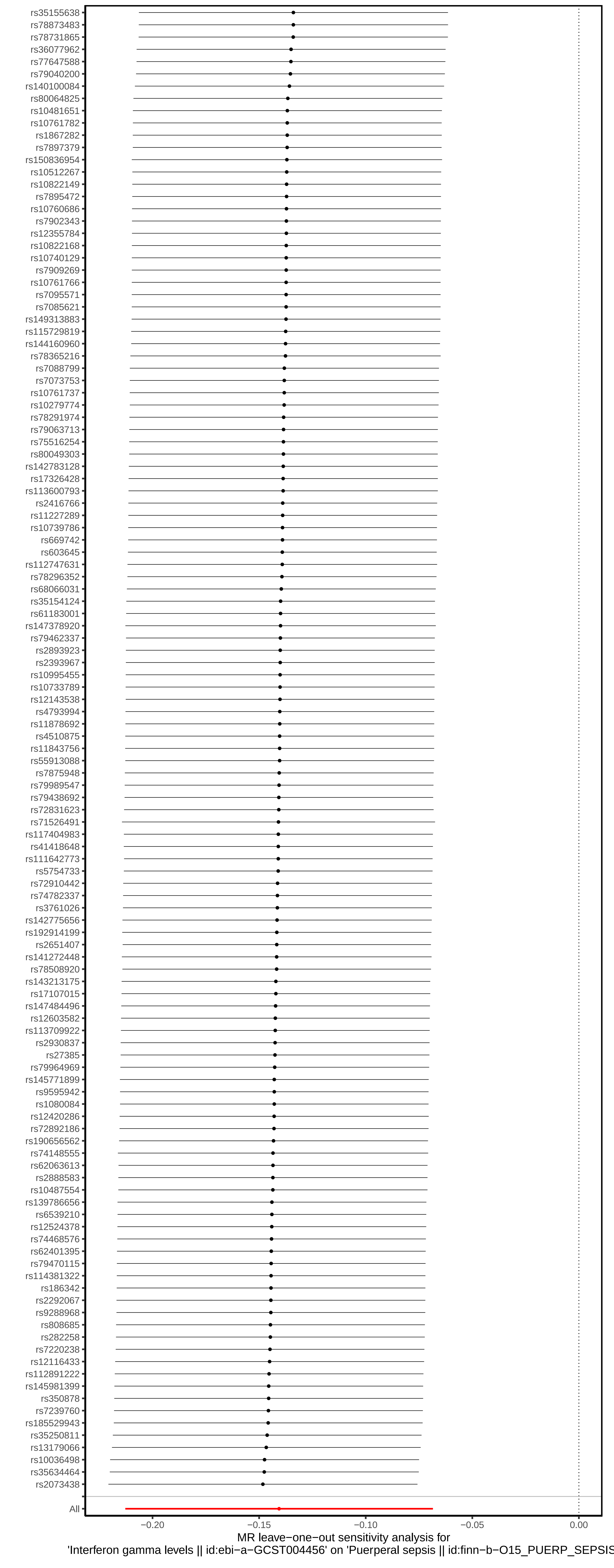
rs35155638
rs78873483
rs78731865
rs36077962
rs77647588
rs79040200
rs140100084
rs80064825
rs10481651
rs10761782
rs1867282
rs7897379
rs150836954
rs10512267
rs10822149
rs7895472
rs10760686
rs7902343
rs12355784
rs10822168
rs10740129
rs7909269
rs10761766
rs7095571
rs7085621
rs149313883
rs115729819
rs144160960
rs78365216
rs7088799
rs7073753
rs10761737
rs10279774
rs78291974
rs79063713
rs75516254
rs80049303
rs142783128
rs17326428
rs113600793
rs2416766
rs11227289
rs10739786
rs669742
rs603645
rs112747631
rs78296352
rs68066031
rs35154124
rs61183001
rs147378920
rs79462337
rs2893923
rs2393967
rs10995455
rs10733789
rs12143538
rs4793994
rs11878692
rs4510875
rs11843756
rs55913088
rs7875948
rs79989547
rs79438692
rs72831623
rs71526491
rs117404983
rs41418648
rs111642773
rs5754733
rs72910442
rs74782337
rs3761026
rs142775656
rs192914199
rs2651407
rs141272448
rs78508920
rs143213175
rs17107015
rs147484496
rs12603582
rs113709922
rs2930837
rs27385
rs79964969
rs145771899
rs9595942
rs1080084
rs12420286
rs72892186
rs190656562
rs74148555
rs62063613
rs2888583
rs10487554
rs139786656
rs6539210
rs12524378
rs74468576
rs62401395
rs79470115
rs114381322
rs186342
rs2292067
rs9288968
rs808685
rs282258
rs7220238
rs12116433
rs112891222
rs145981399
rs350878
rs7239760
rs185529943
rs35250811
rs13179066
rs10036498
rs35634464
rs2073438
All
−0.20
−0.15
−0.10
−0.05
0.00
MR leave−one−out sensitivity analysis for
'Interferon gamma levels || id:ebi−a−GCST004456' on 'Puerperal sepsis || id:finn−b−O15_PUERP_SEPSIS

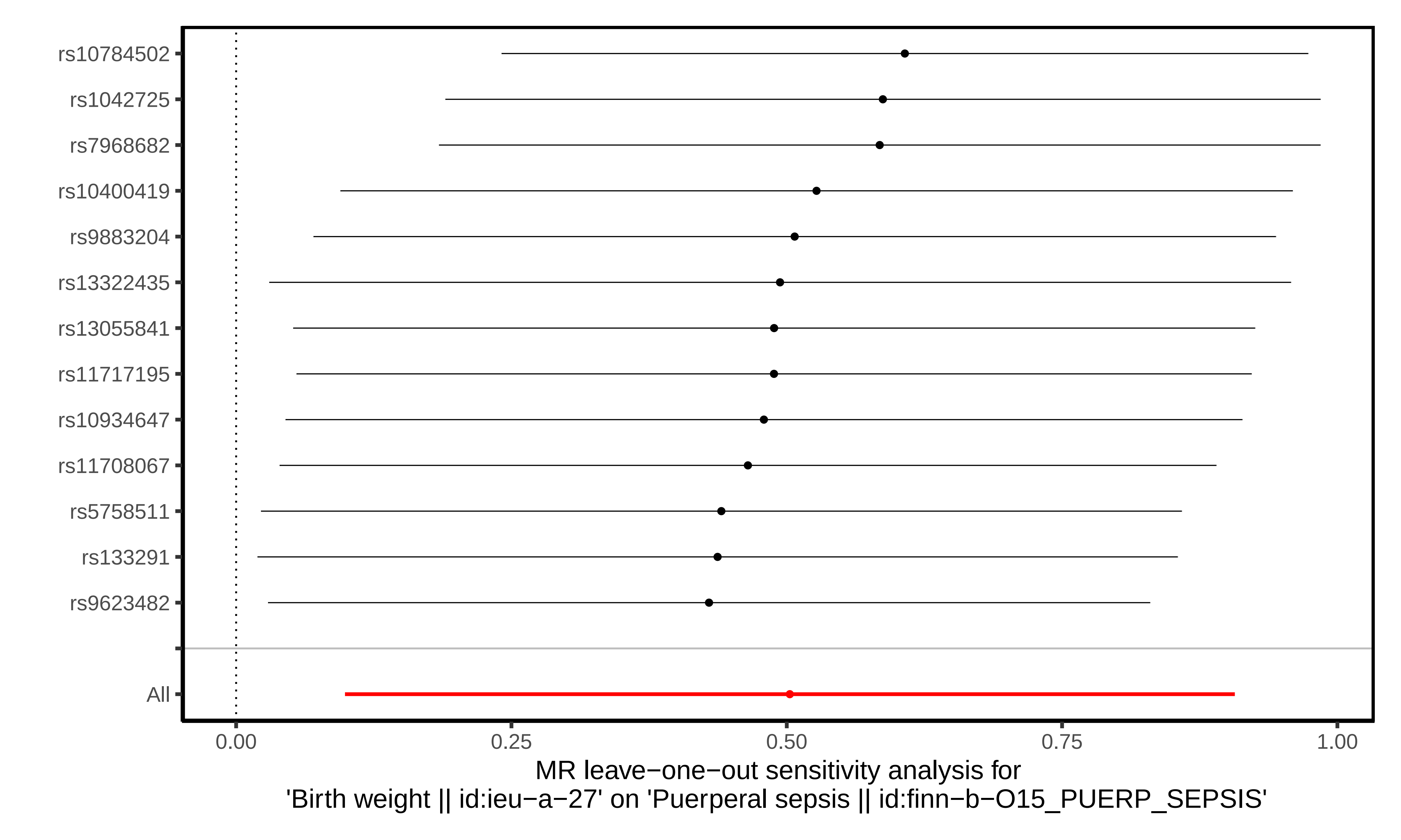
rs10784502
rs1042725
rs7968682
rs10400419
rs9883204
rs13322435
rs13055841
rs11717195
rs10934647
rs11708067
rs5758511
rs133291
rs9623482
All
0.00
0.25
0.50
0.75
1.00
MR leave−one−out sensitivity analysis for
'Birth weight || id:ieu−a−27' on 'Puerperal sepsis || id:finn−b−O15_PUERP_SEPSIS'

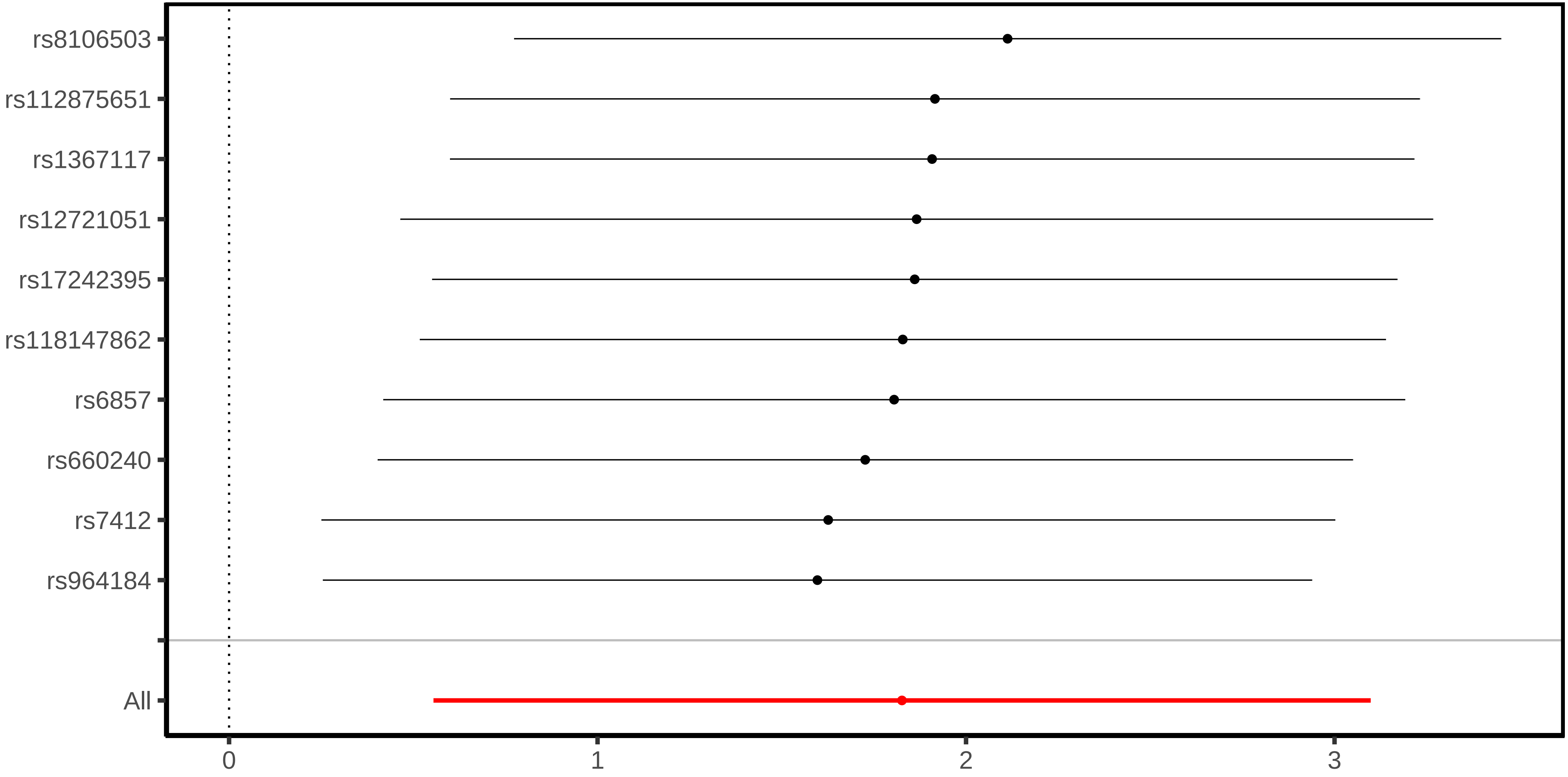

MR leave−one−out sensitivity analysis for
blood pressure diabetes or take exogenous hormones: Cholesterol lowering medication || id:ukb−a−448' on 'Puerperal

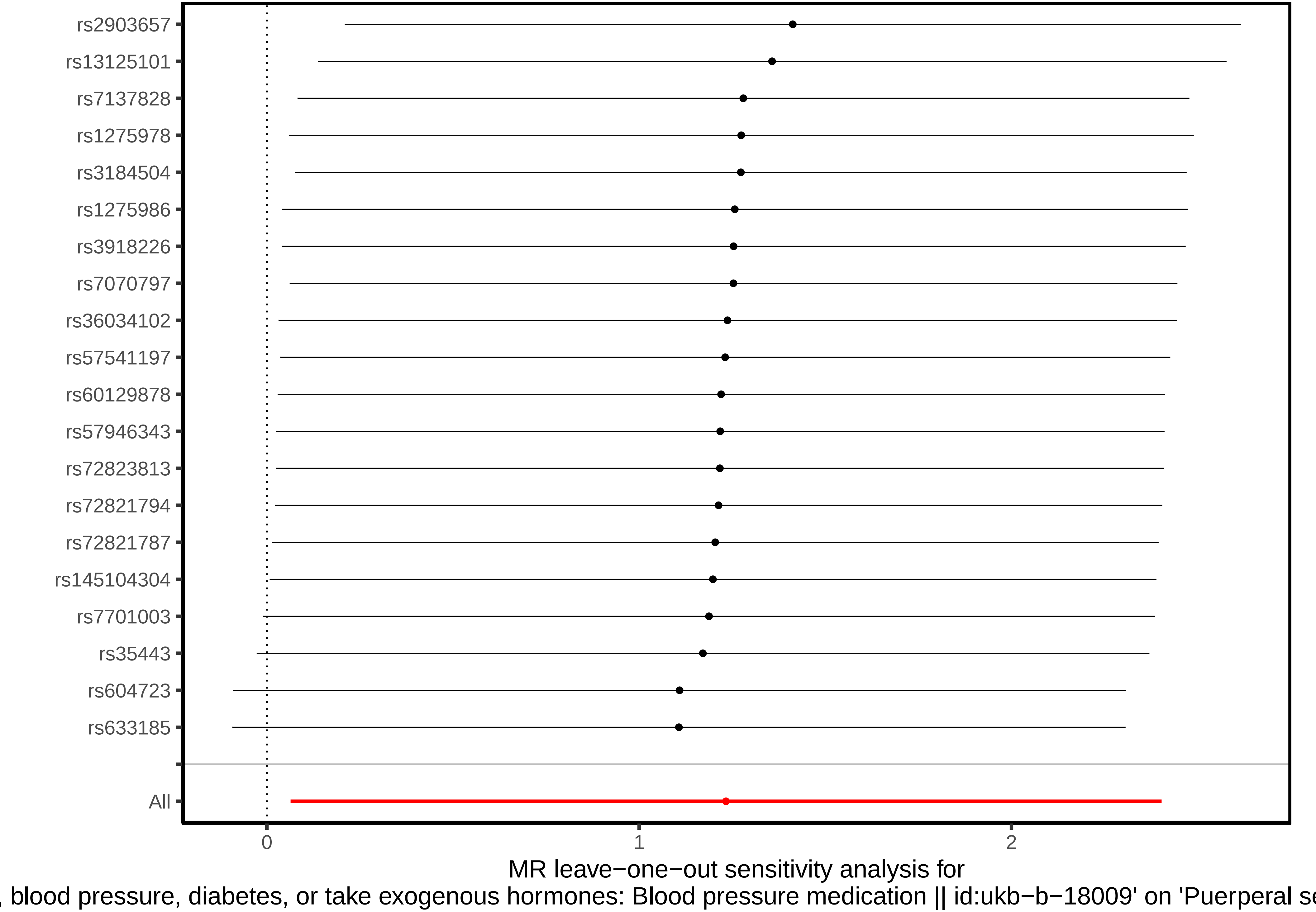
rs2903657
rs13125101
rs7137828
rs1275978
rs3184504
rs1275986
rs3918226
rs7070797
rs36034102
rs57541197
rs60129878
rs57946343
rs72823813
rs72821794
rs72821787
rs145104304
rs7701003
rs35443
rs604723
rs633185
All
0
1
2
MR leave−one−out sensitivity analysis for
, blood pressure, diabetes, or take exogenous hormones: Blood pressure medication || id:ukb−b−18009' on 'Puerperal se

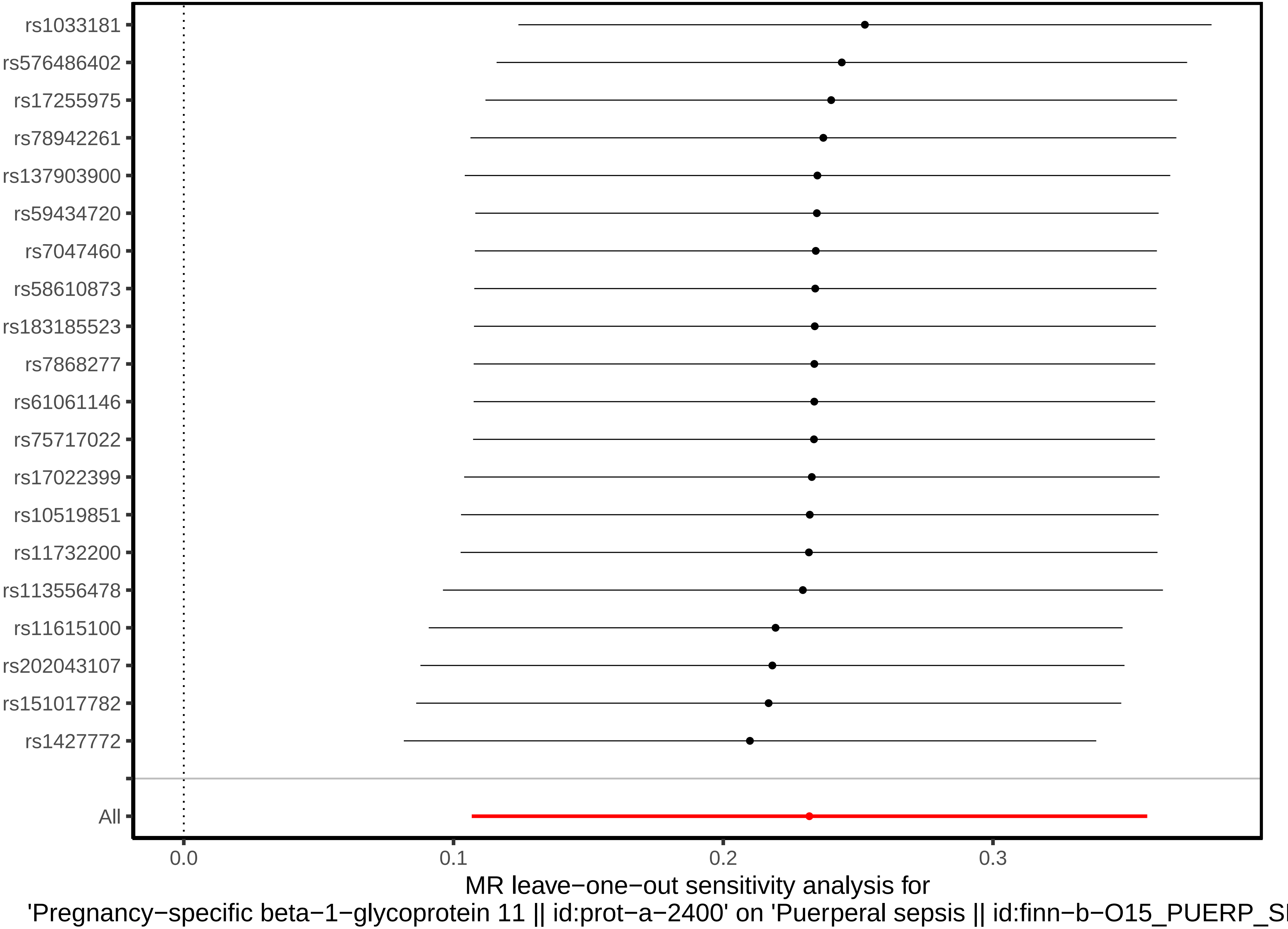

rs1033181
rs576486402
rs17255975
rs78942261
rs137903900
rs59434720
rs7047460
rs58610873
rs183185523
rs7868277
rs61061146
rs75717022
rs17022399
rs10519851
rs11732200
rs113556478
rs11615100
rs202043107
rs151017782
rs1427772
All
0.0
0.1
0.2
0.3
MR leave−one−out sensitivity analysis for
'Pregnancy−specific beta−1−glycoprotein 11 || id:prot−a−2400' on 'Puerperal sepsis || id:finn−b−O15_PUERP_S

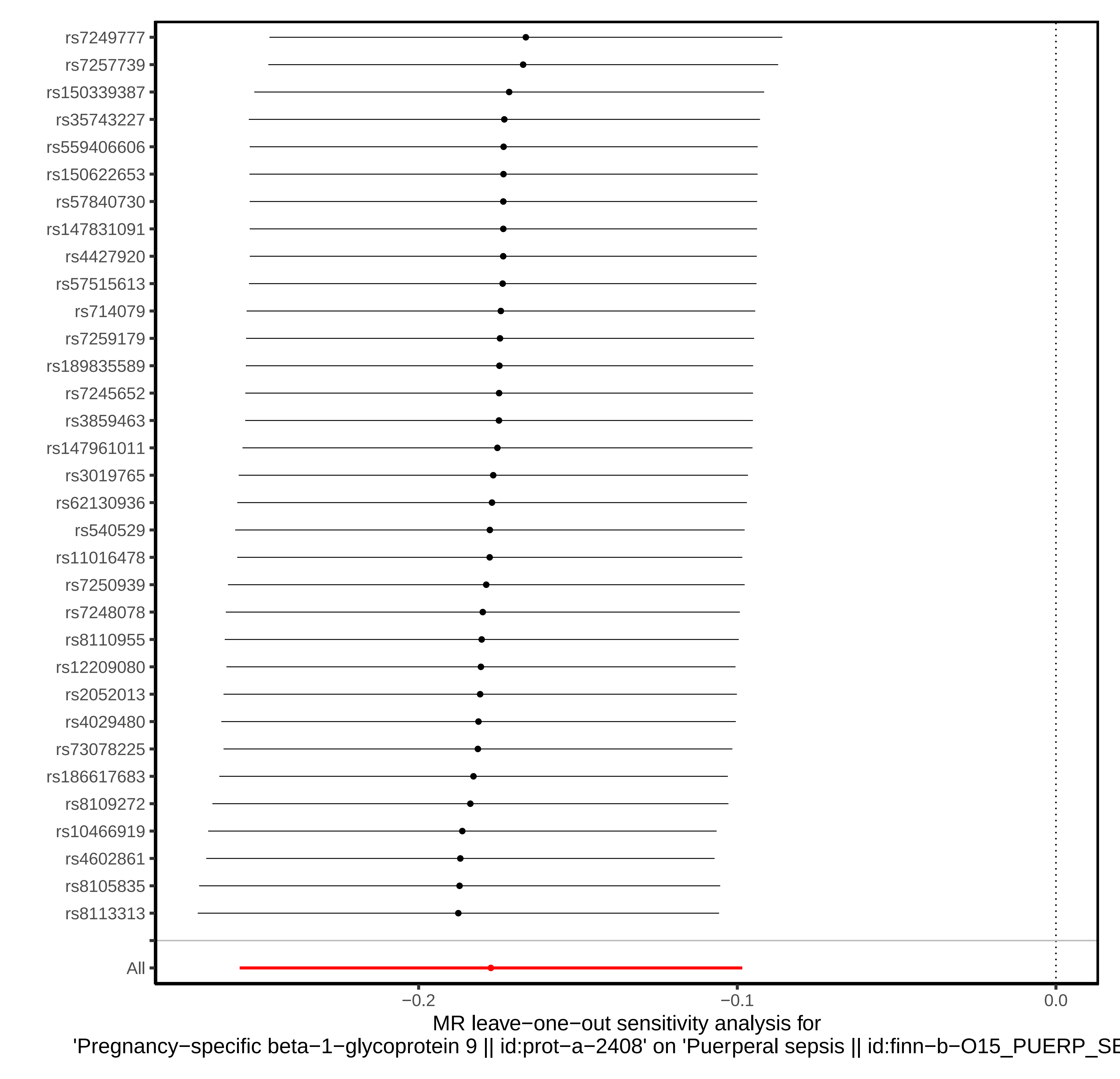
rs7249777
rs7257739
rs150339387
rs35743227
rs559406606
rs150622653
rs57840730
rs147831091
rs4427920
rs57515613
rs714079
rs7259179
rs189835589
rs7245652
rs3859463
rs147961011
rs3019765
rs62130936
rs540529
rs11016478
rs7250939
rs7248078
rs8110955
rs12209080
rs2052013
rs4029480
rs73078225
rs186617683
rs8109272
rs10466919
rs4602861
rs8105835
rs8113313
All
−0.2
−0.1
0.0
MR leave−one−out sensitivity analysis for
'Pregnancy−specific beta−1−glycoprotein 9 || id:prot−a−2408' on 'Puerperal sepsis || id:finn−b−O15_PUERP_SE

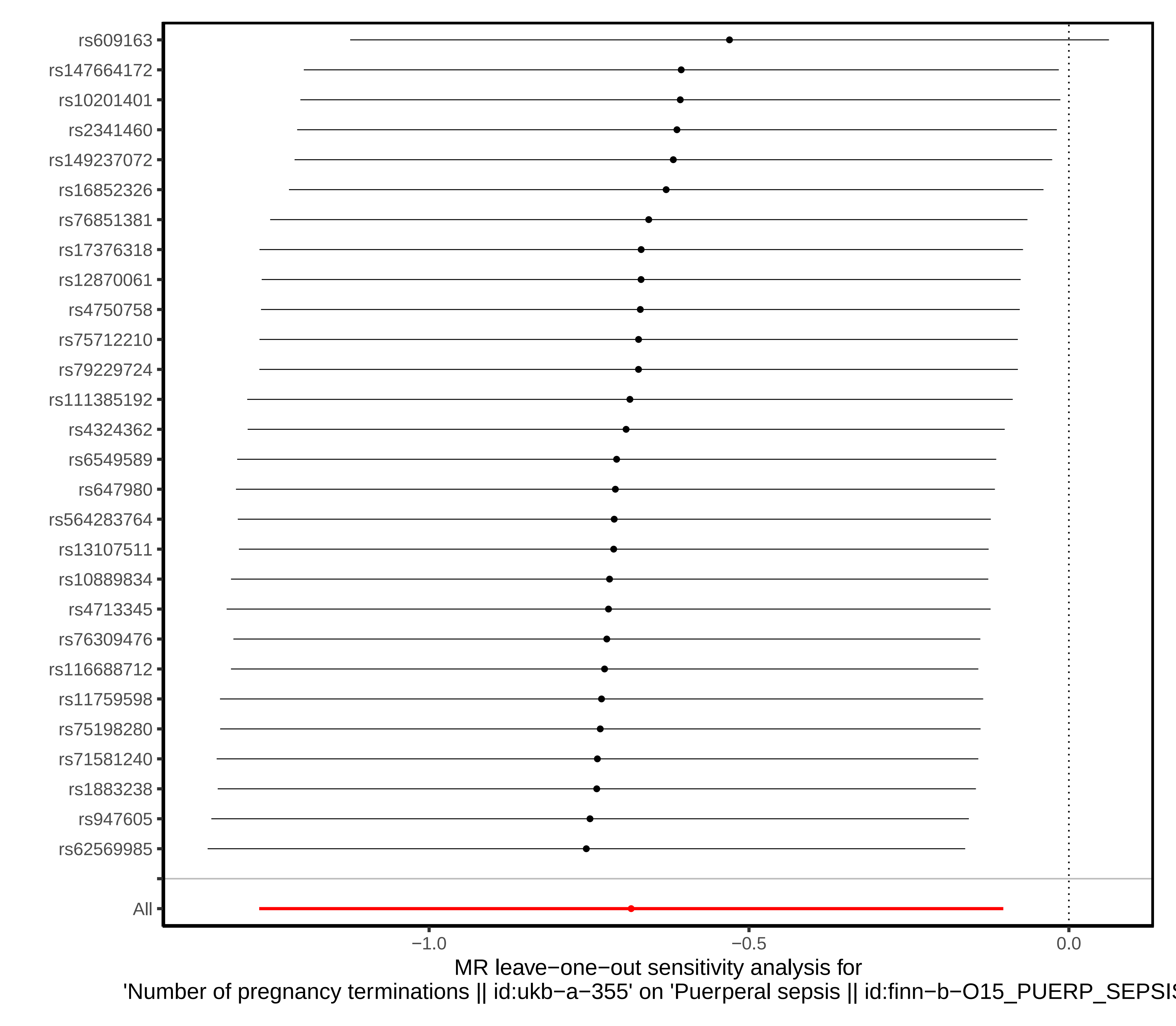
rs609163
rs147664172
rs10201401
rs2341460
rs149237072
rs16852326
rs76851381
rs17376318
rs12870061
rs4750758
rs75712210
rs79229724
rs111385192
rs4324362
rs6549589
rs647980
rs564283764
rs13107511
rs10889834
rs4713345
rs76309476
rs116688712
rs11759598
rs75198280
rs71581240
rs1883238
rs947605
rs62569985
All
−1.0
−0.5
0.0
MR leave−one−out sensitivity analysis for
'Number of pregnancy terminations || id:ukb−a−355' on 'Puerperal sepsis || id:finn−b−O15_PUERP_SEPSIS
