# Supplementary material for: Global burden, projections, and causal factors of maternal sepsis and other maternal infections: A comprehensive epidemiological and mendelian randomization study
Source: PLoS Negl Trop Dis. 2026 May 27;20(5):e0014374. doi: 10.1371/journal.pntd.0014374 (PMC13229374; doi:10.1371/journal.pntd.0014374)
Supplement: S6 Fig — The consistency of results remains robust even after excluding individual genetic variants in each analysis, indicating a high level of reliability and stability in our findings. (PDF) [file pntd.0014374.s006.pdf]

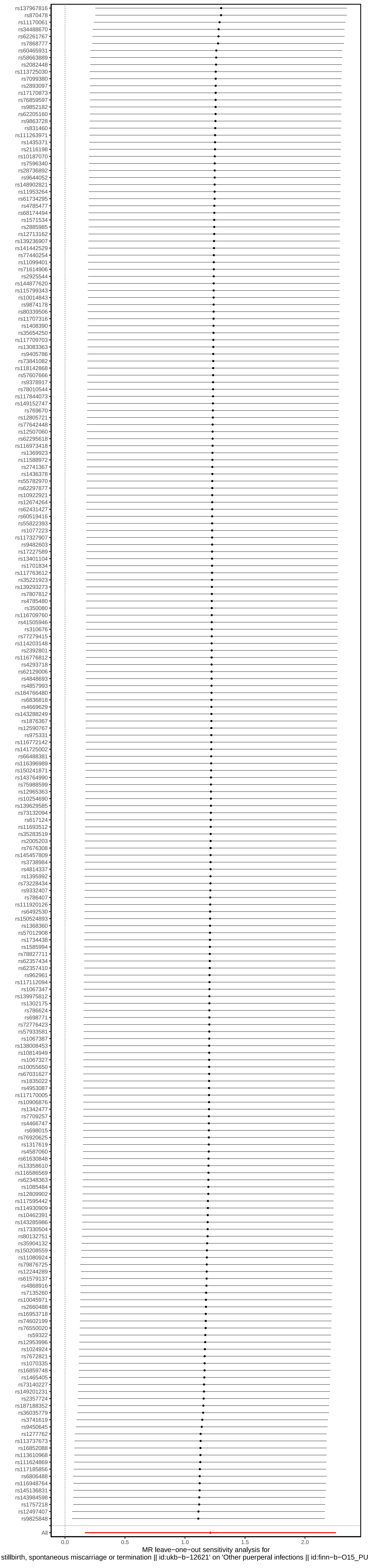
rs137967816
rs870478
rs11170061
rs34488670
rs62261767
rs7868777
rs60465931
rs58663889
rs2082448
rs113725030
rs7099380
rs2893097
rs17170873
rs76859597
rs9852182
rs62205160
rs9863728
rs831460
rs111263971
rs1435371
rs2116198
rs10187070
rs7596340
rs28736892
rs9644052
rs148902821
rs11953264
rs61734295
rs4785477
rs68174494
rs1571534
rs2885985
rs12713162
rs139236907
rs141442529
rs77440254
rs11099401
rs71614906
rs2925544
rs144877620
rs115799343
rs10014843
rs9874178
rs80339506
rs11707316
rs1408390
rs35654250
rs117709703
rs13083363
rs9405786
rs73841082
rs118142868
rs57607666
rs9378917
rs78010544
rs117844073
rs149152747
rs769670
rs12805721
rs77642448
rs12507060
rs62295618
rs116973418
rs1369923
rs11588972
rs2741367
rs1436378
rs55782970
rs62297877
rs10922921
rs12674264
rs62431427
rs60519416
rs55822393
rs1077223
rs117327907
rs9482603
rs17227589
rs13401104
rs1701834
rs117763612
rs35221923
rs139293273
rs7807812
rs4785480
rs350080
rs116709760
rs41505946
rs310676
rs77279415
rs114203148
rs2392801
rs116776812
rs4293718
rs62129006
rs4848693
rs4857993
rs184766480
rs6836818
rs4669629
rs143288249
rs1876367
rs12590767
rs975331
rs116772142
rs141725002
rs66488381
rs116396989
rs150241871
rs143764990
rs75988599
rs12965363
rs10254690
rs139629585
rs73132094
rs617124
rs11693512
rs35283519
rs2005203
rs7676308
rs145457809
rs3738984
rs4814337
rs1395992
rs73228434
rs9332407
rs786407
rs111920126
rs6492530
rs150524893
rs1368360
rs57012908
rs1734438
rs1585994
rs78827711
rs62357434
rs62357410
rs962961
rs117112094
rs1067347
rs139975812
rs1302175
rs786624
rs698771
rs72776423
rs57933581
rs1067387
rs138008453
rs10814949
rs1067327
rs10055650
rs67031627
rs1835022
rs4953087
rs117170005
rs10906876
rs1342477
rs7709257
rs4466747
rs698015
rs76920625
rs1317619
rs4587060
rs61630848
rs13358610
rs116586569
rs62348363
rs1085484
rs12809902
rs117595442
rs114930909
rs10462391
rs143285986
rs17330504
rs80132751
rs35904132
rs150208559
rs11080924
rs79876725
rs12244289
rs61579137
rs4868916
rs7135260
rs10045971
rs2660488
rs16953718
rs74602199
rs76550020
rs59322
rs12953996
rs1024924
rs7672821
rs1070335
rs16859748
rs1465405
rs73140227
rs149201231
rs2357724
rs187188352
rs36035779
rs3741619
rs9450645
rs1277762
rs113737673
rs16852088
rs113610968
rs111624869
rs117185856
rs6806488
rs116948764
rs145136831
rs143984598
rs1757218
rs12497407
rs9825848
All
0.0
0.5
1.0
1.5
2.0
MR leave−one−out sensitivity analysis for
stillbirth, spontaneous miscarriage or termination || id:ukb−b−12621' on 'Other puerperal infections || id:finn−b−O15_PU

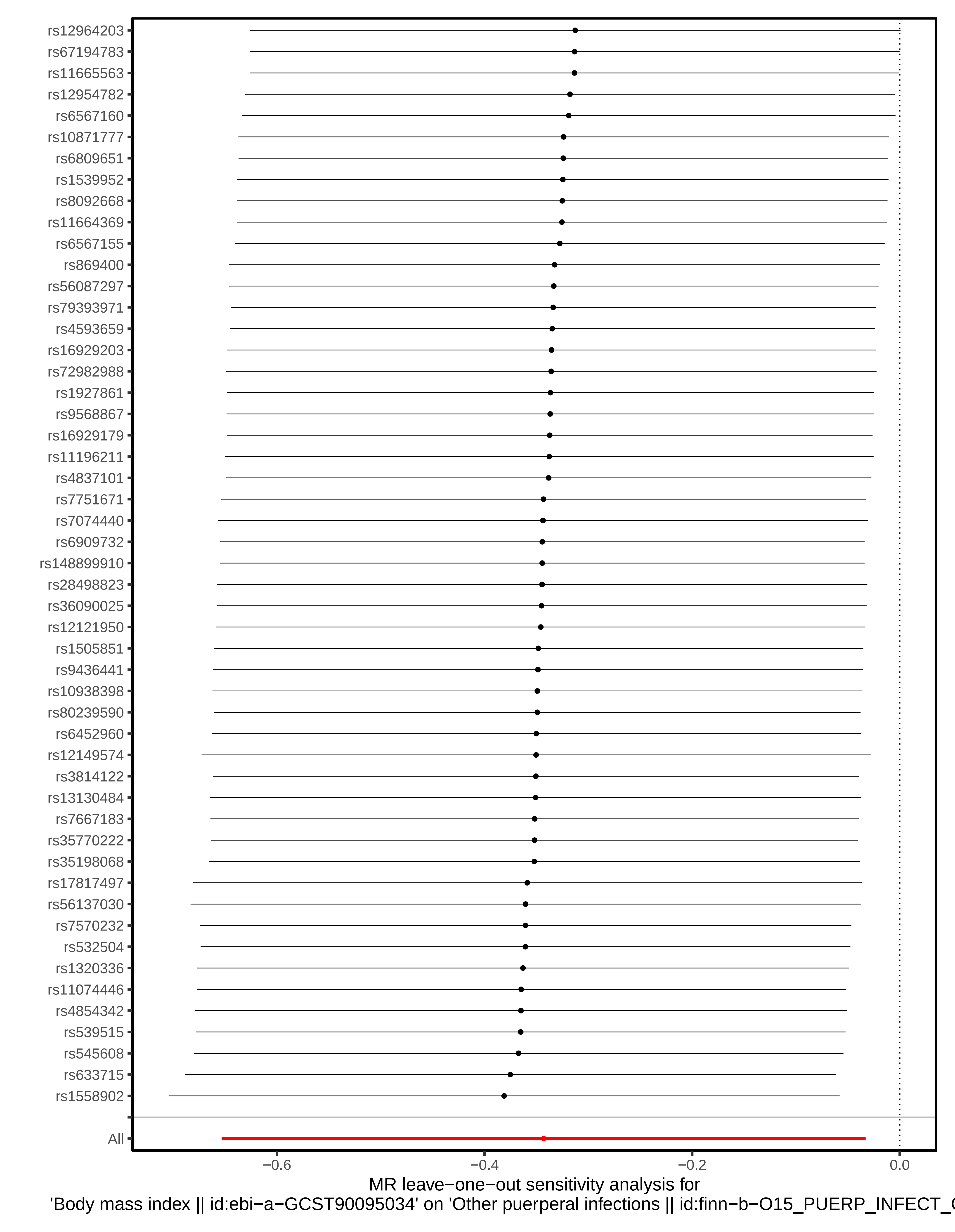

rs12964203
rs67194783
rs11665563
rs12954782
rs6567160
rs10871777
rs6809651
rs1539952
rs8092668
rs11664369
rs6567155
rs869400
rs56087297
rs79393971
rs4593659
rs16929203
rs72982988
rs1927861
rs9568867
rs16929179
rs11196211
rs4837101
rs7751671
rs7074440
rs6909732
rs148899910
rs28498823
rs36090025
rs12121950
rs1505851
rs9436441
rs10938398
rs80239590
rs6452960
rs12149574
rs3814122
rs13130484
rs7667183
rs35770222
rs35198068
rs17817497
rs56137030
rs7570232
rs532504
rs1320336
rs11074446
rs4854342
rs539515
rs545608
rs633715
rs1558902
All
−0.6
−0.4
−0.2
0.0
MR leave−one−out sensitivity analysis for
'Body mass index || id:ebi−a−GCST90095034' on 'Other puerperal infections || id:finn−b−O15_PUERP_INFECT_

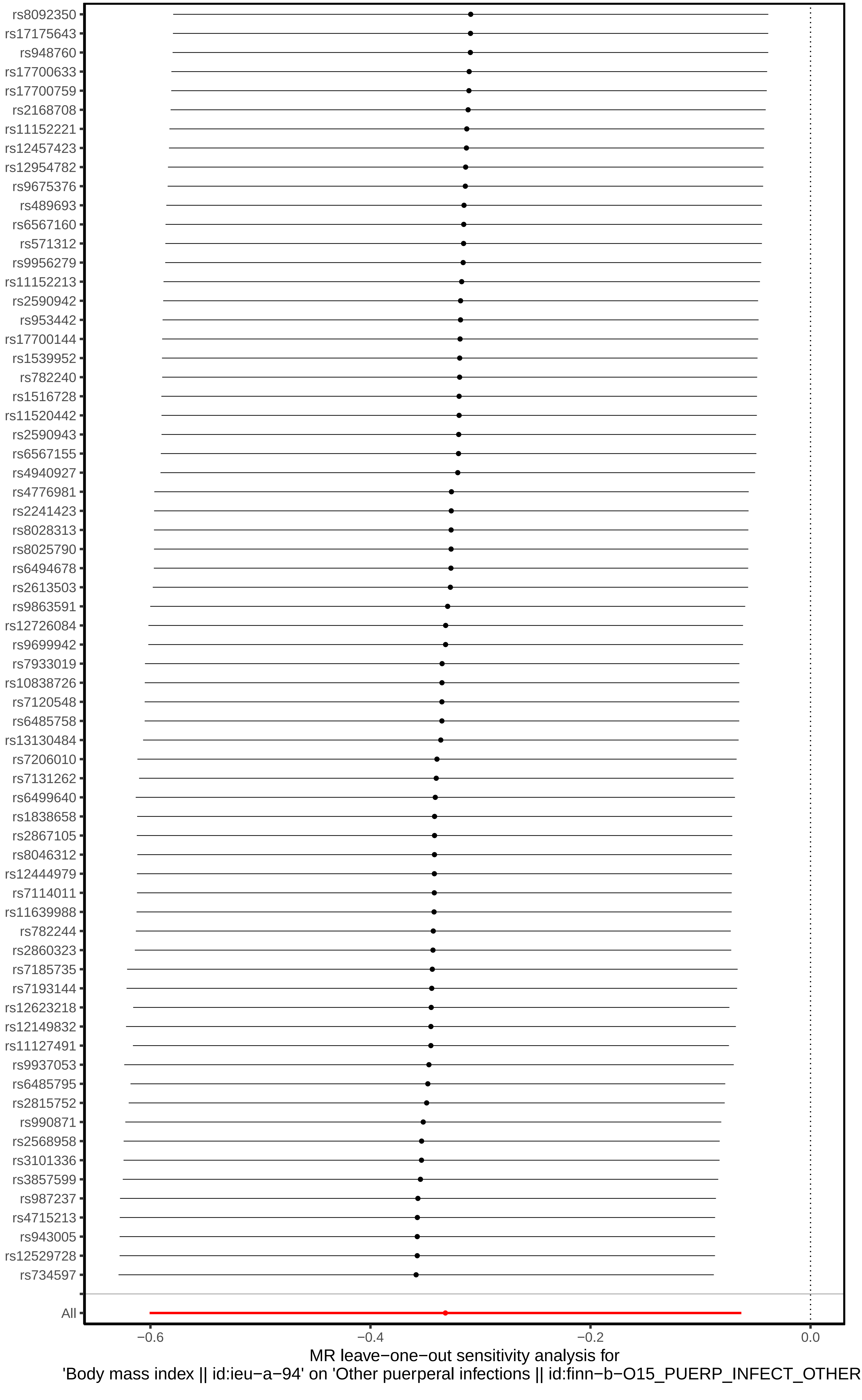

rs8092350
rs17175643
rs948760
rs17700633
rs17700759
rs2168708
rs11152221
rs12457423
rs12954782
rs9675376
rs489693
rs6567160
rs571312
rs9956279
rs11152213
rs2590942
rs953442
rs17700144
rs1539952
rs782240
rs1516728
rs11520442
rs2590943
rs6567155
rs4940927
rs4776981
rs2241423
rs8028313
rs8025790
rs6494678
rs2613503
rs9863591
rs12726084
rs9699942
rs7933019
rs10838726
rs7120548
rs6485758
rs13130484
rs7206010
rs7131262
rs6499640
rs1838658
rs2867105
rs8046312
rs12444979
rs7114011
rs11639988
rs782244
rs2860323
rs7185735
rs7193144
rs12623218
rs12149832
rs11127491
rs9937053
rs6485795
rs2815752
rs990871
rs2568958
rs3101336
rs3857599
rs987237
rs4715213
rs943005
rs12529728
rs734597
All
−0.6
−0.4
−0.2
0.0
MR leave−one−out sensitivity analysis for
'Body mass index || id:ieu−a−94' on 'Other puerperal infections || id:finn−b−O15_PUERP_INFECT_OTHER

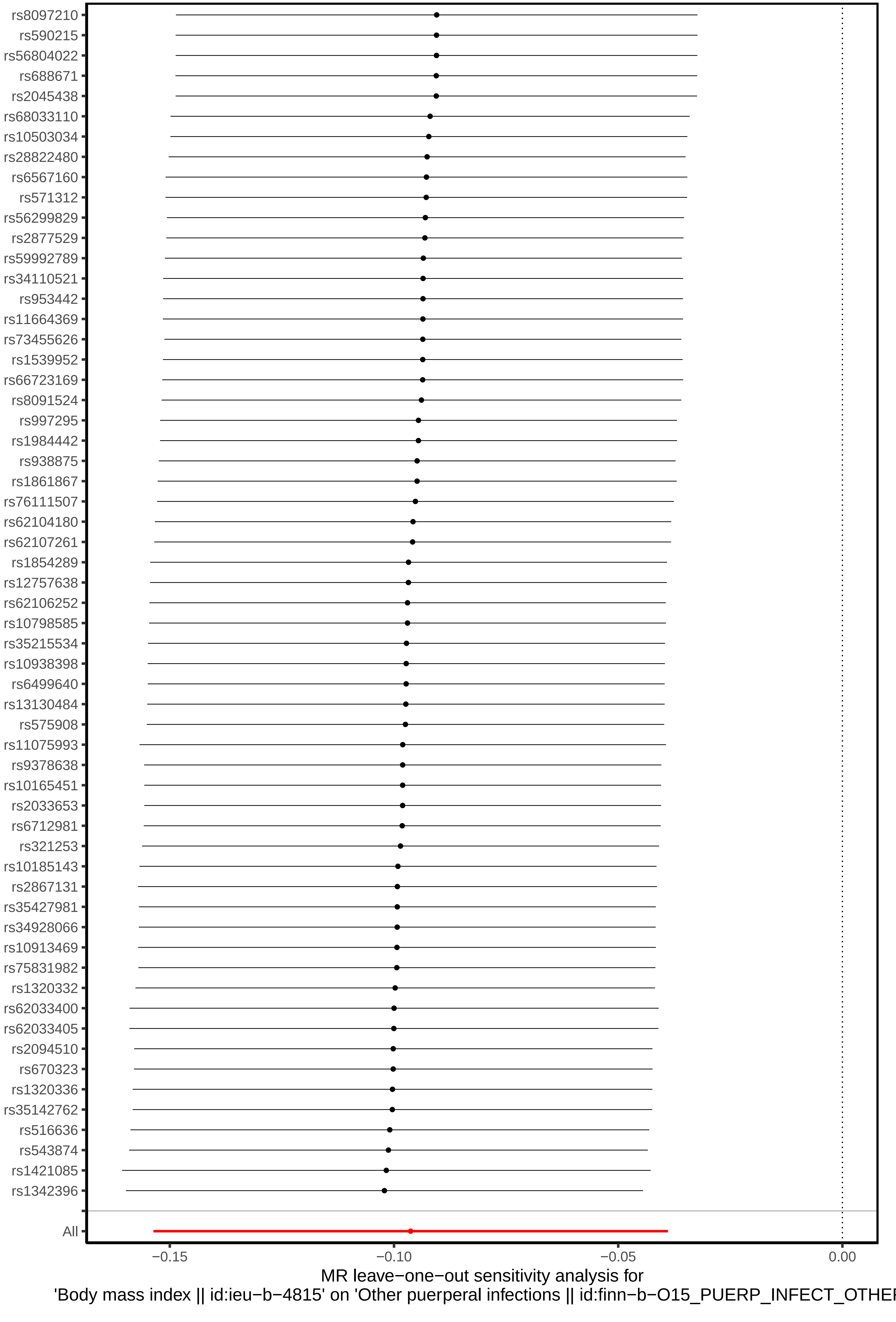
rs8097210
rs590215
rs56804022
rs688671
rs2045438
rs68033110
rs10503034
rs28822480
rs6567160
rs571312
rs56299829
rs2877529
rs59992789
rs34110521
rs953442
rs11664369
rs73455626
rs1539952
rs66723169
rs8091524
rs997295
rs1984442
rs938875
rs1861867
rs76111507
rs62104180
rs62107261
rs1854289
rs12757638
rs62106252
rs10798585
rs35215534
rs10938398
rs6499640
rs13130484
rs575908
rs11075993
rs9378638
rs10165451
rs2033653
rs6712981
rs321253
rs10185143
rs2867131
rs35427981
rs34928066
rs10913469
rs75831982
rs1320332
rs62033400
rs62033405
rs2094510
rs670323
rs1320336
rs35142762
rs516636
rs543874
rs1421085
rs1342396
All
−0.15
−0.10
−0.05
0.00
MR leave−one−out sensitivity analysis for
'Body mass index || id:ieu−b−4815' on 'Other puerperal infections || id:finn−b−O15_PUERP_INFECT_OTHEF

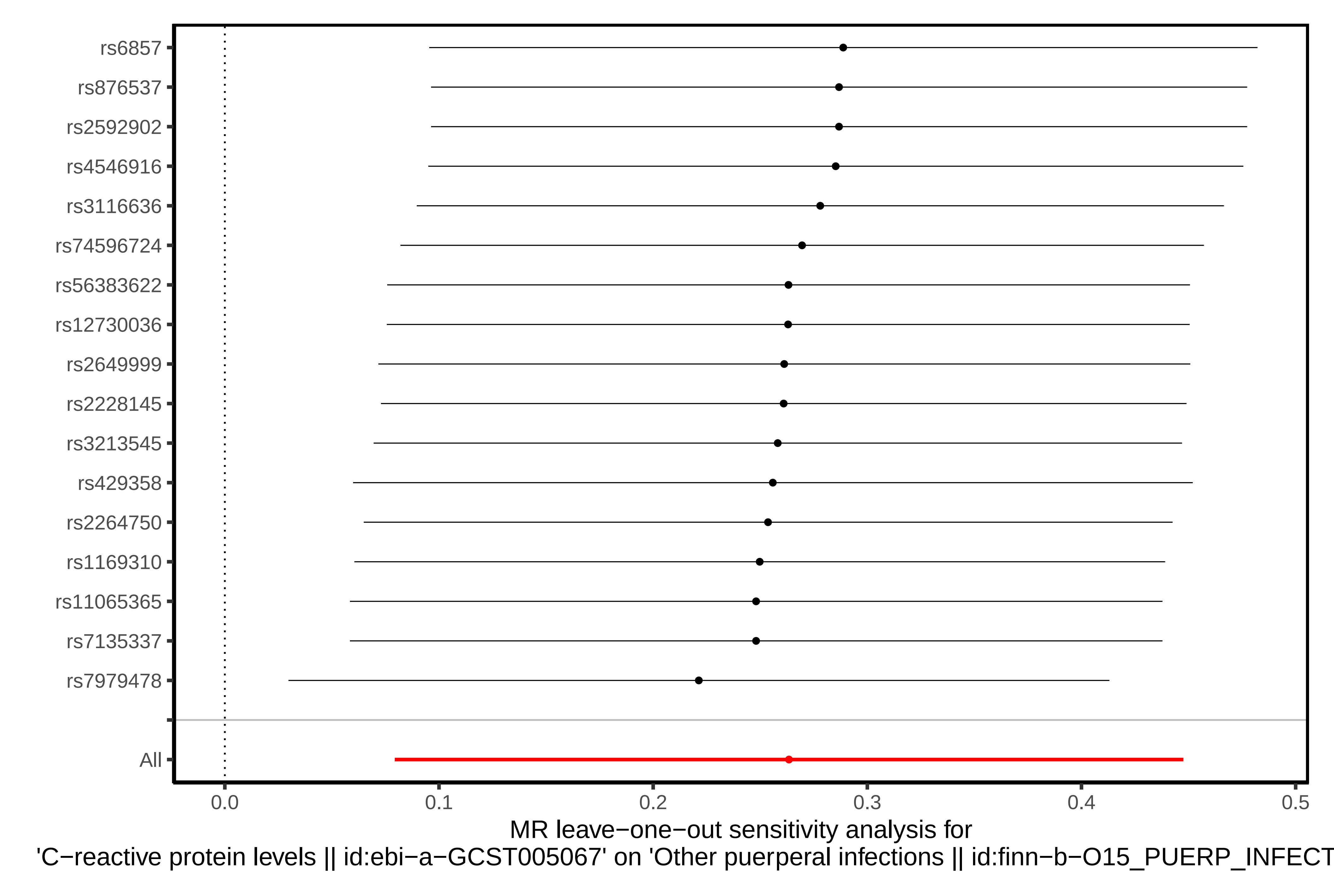
rs6857
rs876537
rs2592902
rs4546916
rs3116636
rs74596724
rs56383622
rs12730036
rs2649999
rs2228145
rs3213545
rs429358
rs2264750
rs1169310
rs11065365
rs7135337
rs7979478
All
0.0
0.1
0.2
0.3
0.4
0.5
MR leave−one−out sensitivity analysis for
'C−reactive protein levels || id:ebi−a−GCST005067' on 'Other puerperal infections || id:finn−b−O15_PUERP_INFECT

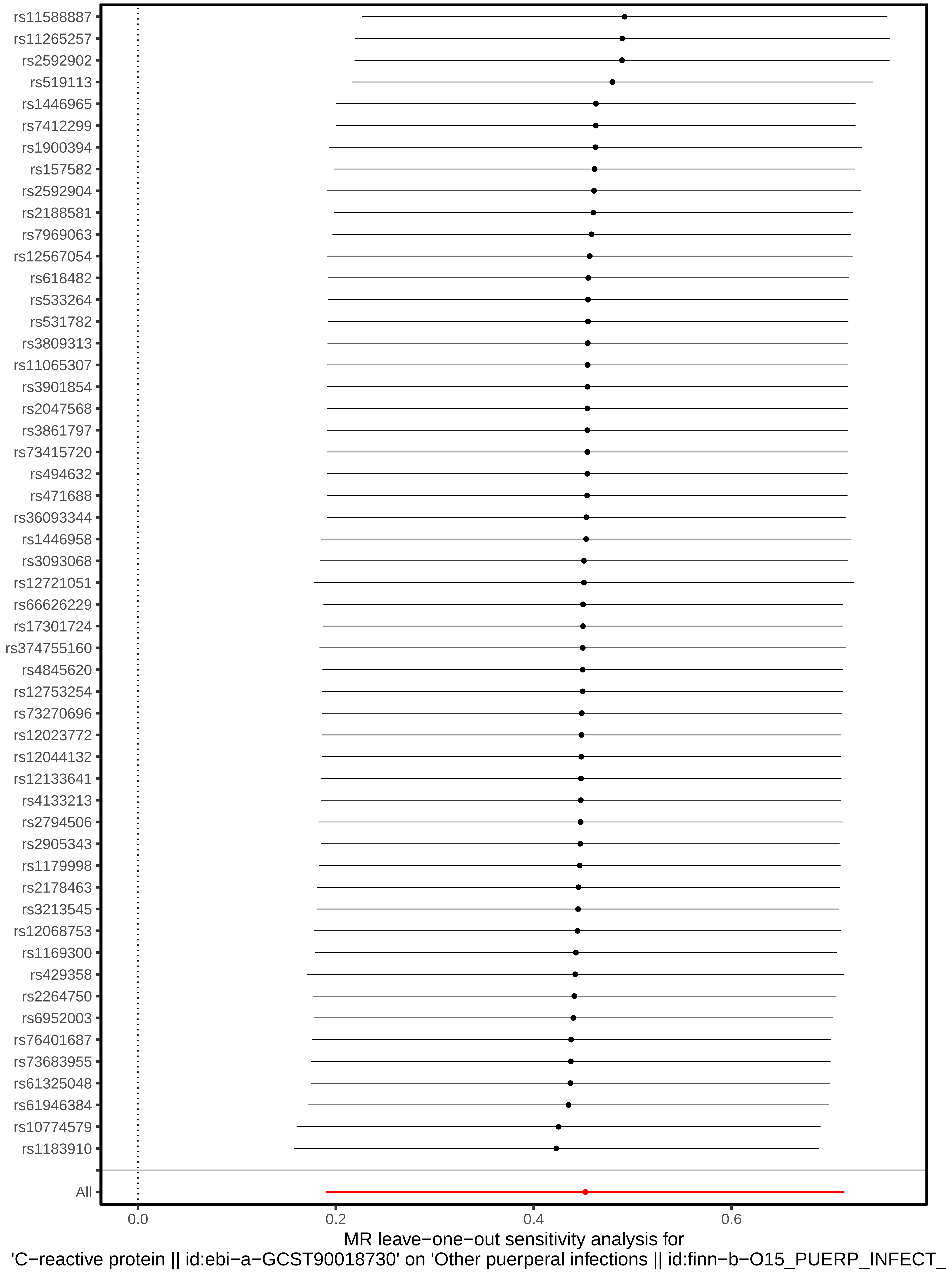
rs11588887
rs11265257
rs2592902
rs519113
rs1446965
rs7412299
rs1900394
rs157582
rs2592904
rs2188581
rs7969063
rs12567054
rs618482
rs533264
rs531782
rs3809313
rs11065307
rs3901854
rs2047568
rs3861797
rs73415720
rs494632
rs471688
rs36093344
rs1446958
rs3093068
rs12721051
rs66626229
rs17301724
rs374755160
rs4845620
rs12753254
rs73270696
rs12023772
rs12044132
rs12133641
rs4133213
rs2794506
rs2905343
rs1179998
rs2178463
rs3213545
rs12068753
rs1169300
rs429358
rs2264750
rs6952003
rs76401687
rs73683955
rs61325048
rs61946384
rs10774579
rs1183910
All
0.0
0.2
0.4
0.6
MR leave−one−out sensitivity analysis for
'C−reactive protein || id:ebi−a−GCST90018730' on 'Other puerperal infections || id:finn−b−O15_PUERP_INFECT_

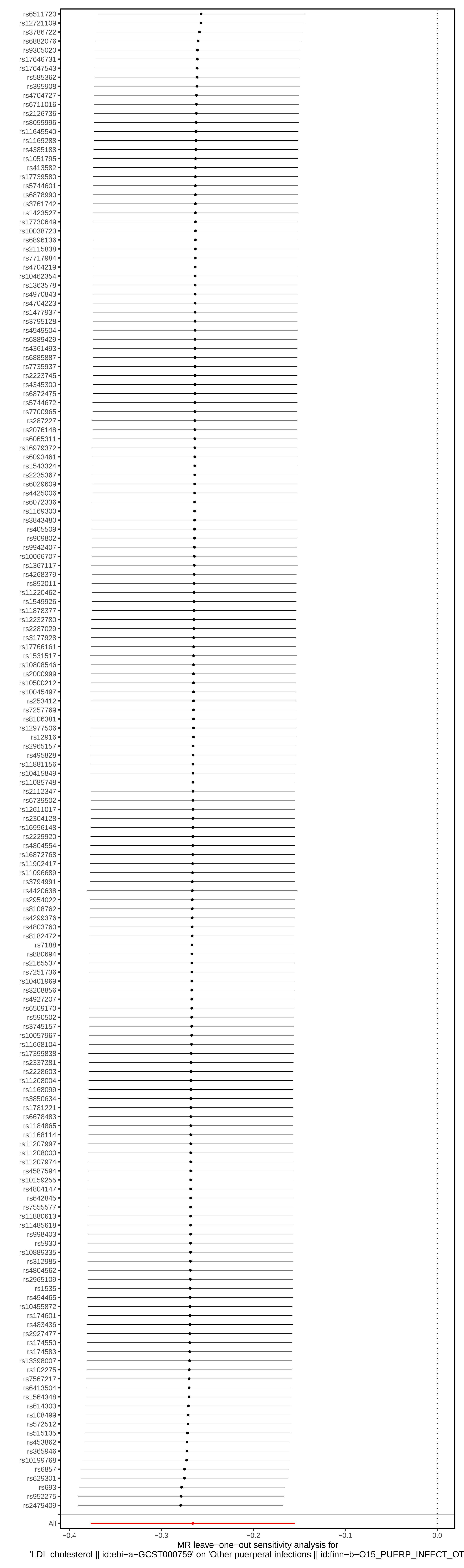
rs6511720
rs12721109
rs3786722
rs6882076
rs9305020
rs17646731
rs17647543
rs585362
rs395908
rs4704727
rs6711016
rs2126736
rs8099996
rs11645540
rs1169288
rs4385188
rs1051795
rs413582
rs17739580
rs5744601
rs6878990
rs3761742
rs1423527
rs17730649
rs10038723
rs6896136
rs2115838
rs7717984
rs4704219
rs10462354
rs1363578
rs4970843
rs4704223
rs1477937
rs3795128
rs4549504
rs6889429
rs4361493
rs6885887
rs7735937
rs2223745
rs4345300
rs6872475
rs5744672
rs7700965
rs287227
rs2076148
rs6065311
rs16979372
rs6093461
rs1543324
rs2235367
rs6029609
rs4425006
rs6072336
rs1169300
rs3843480
rs405509
rs909802
rs9942407
rs10066707
rs1367117
rs4268379
rs892011
rs11220462
rs1549926
rs11878377
rs12232780
rs2287029
rs3177928
rs17766161
rs1531517
rs10808546
rs2000999
rs10500212
rs10045497
rs253412
rs7257769
rs8106381
rs12977506
rs12916
rs2965157
rs495828
rs11881156
rs10415849
rs11085748
rs2112347
rs6739502
rs12611017
rs2304128
rs16996148
rs2229920
rs4804554
rs16872768
rs11902417
rs11096689
rs3794991
rs4420638
rs2954022
rs8108762
rs4299376
rs4803760
rs8182472
rs7188
rs880694
rs2165537
rs7251736
rs10401969
rs3208856
rs4927207
rs6509170
rs590502
rs3745157
rs10057967
rs11668104
rs17399838
rs2337381
rs2228603
rs11208004
rs1168099
rs3850634
rs1781221
rs6678483
rs1184865
rs1168114
rs11207997
rs11208000
rs11207974
rs4587594
rs10159255
rs4804147
rs642845
rs7555577
rs11880613
rs11485618
rs998403
rs5930
rs10889335
rs312985
rs4804562
rs2965109
rs1535
rs494465
rs10455872
rs174601
rs483436
rs2927477
rs174550
rs174583
rs13398007
rs102275
rs7567217
rs6413504
rs1564348
rs614303
rs108499
rs572512
rs515135
rs453862
rs365946
rs10199768
rs6857
rs629301
rs693
rs952275
rs2479409
All
−0.4
−0.3
−0.2
−0.1
0.0
MR leave−one−out sensitivity analysis for
'LDL cholesterol || id:ebi−a−GCST000759' on 'Other puerperal infections || id:finn−b−O15_PUERP_INFECT_OT

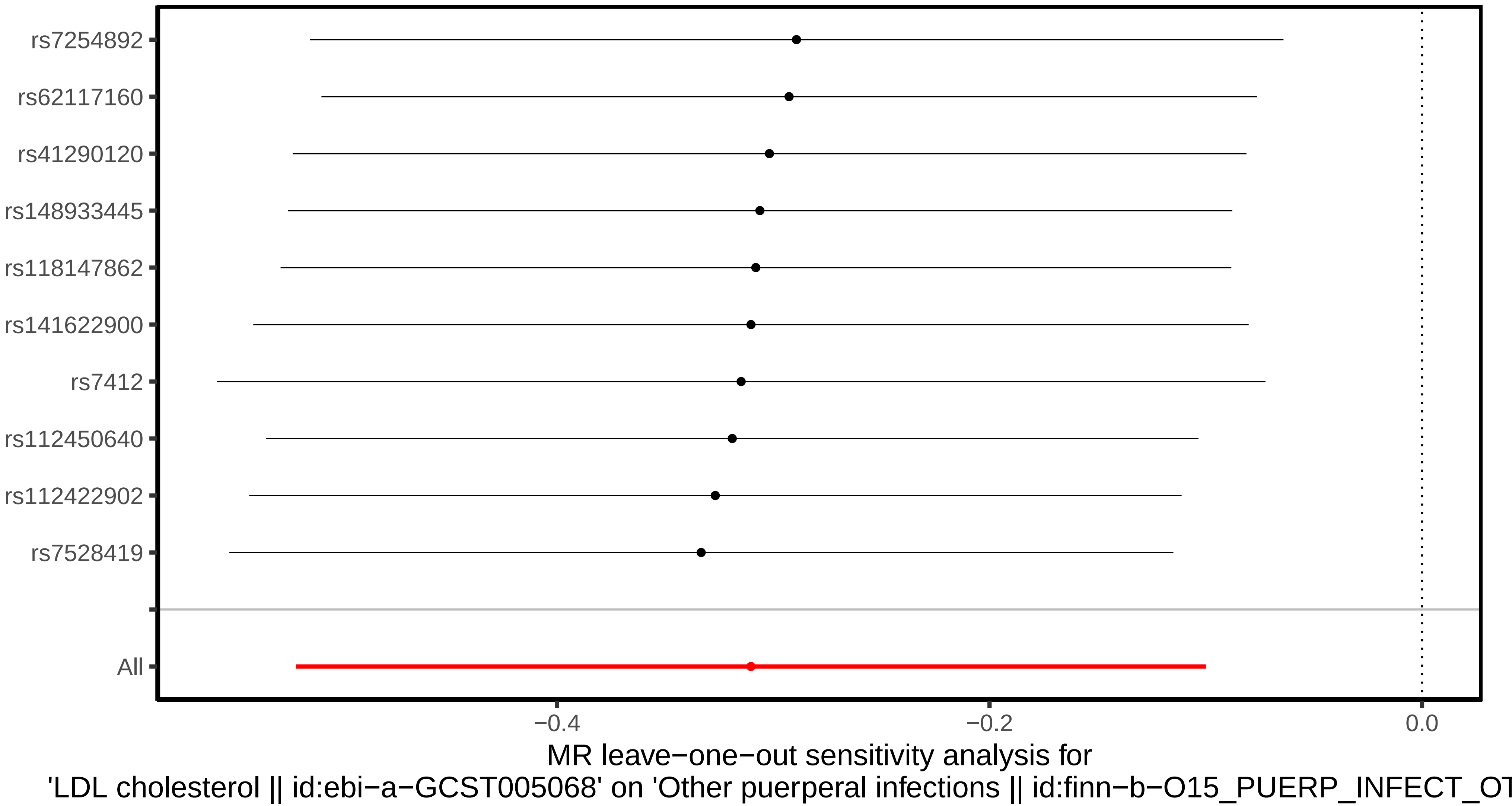
rs7254892
rs62117160
rs41290120
rs148933445
rs118147862
rs141622900
rs7412
rs112450640
rs112422902
rs7528419
All
−0.4
−0.2
0.0
MR leave−one−out sensitivity analysis for
'LDL cholesterol || id:ebi−a−GCST005068' on 'Other puerperal infections || id:finn−b−O15_PUERP_INFECT_OT

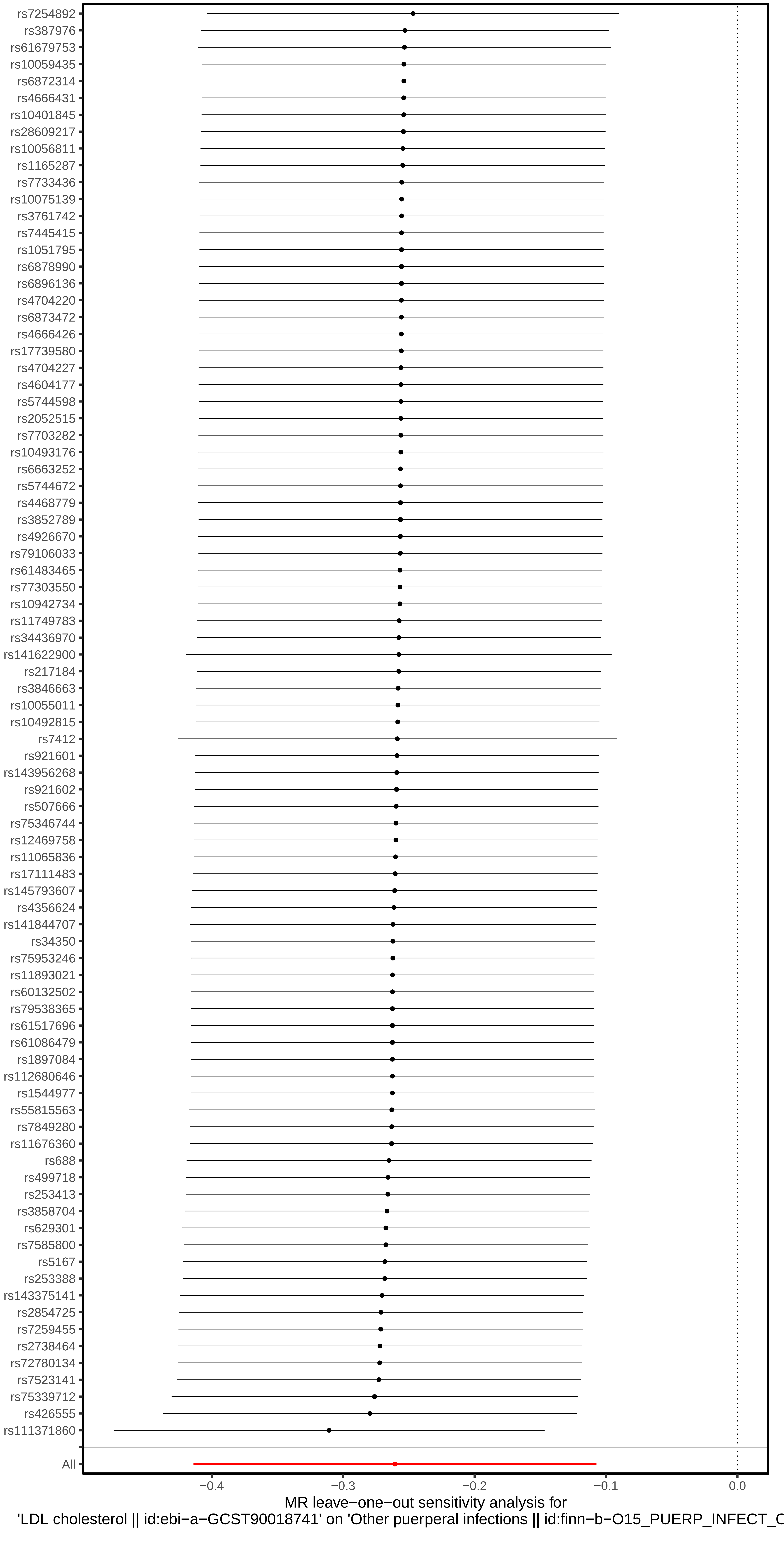
rs7254892
rs387976
rs61679753
rs10059435
rs6872314
rs4666431
rs10401845
rs28609217
rs10056811
rs1165287
rs7733436
rs10075139
rs3761742
rs7445415
rs1051795
rs6878990
rs6896136
rs4704220
rs6873472
rs4666426
rs17739580
rs4704227
rs4604177
rs5744598
rs2052515
rs7703282
rs10493176
rs6663252
rs5744672
rs4468779
rs3852789
rs4926670
rs79106033
rs61483465
rs77303550
rs10942734
rs11749783
rs34436970
rs141622900
rs217184
rs3846663
rs10055011
rs10492815
rs7412
rs921601
rs143956268
rs921602
rs507666
rs75346744
rs12469758
rs11065836
rs17111483
rs145793607
rs4356624
rs141844707
rs34350
rs75953246
rs11893021
rs60132502
rs79538365
rs61517696
rs61086479
rs1897084
rs112680646
rs1544977
rs55815563
rs7849280
rs11676360
rs688
rs499718
rs253413
rs3858704
rs629301
rs7585800
rs5167
rs253388
rs143375141
rs2854725
rs7259455
rs2738464
rs72780134
rs7523141
rs75339712
rs426555
rs111371860
All
−0.4
−0.3
−0.2
−0.1
0.0
MR leave−one−out sensitivity analysis for
'LDL cholesterol || id:ebi−a−GCST90018741' on 'Other puerperal infections || id:finn−b−O15_PUERP_INFECT_C

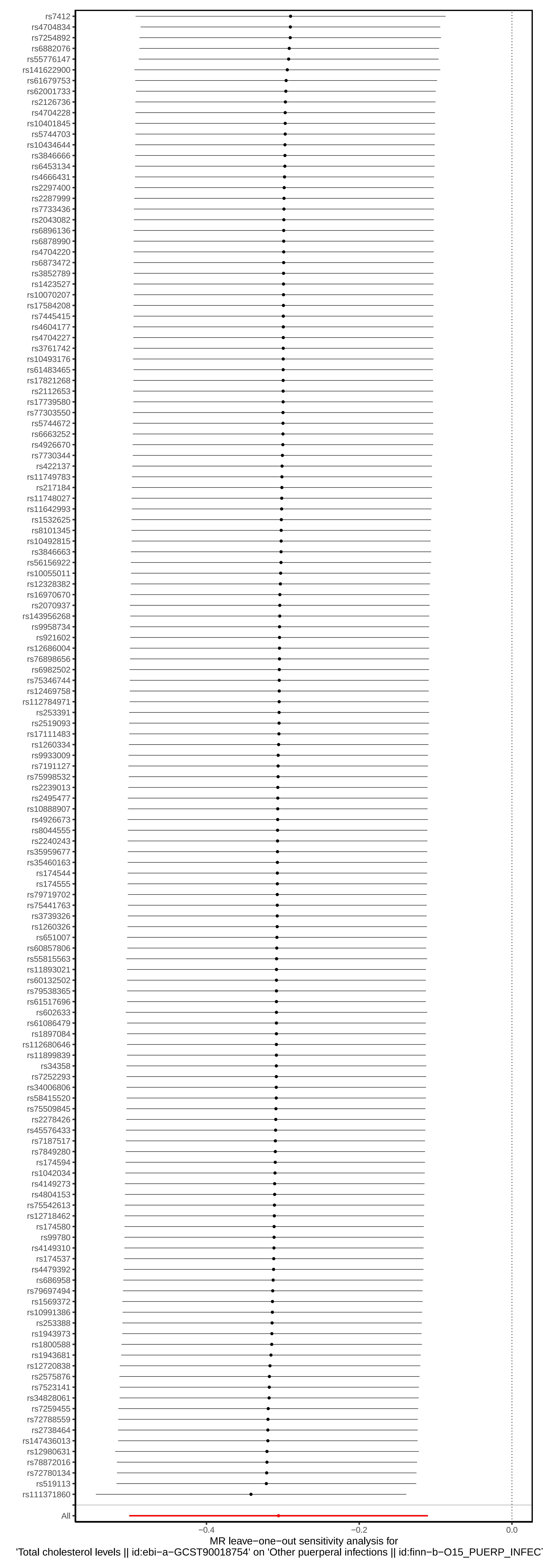
rs7412
rs4704834
rs7254892
rs6882076
rs55776147
rs141622900
rs61679753
rs62001733
rs2126736
rs4704228
rs10401845
rs5744703
rs10434644
rs3846666
rs6453134
rs4666431
rs2297400
rs2287999
rs7733436
rs2043082
rs6896136
rs6878990
rs4704220
rs6873472
rs3852789
rs1423527
rs10070207
rs17584208
rs7445415
rs4604177
rs4704227
rs3761742
rs10493176
rs61483465
rs17821268
rs2112653
rs17739580
rs77303550
rs5744672
rs6663252
rs4926670
rs7730344
rs422137
rs11749783
rs217184
rs11748027
rs11642993
rs1532625
rs8101345
rs10492815
rs3846663
rs56156922
rs10055011
rs12328382
rs16970670
rs2070937
rs143956268
rs9958734
rs921602
rs12686004
rs76898656
rs6982502
rs75346744
rs12469758
rs112784971
rs253391
rs2519093
rs17111483
rs1260334
rs9933009
rs7191127
rs75998532
rs2239013
rs2495477
rs10888907
rs4926673
rs8044555
rs2240243
rs35959677
rs35460163
rs174544
rs174555
rs79719702
rs75441763
rs3739326
rs1260326
rs651007
rs60857806
rs55815563
rs11893021
rs60132502
rs79538365
rs61517696
rs602633
rs61086479
rs1897084
rs112680646
rs11899839
rs34358
rs7252293
rs34006806
rs58415520
rs75509845
rs2278426
rs45576433
rs7187517
rs7849280
rs174594
rs1042034
rs4149273
rs4804153
rs75542613
rs12718462
rs174580
rs99780
rs4149310
rs174537
rs4479392
rs686958
rs79697494
rs1569372
rs10991386
rs253388
rs1943973
rs1800588
rs1943681
rs12720838
rs2575876
rs7523141
rs34828061
rs7259455
rs72788559
rs2738464
rs147436013
rs12980631
rs78872016
rs72780134
rs519113
rs111371860
All
−0.4
−0.2
0.0
MR leave−one−out sensitivity analysis for
'Total cholesterol levels || id:ebi−a−GCST90018754' on 'Other puerperal infections || id:finn−b−O15_PUERP_INFECT

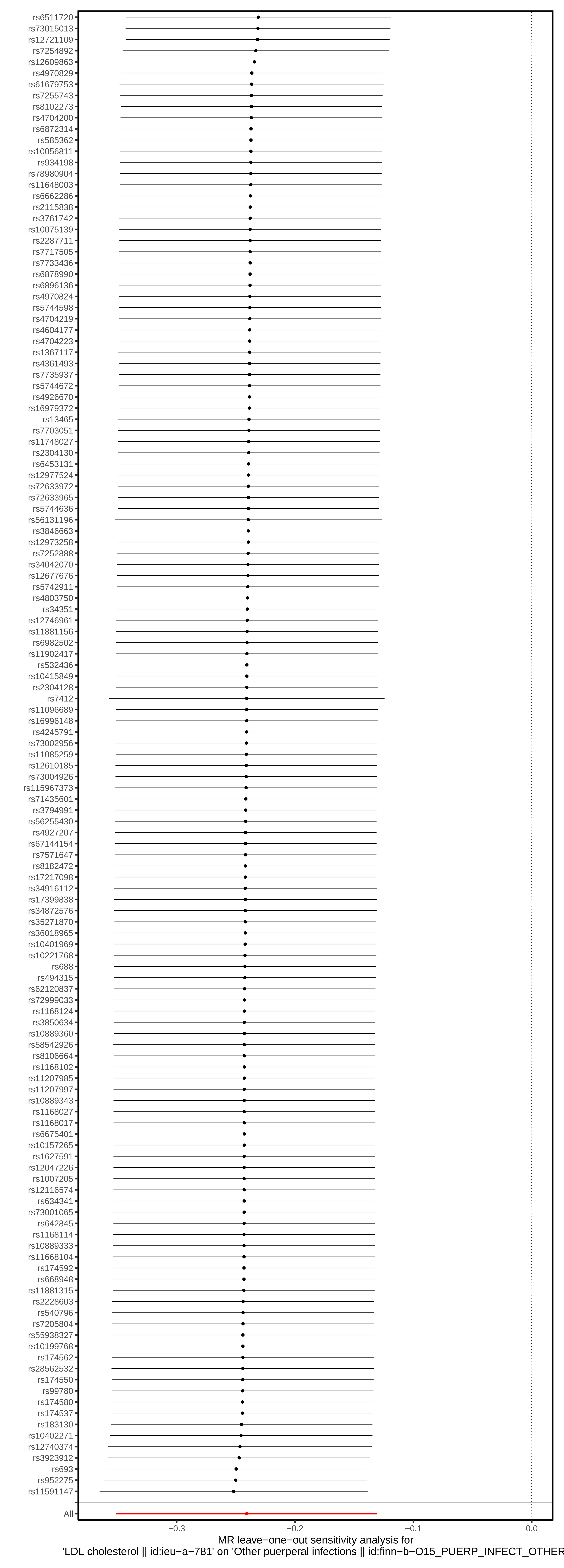
rs6511720
rs73015013
rs12721109
rs7254892
rs12609863
rs4970829
rs61679753
rs7255743
rs8102273
rs4704200
rs6872314
rs585362
rs10056811
rs934198
rs78980904
rs11648003
rs6662286
rs2115838
rs3761742
rs10075139
rs2287711
rs7717505
rs7733436
rs6878990
rs6896136
rs4970824
rs5744598
rs4704219
rs4604177
rs4704223
rs1367117
rs4361493
rs7735937
rs5744672
rs4926670
rs16979372
rs13465
rs7703051
rs11748027
rs2304130
rs6453131
rs12977524
rs72633972
rs72633965
rs5744636
rs56131196
rs3846663
rs12973258
rs7252888
rs34042070
rs12677676
rs5742911
rs4803750
rs34351
rs12746961
rs11881156
rs6982502
rs11902417
rs532436
rs10415849
rs2304128
rs7412
rs11096689
rs16996148
rs4245791
rs73002956
rs11085259
rs12610185
rs73004926
rs115967373
rs71435601
rs3794991
rs56255430
rs4927207
rs67144154
rs7571647
rs8182472
rs17217098
rs34916112
rs17399838
rs34872576
rs35271870
rs36018965
rs10401969
rs10221768
rs688
rs494315
rs62120837
rs72999033
rs1168124
rs3850634
rs10889360
rs58542926
rs8106664
rs1168102
rs11207985
rs11207997
rs10889343
rs1168027
rs1168017
rs6675401
rs10157265
rs1627591
rs12047226
rs1007205
rs12116574
rs634341
rs73001065
rs642845
rs1168114
rs10889333
rs11668104
rs174592
rs668948
rs11881315
rs2228603
rs540796
rs7205804
rs55938327
rs10199768
rs174562
rs28562532
rs174550
rs99780
rs174580
rs174537
rs183130
rs10402271
rs12740374
rs3923912
rs693
rs952275
rs11591147
All
−0.3
−0.2
−0.1
0.0
MR leave−one−out sensitivity analysis for
'LDL cholesterol || id:ieu−a−781' on 'Other puerperal infections || id:finn−b−O15_PUERP_INFECT_OTHER

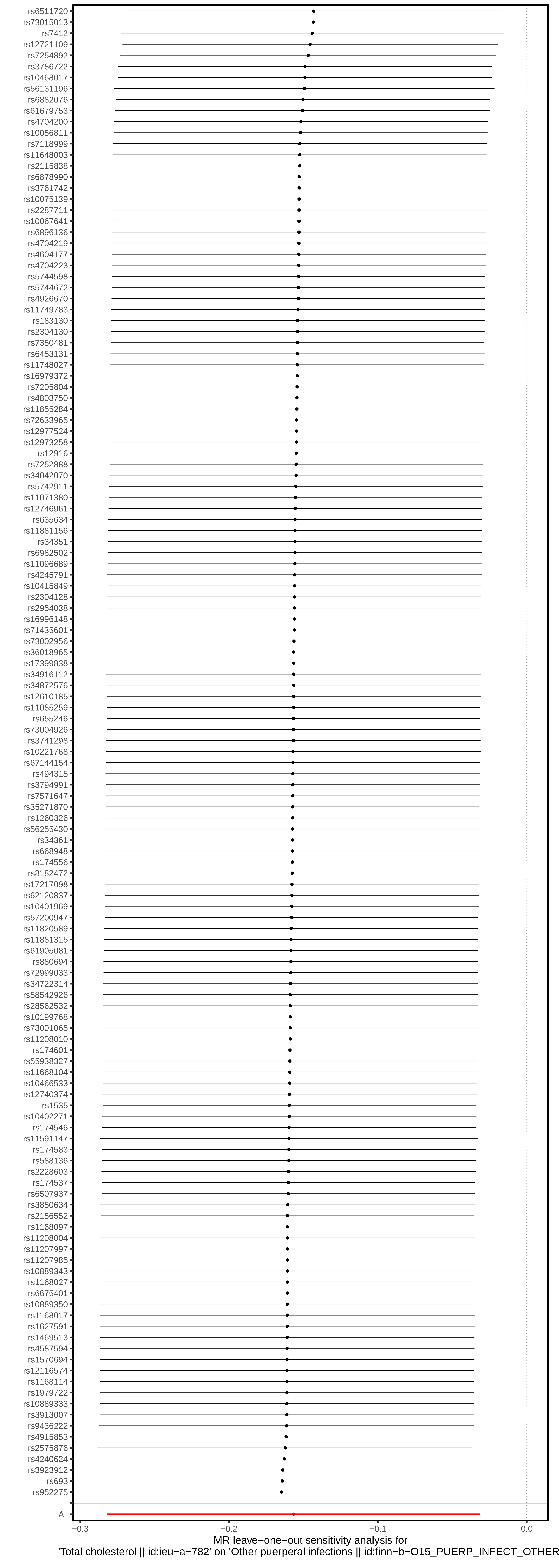
rs6511720
rs73015013
rs7412
rs12721109
rs7254892
rs3786722
rs10468017
rs56131196
rs6882076
rs61679753
rs4704200
rs10056811
rs7118999
rs11648003
rs2115838
rs6878990
rs3761742
rs10075139
rs2287711
rs10067641
rs6896136
rs4704219
rs4604177
rs4704223
rs5744598
rs5744672
rs4926670
rs11749783
rs183130
rs2304130
rs7350481
rs6453131
rs11748027
rs16979372
rs7205804
rs4803750
rs11855284
rs72633965
rs12977524
rs12973258
rs12916
rs7252888
rs34042070
rs5742911
rs11071380
rs12746961
rs635634
rs11881156
rs34351
rs6982502
rs11096689
rs4245791
rs10415849
rs2304128
rs2954038
rs16996148
rs71435601
rs73002956
rs36018965
rs17399838
rs34916112
rs34872576
rs12610185
rs11085259
rs655246
rs73004926
rs3741298
rs10221768
rs67144154
rs494315
rs3794991
rs7571647
rs35271870
rs1260326
rs56255430
rs34361
rs668948
rs174556
rs8182472
rs17217098
rs62120837
rs10401969
rs57200947
rs11820589
rs11881315
rs61905081
rs880694
rs72999033
rs34722314
rs58542926
rs28562532
rs10199768
rs73001065
rs11208010
rs174601
rs55938327
rs11668104
rs10466533
rs12740374
rs1535
rs10402271
rs174546
rs11591147
rs174583
rs588136
rs2228603
rs174537
rs6507937
rs3850634
rs2156552
rs1168097
rs11208004
rs11207997
rs11207985
rs10889343
rs1168027
rs6675401
rs10889350
rs1168017
rs1627591
rs1469513
rs4587594
rs1570694
rs12116574
rs1168114
rs1979722
rs10889333
rs3913007
rs9436222
rs4915853
rs2575876
rs4240624
rs3923912
rs693
rs952275
All
−0.3
−0.2
−0.1
0.0
MR leave−one−out sensitivity analysis for
'Total cholesterol || id:ieu−a−782' on 'Other puerperal infections || id:finn−b−O15_PUERP_INFECT_OTHER

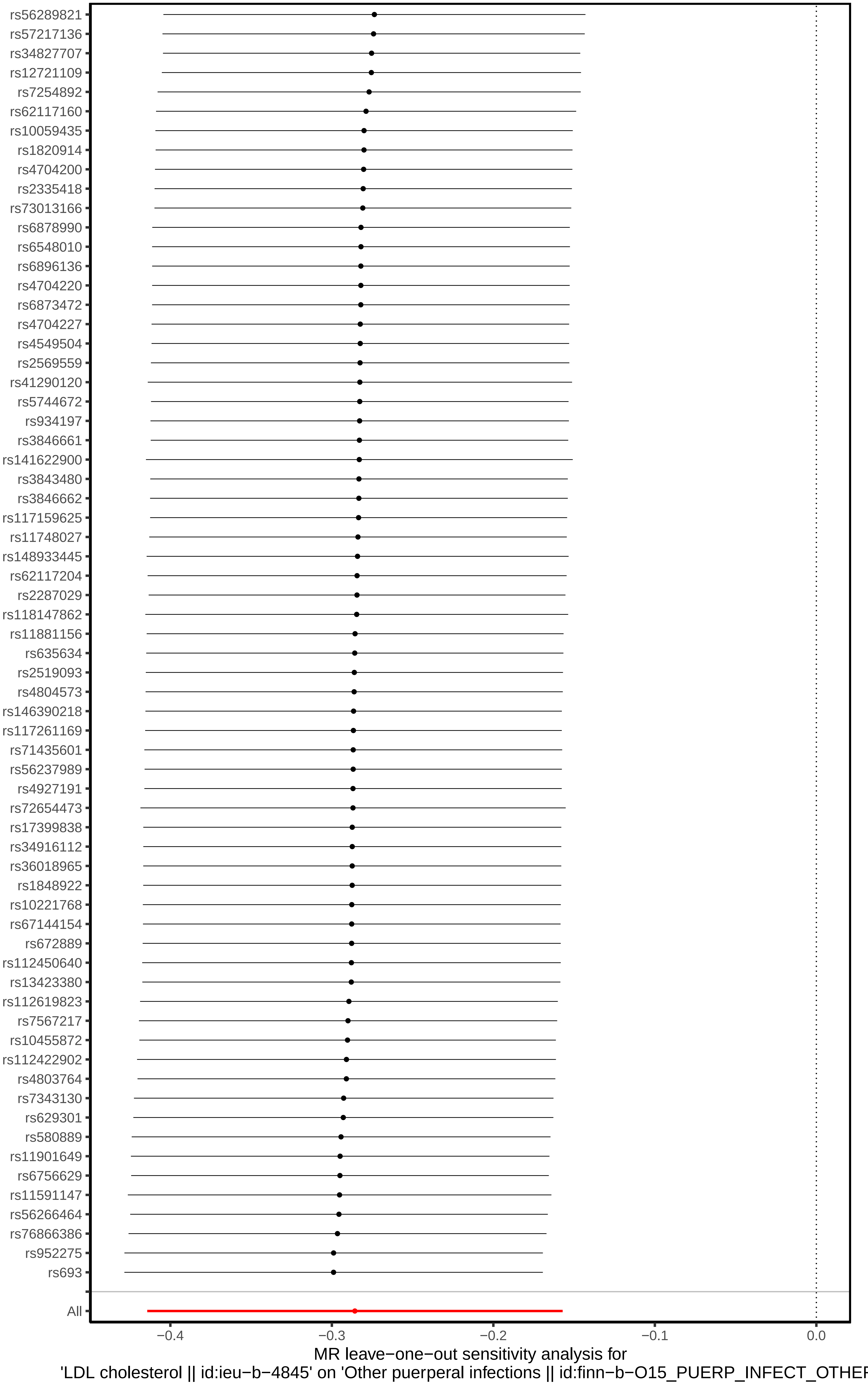

rs56289821
rs57217136
rs34827707
rs12721109
rs7254892
rs62117160
rs10059435
rs1820914
rs4704200
rs2335418
rs73013166
rs6878990
rs6548010
rs6896136
rs4704220
rs6873472
rs4704227
rs4549504
rs2569559
rs41290120
rs5744672
rs934197
rs3846661
rs141622900
rs3843480
rs3846662
rs117159625
rs11748027
rs148933445
rs62117204
rs2287029
rs118147862
rs11881156
rs635634
rs2519093
rs4804573
rs146390218
rs117261169
rs71435601
rs56237989
rs4927191
rs72654473
rs17399838
rs34916112
rs36018965
rs1848922
rs10221768
rs67144154
rs672889
rs112450640
rs13423380
rs112619823
rs7567217
rs10455872
rs112422902
rs4803764
rs7343130
rs629301
rs580889
rs11901649
rs6756629
rs11591147
rs56266464
rs76866386
rs952275
rs693
All
−0.4
−0.3
−0.2
−0.1
0.0
MR leave−one−out sensitivity analysis for
'LDL cholesterol || id:ieu−b−4845' on 'Other puerperal infections || id:finn−b−O15_PUERP_INFECT_OTHER'

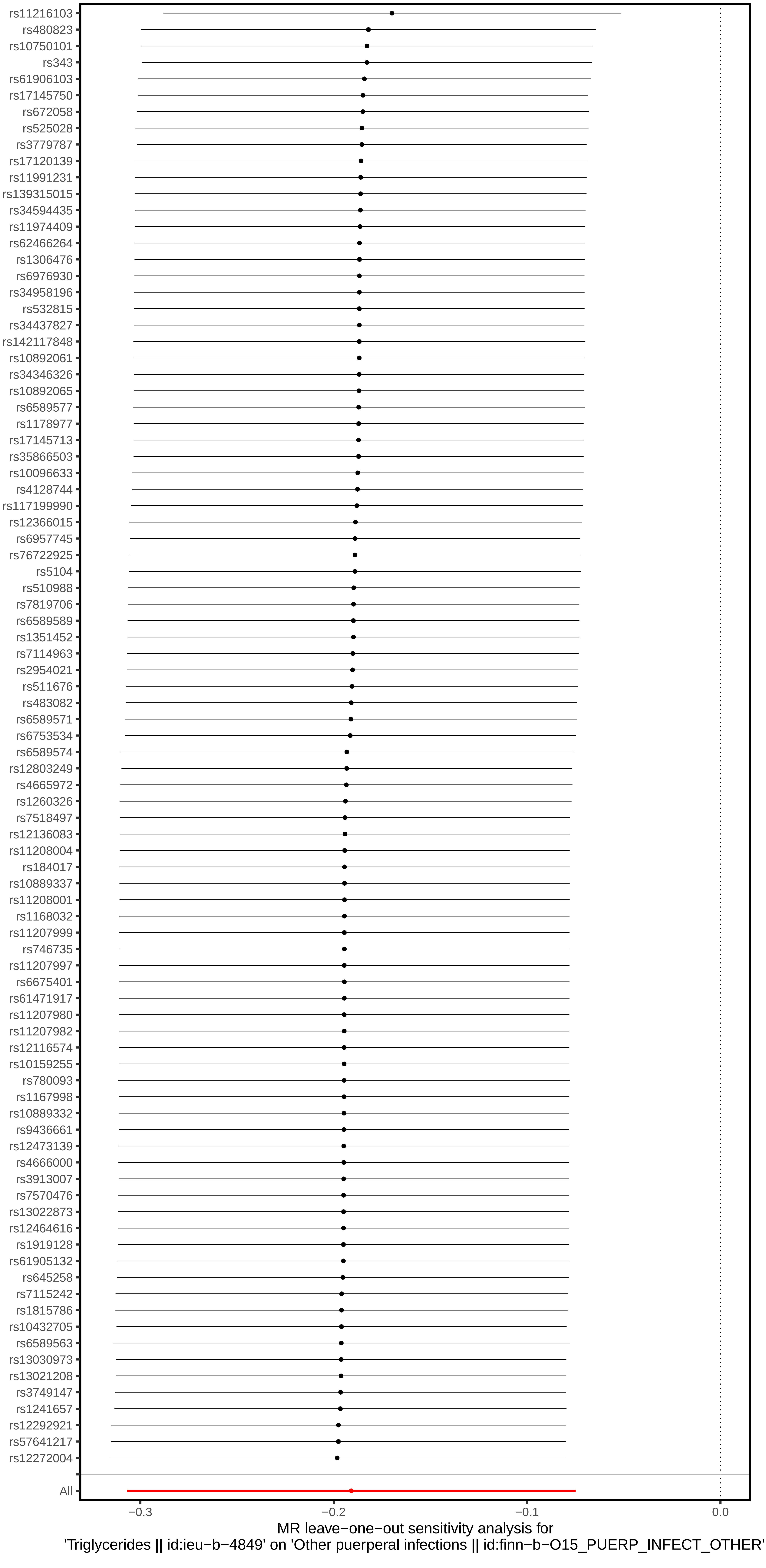

rs11216103
rs480823
rs10750101
rs343
rs61906103
rs17145750
rs672058
rs525028
rs3779787
rs17120139
rs11991231
rs139315015
rs34594435
rs11974409
rs62466264
rs1306476
rs6976930
rs34958196
rs532815
rs34437827
rs142117848
rs10892061
rs34346326
rs10892065
rs6589577
rs1178977
rs17145713
rs35866503
rs10096633
rs4128744
rs117199990
rs12366015
rs6957745
rs76722925
rs5104
rs510988
rs7819706
rs6589589
rs1351452
rs7114963
rs2954021
rs511676
rs483082
rs6589571
rs6753534
rs6589574
rs12803249
rs4665972
rs1260326
rs7518497
rs12136083
rs11208004
rs184017
rs10889337
rs11208001
rs1168032
rs11207999
rs746735
rs11207997
rs6675401
rs61471917
rs11207980
rs11207982
rs12116574
rs10159255
rs780093
rs1167998
rs10889332
rs9436661
rs12473139
rs4666000
rs3913007
rs7570476
rs13022873
rs12464616
rs1919128
rs61905132
rs645258
rs7115242
rs1815786
rs10432705
rs6589563
rs13030973
rs13021208
rs3749147
rs1241657
rs12292921
rs57641217
rs12272004
All
−0.3
−0.2
−0.1
0.0
MR leave−one−out sensitivity analysis for
'Triglycerides || id:ieu−b−4849' on 'Other puerperal infections || id:finn−b−O15_PUERP_INFECT_OTHER'

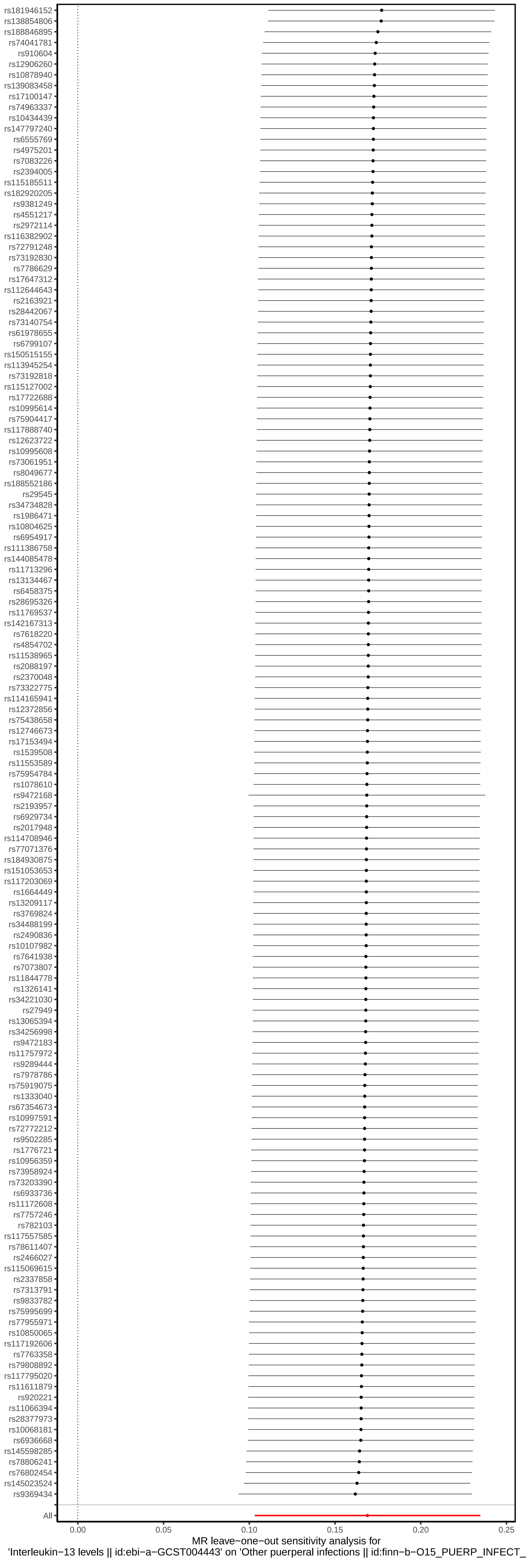
rs181946152
rs138854806
rs188846895
rs74041781
rs910604
rs12906260
rs10878940
rs139083458
rs17100147
rs74963337
rs10434439
rs147797240
rs6555769
rs4975201
rs7083226
rs2394005
rs115185511
rs182920205
rs9381249
rs4551217
rs2972114
rs116382902
rs72791248
rs73192830
rs7786629
rs17647312
rs112644643
rs2163921
rs28442067
rs73140754
rs61978655
rs6799107
rs150515155
rs113945254
rs73192818
rs115127002
rs17722688
rs10995614
rs75904417
rs117888740
rs12623722
rs10995608
rs73061951
rs8049677
rs188552186
rs29545
rs34734828
rs1986471
rs10804625
rs6954917
rs111386758
rs144085478
rs11713296
rs13134467
rs6458375
rs28695326
rs11769537
rs142167313
rs7618220
rs4854702
rs11538965
rs2088197
rs2370048
rs73322775
rs114165941
rs12372856
rs75438658
rs12746673
rs17153494
rs1539508
rs11553589
rs75954784
rs1078610
rs9472168
rs2193957
rs6929734
rs2017948
rs114708946
rs77071376
rs184930875
rs151053653
rs117203069
rs1664449
rs13209117
rs3769824
rs34488199
rs2490836
rs10107982
rs7641938
rs7073807
rs11844778
rs1326141
rs34221030
rs27949
rs13065394
rs34256998
rs9472183
rs11757972
rs9289444
rs7978786
rs75919075
rs1333040
rs67354673
rs10997591
rs72772212
rs9502285
rs1776721
rs10956359
rs73958924
rs73203390
rs6933736
rs11172608
rs7757246
rs782103
rs117557585
rs78611407
rs2466027
rs115069615
rs2337858
rs7313791
rs9833782
rs75995699
rs77955971
rs10850065
rs117192606
rs7763358
rs79808892
rs117795020
rs11611879
rs920221
rs11066394
rs28377973
rs10068181
rs6936668
rs145598285
rs78806241
rs76802454
rs145023524
rs9369434
All
0.00
0.05
0.10
0.15
0.20
0.25
MR leave−one−out sensitivity analysis for
'Interleukin−13 levels || id:ebi−a−GCST004443' on 'Other puerperal infections || id:finn−b−O15_PUERP_INFECT_

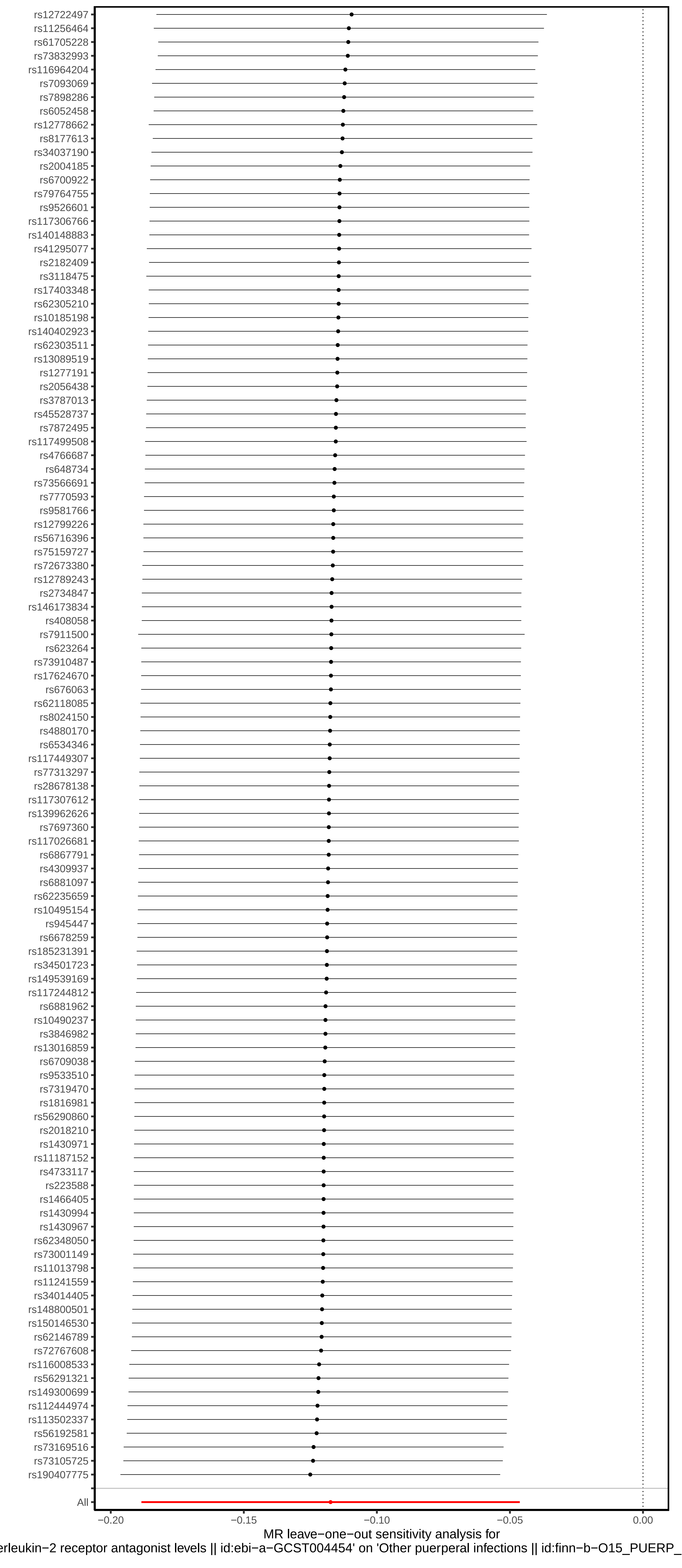
rs12722497
rs11256464
rs61705228
rs73832993
rs116964204
rs7093069
rs7898286
rs6052458
rs12778662
rs8177613
rs34037190
rs2004185
rs6700922
rs79764755
rs9526601
rs117306766
rs140148883
rs41295077
rs2182409
rs3118475
rs17403348
rs62305210
rs10185198
rs140402923
rs62303511
rs13089519
rs1277191
rs2056438
rs3787013
rs45528737
rs7872495
rs117499508
rs4766687
rs648734
rs73566691
rs7770593
rs9581766
rs12799226
rs56716396
rs75159727
rs72673380
rs12789243
rs2734847
rs146173834
rs408058
rs7911500
rs623264
rs73910487
rs17624670
rs676063
rs62118085
rs8024150
rs4880170
rs6534346
rs117449307
rs77313297
rs28678138
rs117307612
rs139962626
rs7697360
rs117026681
rs6867791
rs4309937
rs6881097
rs62235659
rs10495154
rs945447
rs6678259
rs185231391
rs34501723
rs149539169
rs117244812
rs6881962
rs10490237
rs3846982
rs13016859
rs6709038
rs9533510
rs7319470
rs1816981
rs56290860
rs2018210
rs1430971
rs11187152
rs4733117
rs223588
rs1466405
rs1430994
rs1430967
rs62348050
rs73001149
rs11013798
rs11241559
rs34014405
rs148800501
rs150146530
rs62146789
rs72767608
rs116008533
rs56291321
rs149300699
rs112444974
rs113502337
rs56192581
rs73169516
rs73105725
rs190407775
All
−0.20
−0.15
−0.10
−0.05
0.00
MR leave−one−out sensitivity analysis for
erleukin−2 receptor antagonist levels || id:ebi−a−GCST004454' on 'Other puerperal infections || id:finn−b−O15_PUERP_

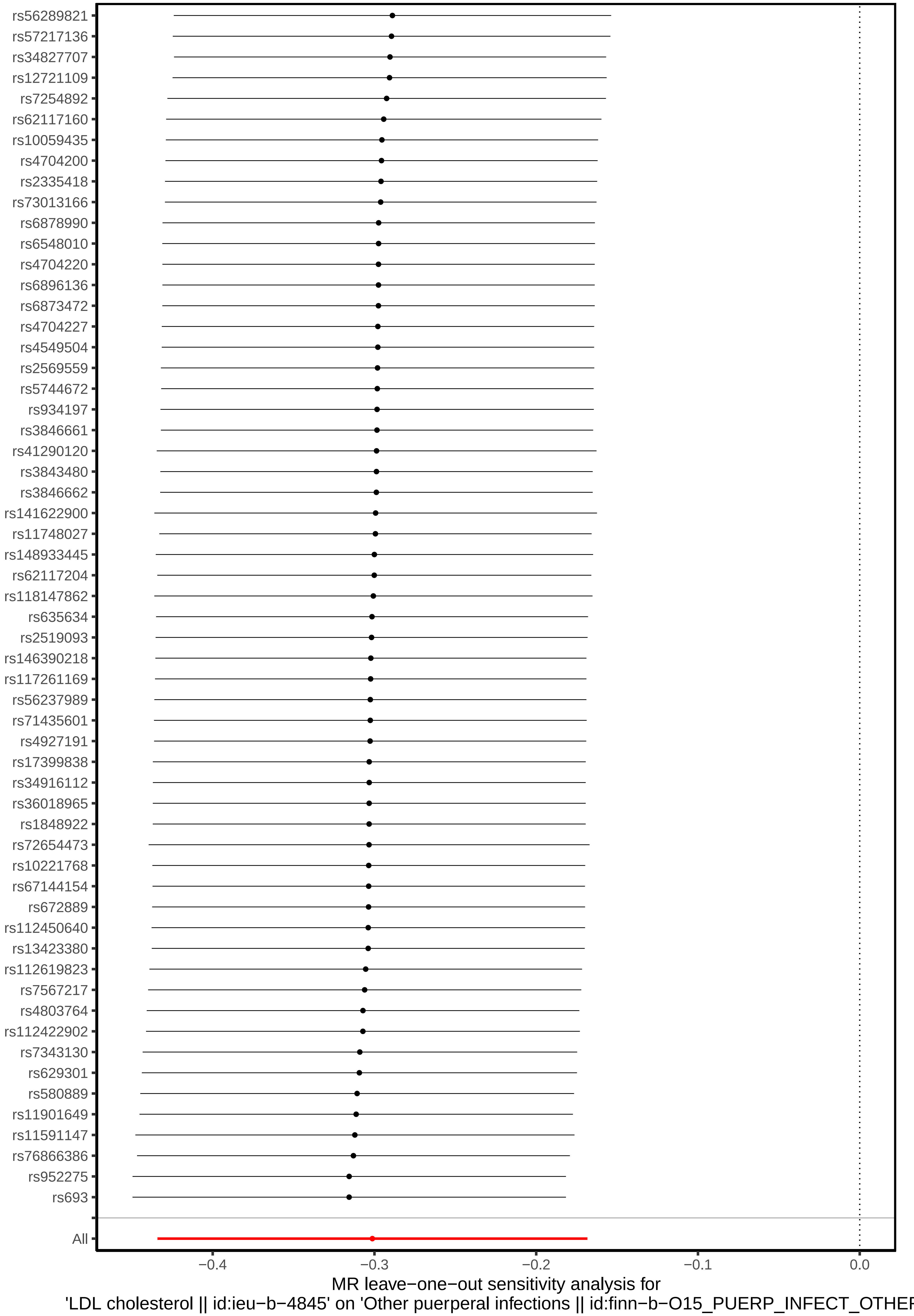
rs56289821
rs57217136
rs34827707
rs12721109
rs7254892
rs62117160
rs10059435
rs4704200
rs2335418
rs73013166
rs6878990
rs6548010
rs4704220
rs6896136
rs6873472
rs4704227
rs4549504
rs2569559
rs5744672
rs934197
rs3846661
rs41290120
rs3843480
rs3846662
rs141622900
rs11748027
rs148933445
rs62117204
rs118147862
rs635634
rs2519093
rs146390218
rs117261169
rs56237989
rs71435601
rs4927191
rs17399838
rs34916112
rs36018965
rs1848922
rs72654473
rs10221768
rs67144154
rs672889
rs112450640
rs13423380
rs112619823
rs7567217
rs4803764
rs112422902
rs7343130
rs629301
rs580889
rs11901649
rs11591147
rs76866386
rs952275
rs693
All
−0.4
−0.3
−0.2
−0.1
0.0
MR leave−one−out sensitivity analysis for
'LDL cholesterol || id:ieu−b−4845' on 'Other puerperal infections || id:finn−b−O15_PUERP_INFECT_OTHEF

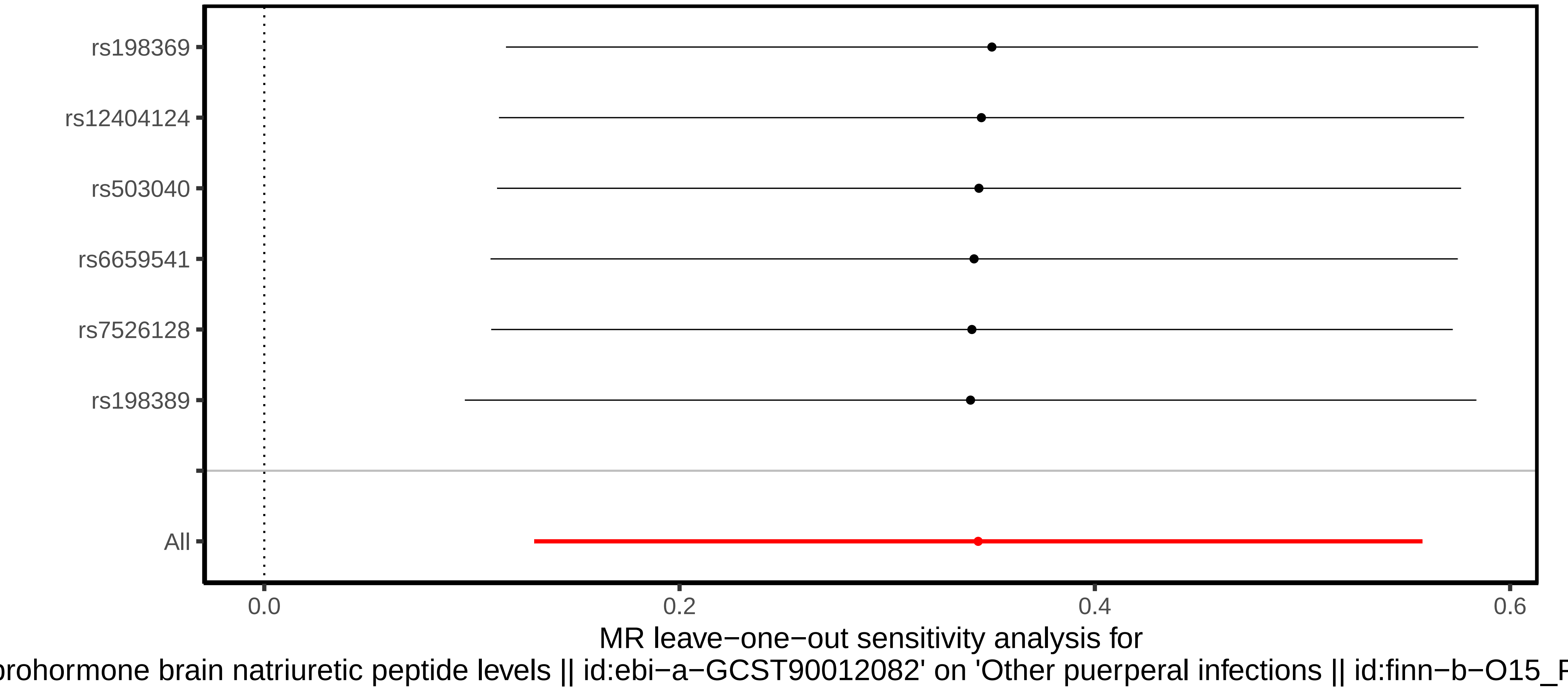
rs198369
rs12404124
rs503040
rs6659541
rs7526128
rs198389
All
0.0
0.2
0.4
0.6
MR leave−one−out sensitivity analysis for
prohormone brain natriuretic peptide levels || id:ebi−a−GCST90012082' on 'Other puerperal infections || id:finn−b−O15_F

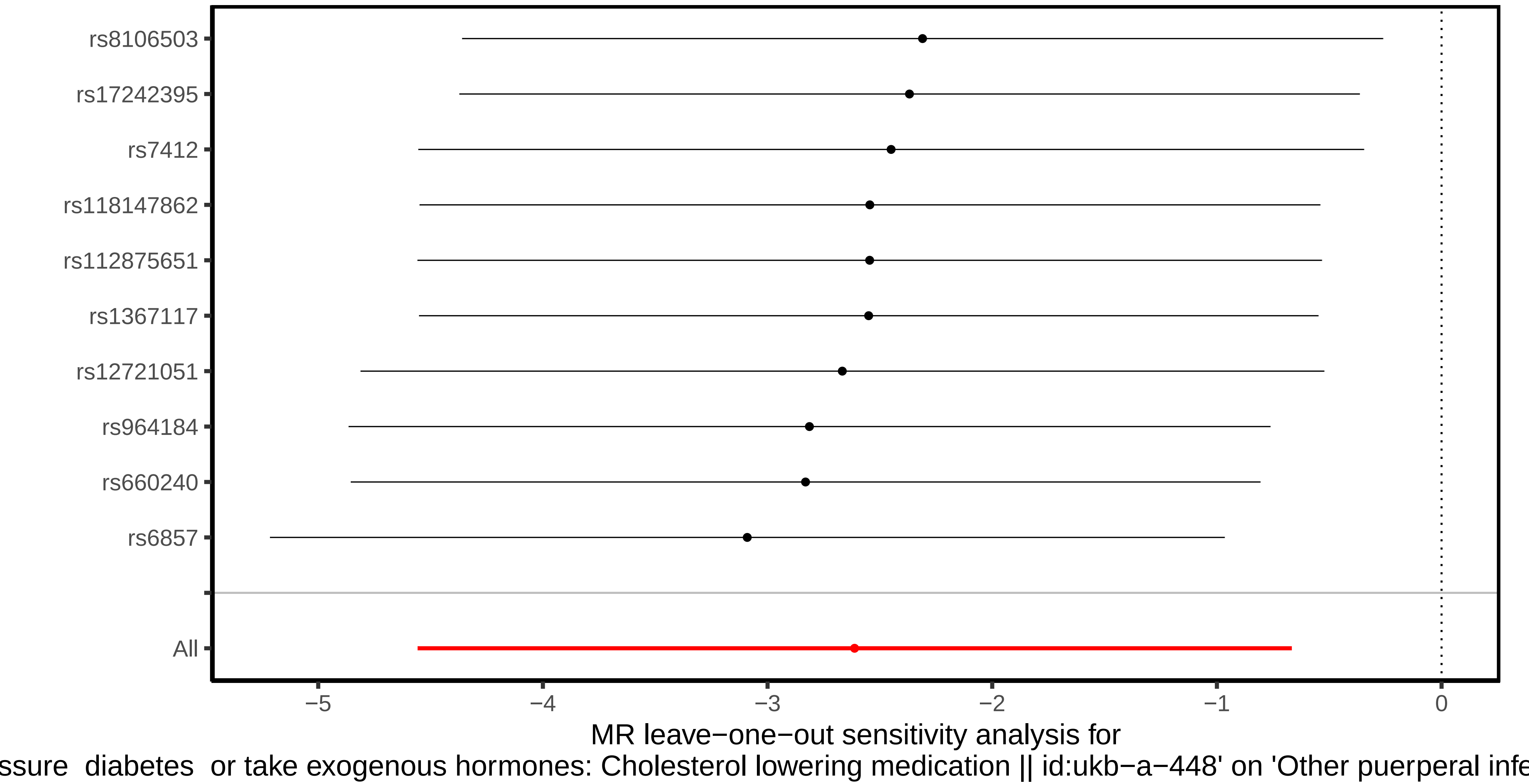

rs8106503
rs17242395
rs7412
rs118147862
rs112875651
rs1367117
rs12721051
rs964184
rs660240
rs6857
All
−5
−4
−3
−2
−1
0
MR leave−one−out sensitivity analysis for
ssure diabetes or take exogenous hormones: Cholesterol lowering medication || id:ukb−a−448' on 'Other puerperal infe

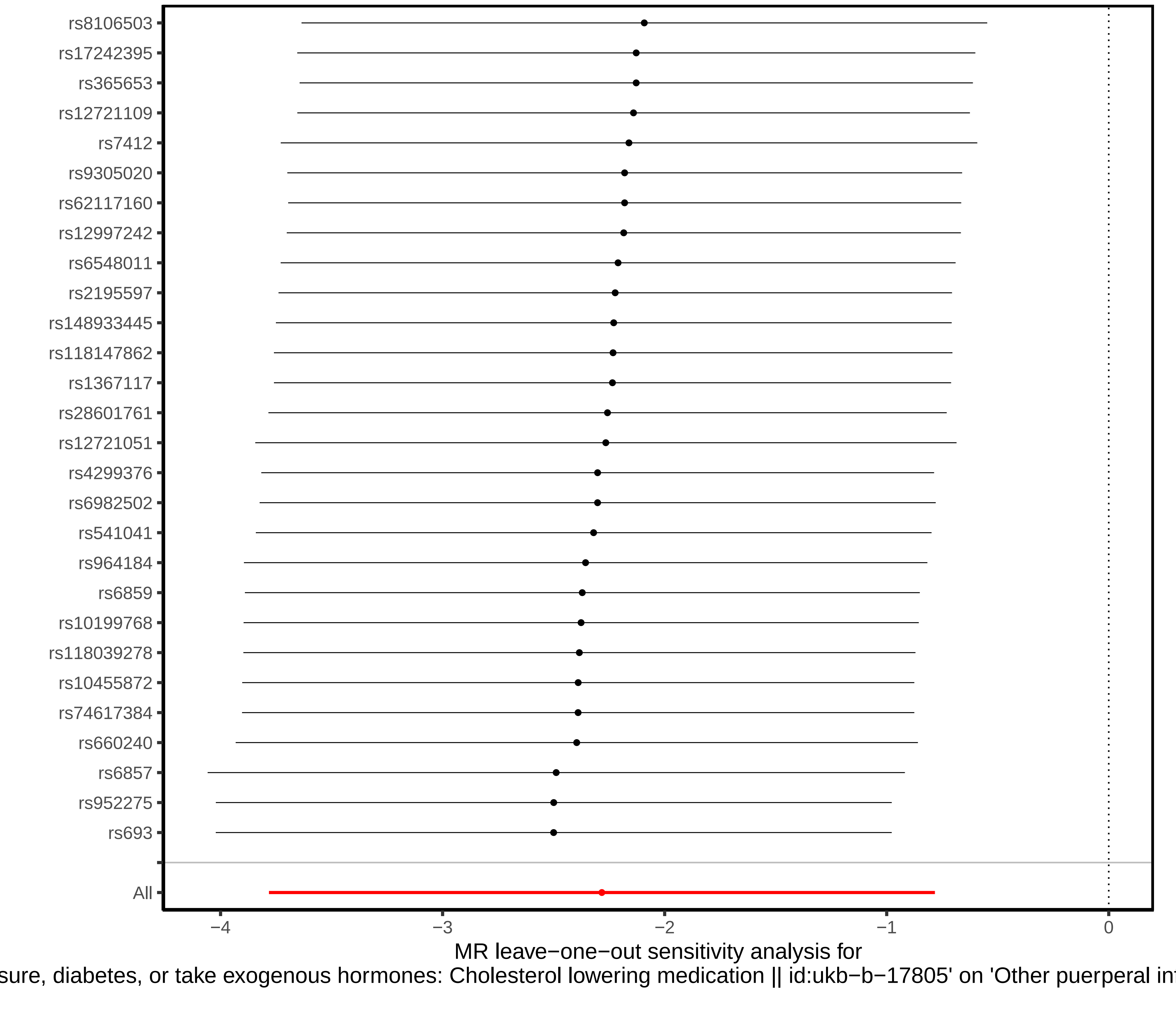
rs8106503
rs17242395
rs365653
rs12721109
rs7412
rs9305020
rs62117160
rs12997242
rs6548011
rs2195597
rs148933445
rs118147862
rs1367117
rs28601761
rs12721051
rs4299376
rs6982502
rs541041
rs964184
rs6859
rs10199768
rs118039278
rs10455872
rs74617384
rs660240
rs6857
rs952275
rs693
All
−4
−3
−2
−1
0
MR leave−one−out sensitivity analysis for
sure, diabetes, or take exogenous hormones: Cholesterol lowering medication || id:ukb−b−17805' on 'Other puerperal inf

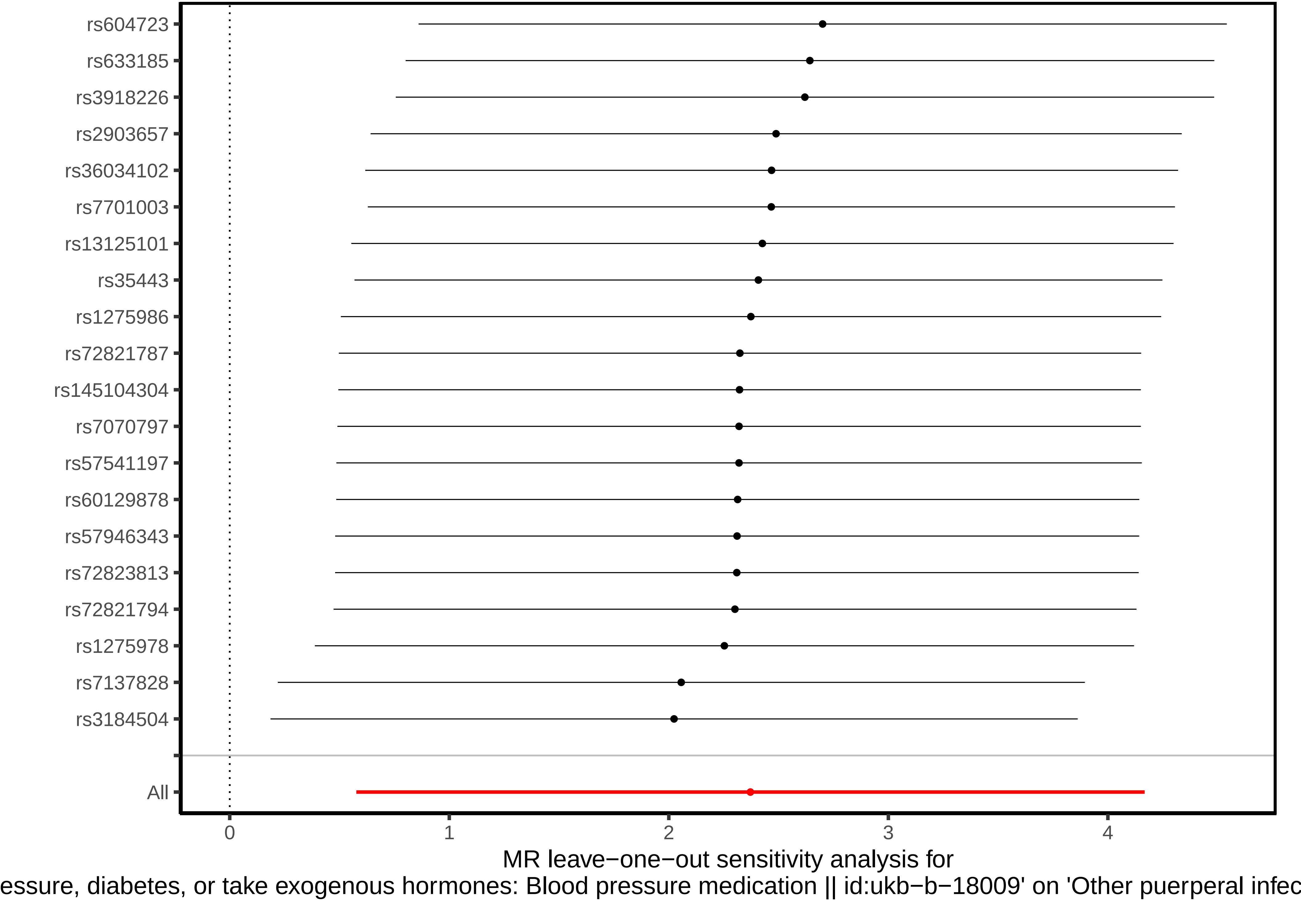
rs604723
rs633185
rs3918226
rs2903657
rs36034102
rs7701003
rs13125101
rs35443
rs1275986
rs72821787
rs145104304
rs7070797
rs57541197
rs60129878
rs57946343
rs72823813
rs72821794
rs1275978
rs7137828
rs3184504
All
0
1
2
3
4
MR leave−one−out sensitivity analysis for
essure, diabetes, or take exogenous hormones: Blood pressure medication || id:ukb−b−18009' on 'Other puerperal infec

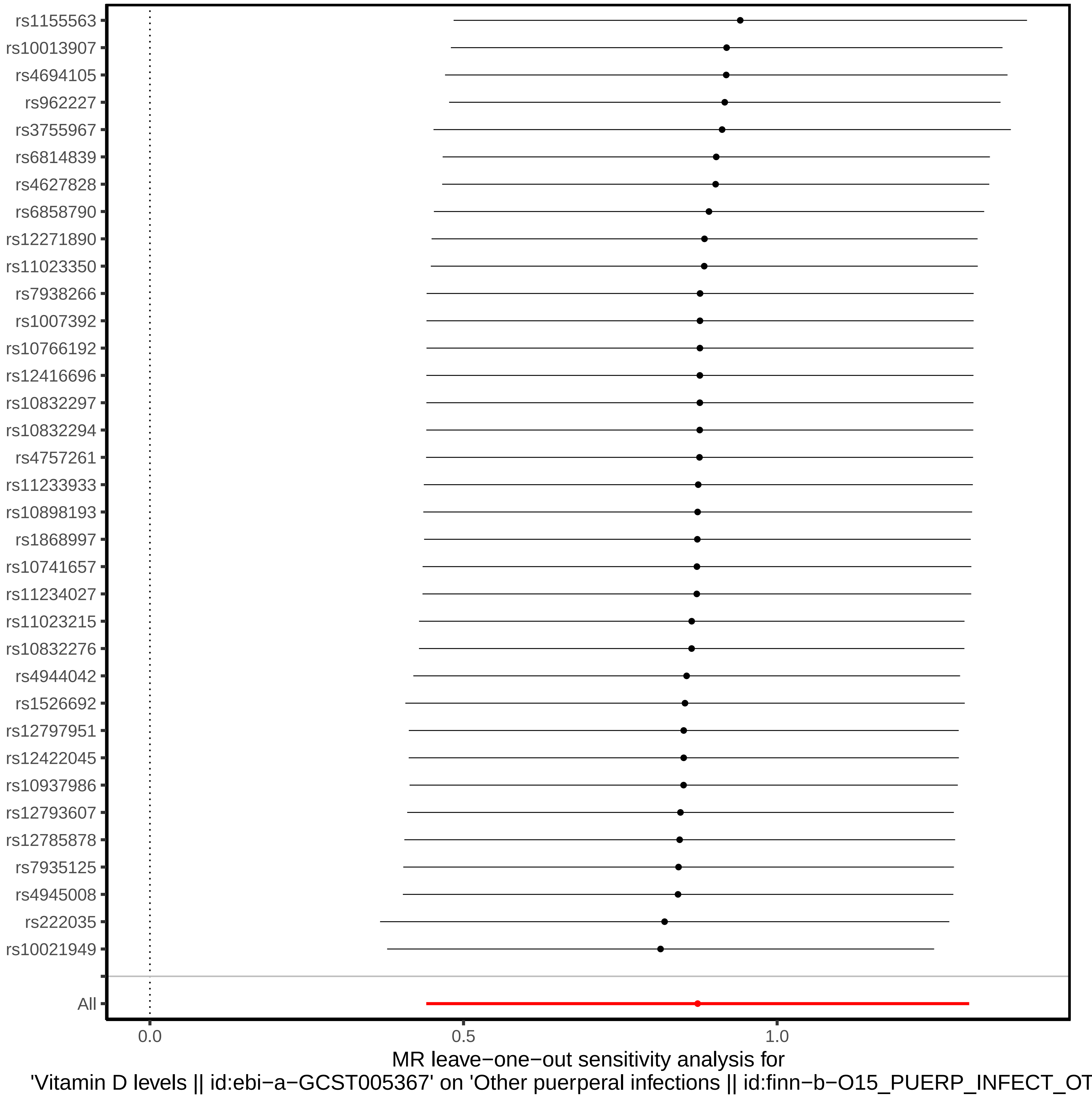

rs1155563
rs10013907
rs4694105
rs962227
rs3755967
rs6814839
rs4627828
rs6858790
rs12271890
rs11023350
rs7938266
rs1007392
rs10766192
rs12416696
rs10832297
rs10832294
rs4757261
rs11233933
rs10898193
rs1868997
rs10741657
rs11234027
rs11023215
rs10832276
rs4944042
rs1526692
rs12797951
rs12422045
rs10937986
rs12793607
rs12785878
rs7935125
rs4945008
rs222035
rs10021949
All
0.0
0.5
1.0
MR leave−one−out sensitivity analysis for
'Vitamin D levels || id:ebi−a−GCST005367' on 'Other puerperal infections || id:finn−b−O15_PUERP_INFECT_OT
