# Supplementary material for: Global burden, projections, and causal factors of maternal sepsis and other maternal infections: A comprehensive epidemiological and mendelian randomization study
Source: PLoS Negl Trop Dis. 2026 May 27;20(5):e0014374. doi: 10.1371/journal.pntd.0014374 (PMC13229374; doi:10.1371/journal.pntd.0014374)
Supplement: S1 Checklist — Skrivankova VW, Richmond RC, Woolf BAR, et al. Strengthening the Reporting of Observational Studies in Epidemiology Using Mendelian Randomization: The STROBE-MR Statement. JAMA. 2021;326(16):1614–1621. doi:10.1001/jama.2021.18236. (DOCX) [file pntd.0014374.s020.docx]

**STROBE-MR checklist of recommended items to address in reports of Mendelian randomization studies**^1^ ^2^

| **Item No.** | **Section** | **Checklist item** | **Page No.** | **Relevant text from manuscript** |
| --- | --- | --- | --- | --- |
| 1 | **TITLE and ABSTRACT** | Indicate Mendelian randomization (MR) as the study’s design in the title and/or the abstract if that is a main purpose of the study | 1-2 | Title: Global Burden, Projections, and Causal factors of Maternal Sepsis and Other Maternal Infections: A Comprehensive Epidemiological and Mendelian Randomization Study  Abstract：Background: Maternal sepsis and other maternal infections (MSMI) remain major contributors to global maternal morbidity and mortality. However, the integration of epidemiological trends with causal inference evidence remains limited.  Methods: Using data from the Global Burden of Disease (GBD) 2021 study, we assessed temporal trends in MSMI burden from 1990 to 2021 and projected future patterns using ARIMA and Bayesian age–period–cohort (BAPC) models. In parallel, we conducted a two-sample multivariable Mendelian randomization (MVMR) analysis to evaluate the causal effects of inflammatory biomarkers and related factors on MSMI risk.  Results: Although age-standardized rates declined globally, absolute case numbers increased in low-SDI regions, largely driven by population growth. Forecasting results differed between ARIMA and BAPC models, reflecting distinct underlying assumptions regarding temporal dynamics. MVMR analysis identified inflammatory biomarkers, including CRP, IL-13, IL-10, RANTES, and NT-proBNP, as key causal factors associated with MSMI.  Conclusions: This study provides the first integrated framework combining global disease burden analysis with multivariable MR. By linking population-level trends with causal inference, our findings offer dual evidence to support targeted prevention strategies and advance precision public health interventions for MSMI. |
|  | **INTRODUCTION** |  |  |  |
| 2 | **Background** | Explain the scientific background and rationale for the reported study. What is the exposure? Is a potential causal relationship between exposure and outcome plausible? Justify why MR is a helpful method to address the study question | 4 | Disparities in disease burden provide policymakers with an evidence-based foundation for implementing targeted interventions and preventive measures in specific populations. Nevertheless, patients affected by MSMI, along with their families, often seek to understand the underlying causes of these conditions to adopt effective prevention strategies. Therefore, it is essential to thoroughly investigate the risk factors contributing to MSMI for developing effective interventions aimed at reducing associated risks. Unlike traditional observational studies, which are susceptible to residual confounding and various biases, Mendelian randomization (MR) is an epidemiological approach that uses genetic variants-randomly allocated at conception-as instrumental variables to infer causal relationships between exposures and outcomes [11]. This method substantially reduces reverse causation and confounding biases often introduced by sociodemographic or behavioral factors in conventional observational studies. |
| 3 | **Objectives** | State specific objectives clearly, including pre-specified causal hypotheses (if any). State that MR is a method that, under specific assumptions, intends to estimate causal effects | 4 | In this study, we applied a two-sample MR design to examine bidirectional causal relationships between various potential risk factors and MSMI. Furthermore, multivariable MR was employed to assess the independent effects of multiple significantly associated exposures on MSMI outcomes. Our findings reveal causal links between several modifiable factors and MSMI, which may inform future preventive strategies and clinical guidance. |
|  | **METHODS** |  |  |  |
| 4 | **Study design and data sources** | Present key elements of the study design early in the article. Consider including a table listing sources of data for all phases of the study. For each data source contributing to the analysis, describe the following: | 34-36 | Table 1 |
|  | a) | Setting: Describe the study design and the underlying population, if possible. Describe the setting, locations, and relevant dates, including periods of recruitment, exposure, follow-up, and data collection, when available. | 5-6 | A two-sample MR framework was applied to investigate the causal relationships between variable factors and MSMI. Given that genetic variations may influence multiple correlated exposures, multivariable Mendelian randomization (MVMR) was performed to delineate the direct causal effects of individual variable factors on MSMI. Detailed data source descriptions are provided in Table 1 |
|  | b) | Participants: Give the eligibility criteria, and the sources and methods of selection of participants. Report the sample size, and whether any power or sample size calculations were carried out prior to the main analysis | 6-7 | Instrumental variables for these factors were sourced from multiple large-scale genetic consortia and databases, including the UK Biobank (UKB) [15], the Early Growth Genetics (EGG) Consortium [16], the Genetic Investigation of Anthropometric Traits (GIANT) Consortium [17], the Global Lipids Genetics Consortium (GLGC) [18], the NHGRI-EBI Genome-Wide Association Studies Catalog (GWAS Catalog) [19], the Systematic and Combined Analysis of Olink Proteins (SCALLOP) [20], and the Within Family GWAS Consortium (WFGWC) [21]. Detailed data source descriptions are provided in Table 1. The FinnGen study protocol was approved by Coordinating Ethics Committee of the Hospital District of Helsinki and Uusimaa (number HUS/990/2017), with all participants providing informed consent [14]. |
|  | c) | Describe measurement, quality control and selection of genetic variants | 6-7 | The analysis was grounded in three core MR assumptions: (i) the genetic instruments exhibit strong associations with the exposures of interest; (ii) these instruments are independent of potential confounders; and (iii) they influence the risk of MSMI only via the exposure pathways (Fig.1). As all genetic and outcome data were derived from previously published studies with ethical approvals [13], no additional informed consent was required for the present study.  Based on GWAS summary statistics, single-nucleotide polymorphisms (SNPs) associated with each exposure of interest were selected as instrumental variables. Independent SNPs were identified through a clumping procedure under strict linkage disequilibrium (LD) criteria (P < 5 × 10⁻⁸, r² < 0.001, clumping window = 10,000 kb). For unavailable SNPs in the outcome dataset, proxy variants were identified from the 1000 Genomes European reference panel using a high LD threshold (r² > 0.8), excluding SNPs without suitable proxies. Additionally, palindromic SNPs with intermediate allele frequencies were removed to prevent strand ambiguity. To enhance analytical robustness, SNPs with a minor allele frequency (MAF) < 0.01 were further excluded, thereby minimizing potential bias arising from low-frequency variants that often yield unreliable estimates in GWAS. The proportion of variance explained (R2) for each risk factor by individual SNPs was quantified[13], and the strength of instrumental variables was evaluated using the F-statistic [22]. The F-statistic was calculated as F = R² × (N − 2) / (1 − R²), where R² represents the proportion of variance explained by the genetic instruments and N is the sample size. |
|  | d) | For each exposure, outcome, and other relevant variables, describe methods of assessment and diagnostic criteria for diseases | 4-7 | Genome-wide association study (GWAS) summary statistics for MSMI were obtained from the FinnGen Consortium [14]. The analyzed variable factors encompassed five major categories:   - **Basic characteristics**: birth weight, body mass index (BMI); - **Medical history**: previous smoking status, history of stillbirth, spontaneous miscarriage or termination, number of pregnancy terminations, medication use for cholesterol, blood pressure, diabetes, or exogenous hormones (e.g., cholesterol-lowering medication); - **Routine blood and serum metabolic biomarkers**: high-density lipoprotein (HDL) cholesterol, low-density lipoprotein (LDL) cholesterol, triglycerides (TG), total cholesterol levels or total cholesterol (TC), C-reactive protein levels or C-reactive protein (CRP), vitamin D levels (VitD), N-terminal prohormone of brain natriuretic peptide levels (NT-proBNP); - **Serum inflammatory cytokines**: RANTES levels (RANTES), interleukin-13 levels (IL-13), interleukin-10 levels (IL-10), interleukin-1 receptor antagonist levels (IL-1Ra), interleukin-2 receptor antagonist levels (IL-2Ra), interferon gamma levels (IFN-γ); - **Serum-specific biomarkers**: Pregnancy-specific beta-1-glycoprotein 11 (PSG11), Pregnancy-specific beta-1-glycoprotein 9 (PSG9).   Instrumental variables for these factors were sourced from multiple large-scale genetic consortia and databases, including the UK Biobank (UKB) [15], the Early Growth Genetics (EGG) Consortium [16], the Genetic Investigation of Anthropometric Traits (GIANT) Consortium [17], the Global Lipids Genetics Consortium (GLGC) [18], the NHGRI-EBI Genome-Wide Association Studies Catalog (GWAS Catalog) [19], the Systematic and Combined Analysis of Olink Proteins (SCALLOP) [20], and the Within Family GWAS Consortium (WFGWC) [21]. Detailed data source descriptions are provided in Table 1. The FinnGen study protocol was approved by Coordinating Ethics Committee of the Hospital District of Helsinki and Uusimaa (number HUS/990/2017), with all participants providing informed consent [14].  As previously defined [12], MSMI comprises two distinct entities [12]. Maternal sepsis is characterized by abnormal core body temperature (＜36°C or >38°C) along with signs of shock, such as systolic hypotension (<90 mmHg) and tachycardia (>120 beats per minute). Other maternal infections refer to any non-AIDS-defining and non-sexually transmitted infectious conditions not considered epidemiologically linked to pregnancy. The latter category encompasses disorders such as gestational urinary tract infections, mastitis, candidiasis, and bacterial vaginosis. |
|  | e) | Provide details of ethics committee approval and participant informed consent, if relevant | 7 | The FinnGen study protocol was approved by Coordinating Ethics Committee of the Hospital District of Helsinki and Uusimaa (number HUS/990/2017), with all participants providing informed consent [14]. |
| 5 | **Assumptions** | Explicitly state the three core IV assumptions for the main analysis (relevance, independence and exclusion restriction) as well assumptions for any additional or sensitivity analysis | 6 | The analysis was grounded in three core MR assumptions: (i) the genetic instruments exhibit strong associations with the exposures of interest; (ii) these instruments are independent of potential confounders; and (iii) they influence the risk of MSMI only via the exposure pathways (Fig.1). |
| 6 | **Statistical methods: main analysis** | Describe statistical methods and statistics used | 7-11 |  |
|  | a) | Describe how quantitative variables were handled in the analyses (i.e., scale, units, model) | 9 | A two-sample MR framework was applied to investigate the causal relationships between variable factors and MSMI. After harmonizing effect alleles and directions using the “harmonise_data” function from the TwoSampleMR package in R software (version 4.1.0), MR analyses were conducted through the “mr” function, incorporating five complementary methods: MR-Egger regression (MR-Egger) [25], weighted median (WM) [25], inverse-variance weighted (IVW) [26], simple mode, and weighted mode [26]. MR analyses were performed separately for each exposure factor, with the IVW method serving as the primary analytical approach for causal inference. |
|  | b) | Describe how genetic variants were handled in the analyses and, if applicable, how their weights were selected | 9-10 | MR analyses were performed separately for each exposure factor, with the IVW method serving as the primary analytical approach for causal inference.  Heterogeneity among instrumental variables was assessed using Cochran’s Q test via the "mr_heterogeneity" function. P-values > 0.05 indicated absence of significant heterogeneity, supporting the use of fixed-effect inverse-variance weighted (IVW) estimates. In this study, all Q test results were non-significant (P > 0.05), confirming homogeneity across variables [27]. Horizontal pleiotropy was evaluated using MR-Egger regression and MR-PRESSO through the "mr_pleiotropy_test" function. P > 0.05 in both tests indicated no evidence of directional pleiotropy, thereby ensuring causal estimate reliability [25,28]. Finally, causal directionality was verified using MR-Steiger analysis, which confirmed the presumed direction (exposure → outcome) by demonstrating greater variance explained in exposure than outcome. Sensitivity analysis was conducted using the “mr_leaveoneout” function, which removes each SNP one by one and evaluates its impact on the overall results. Notably, if the results change significantly after removing a particular SNP, this indicates that the SNP is sensitive. In this study, no SNPs with significant deviation were found, indicating that the analysis results are stable and reliable. |
|  | c) | Describe the MR estimator (e.g. two-stage least squares, Wald ratio) and related statistics. Detail the included covariates and, in case of two-sample MR, whether the same covariate set was used for adjustment in the two samples | 10 | Given that genetic variations may influence multiple correlated exposures, multivariable Mendelian randomization (MVMR) was performed to delineate the direct causal effects of individual variable factors on MSMI. This approach isolates the independent contribution of each exposure to disease risk [29]. To mitigate potential collinearity among exposures, the least absolute shrinkage and selection operator (LASSO) was applied for variable selection and dimensionality reduction. This method effectively identified a subset of influential exposures by retaining factors significantly associated with MSMI while excluding those with negligible effects [30]. The optimal tuning parameter (λ) was determined using 10-fold cross-validation, selecting the value that minimized the mean cross-validated error. Variables with non-zero coefficients were retained for subsequent multivariable MR analysis. |
|  | d) | Explain how missing data were addressed | 9 | This study utilized publicly available aggregated data from large-scale GWAS consortia (such as UK Biobank, FinnGen, GIANT, etc.). The original GWASs had undergone strict quality control at the individual level, including the exclusion of individuals with genotype detection rates below 95%, missing phenotype data, or abnormal values. Therefore, the aggregated data used in this MR analysis itself does not contain issues related to missing data at the individual level. |
|  | e) | If applicable, indicate how multiple testing was addressed |  | not available |
| 7 | **Assessment of assumptions** | Describe any methods or prior knowledge used to assess the assumptions or justify their validity | 9-10 | Heterogeneity among instrumental variables was assessed using Cochran’s Q test via the "mr_heterogeneity" function. P-values > 0.05 indicated absence of significant heterogeneity, supporting the use of fixed-effect inverse-variance weighted (IVW) estimates. In this study, all Q test results were non-significant (P > 0.05), confirming homogeneity across variables [27]. Horizontal pleiotropy was evaluated using MR-Egger regression and MR-PRESSO through the "mr_pleiotropy_test" function. P > 0.05 in both tests indicated no evidence of directional pleiotropy, thereby ensuring causal estimate reliability [25,28]. Finally, causal directionality was verified using MR-Steiger analysis, which confirmed the presumed direction (exposure → outcome) by demonstrating greater variance explained in exposure than outcome. Sensitivity analysis was conducted using the “mr_leaveoneout” function, which removes each SNP one by one and evaluates its impact on the overall results. Notably, if the results change significantly after removing a particular SNP, this indicates that the SNP is sensitive. In this study, no SNPs with significant deviation were found, indicating that the analysis results are stable and reliable. |
| 8 | **Sensitivity analyses and additional analyses** | Describe any sensitivity analyses or additional analyses performed (e.g. comparison of effect estimates from different approaches, independent replication, bias analytic techniques, validation of instruments, simulations) | 7-10 | Spearman’s rank correlation analysis was employed to investigate the association between the Socio-demographic Index (SDI) and the burden of MSMI. The SDI is a composite indicator of a region’s development level, ranging from 0 (lowest) to 1 (highest), based on lag-distributed income per capita, average educational attainment, and total fertility rate. Given the non-normal distribution of burden indicators across countries, Spearman’s rank correlation was selected as it is a non-parametric method that does not assume linearity or normality, and is robust to outliers. All correlation analyses were performed at the country level, including all 204 countries and territories in the GBD study for the year 1990 and 2021. For each pair of variables (SDI vs. each burden indicator), Spearman’s correlation coefficient (r) and the corresponding two-tailed p-value were calculated. A p-value < 0.05 was considered statistically significant. All statistical analyses were conducted using R software (version 4.5.3) with the “cor.test” function [12]. Decomposition analysis was performed to quantify the contributions of demographic factors and epidemiological changes to the disparities in the burden of MSMI across different SDI quintiles in 2021. Using the Das Gupta decomposition method, the difference in outcome measure between each SDI quintile (low, low-middle, middle, high-middle, and high SDI) and the global average was partitioned into three components: (1) population structure (aging) , reflecting differences in the age composition of populations; (2) population size (growth) , reflecting differences in total population across SDI quintiles; and (3) epidemiological changes, reflecting differences in age-specific rates of the outcome measure. Absolute contributions (the number of cases attributable to each factor) and relative contributions (the percentage of the total disparity explained by each factor) were calculated for each SDI quintile. All decomposition analyses were conducted using R software (version 4.5.3) with the “Dasgupta” function from the “decompose” package [12].  Sensitivity analysis was conducted using the “mr_leaveoneout” function, which removes each SNP one by one and evaluates its impact on the overall results. Notably, if the results change significantly after removing a particular SNP, this indicates that the SNP is sensitive. In this study, no SNPs with significant deviation were found, indicating that the analysis results are stable and reliable. |
| 9 | **Software and pre-registration** |  |  |  |
|  | a) | Name statistical software and package(s), including version and settings used | 9 | A two-sample MR framework was applied to investigate the causal relationships between variable factors and MSMI. After harmonizing effect alleles and directions using the “harmonise_data” function from the TwoSampleMR package in R software (version 4.1.0), MR analyses were conducted through the “mr” function, incorporating five complementary methods: MR-Egger regression (MR-Egger) [25], weighted median (WM) [25], inverse-variance weighted (IVW) [26], simple mode, and weighted mode [26]. MR analyses were performed separately for each exposure factor, with the IVW method serving as the primary analytical approach for causal inference. |
|  | b) | State whether the study protocol and details were pre-registered (as well as when and where) | 10 | The study protocol and details do not require pre-registered. |
|  | **RESULTS** |  |  |  |
| 10 | **Descriptive data** |  |  |  |
|  | a) | Report the numbers of individuals at each stage of included studies and reasons for exclusion. Consider use of a flow diagram |  | not available |
|  | b) | Report summary statistics for phenotypic exposure(s), outcome(s), and other relevant variables (e.g. means, SDs, proportions) |  | not available |
|  | c) | If the data sources include meta-analyses of previous studies, provide the assessments of heterogeneity across these studies |  | not available |
|  | d) | For two-sample MR:  i.  Provide justification of the similarity of the genetic variant-exposure associations between the exposure and outcome samples  ii.  Provide information on the number of individuals who overlap between the exposure and outcome studies |  | not available |
| 11 | **Main results** |  |  |  |
|  | a) | Report the associations between genetic variant and exposure, and between genetic variant and outcome, preferably on an interpretable scale | 14-16 | To investigate potential causal links, a range of variable factors were systematically evaluated in relation to MSMI. For maternal sepsis, multiple factors showed statistically significant associations (P < 0.05), as detailed in Table 2. These included lipid profiles, inflammatory markers, medication use, and pregnancy history variables. Using the inverse variance weighted (IVW) method, we identified several factors with odds ratios (OR) > 1, such as LDL cholesterol, previous smoking, RANTES levels, IL-13 levels, IL-10 levels, birth weight, cholesterol-lowering medications, blood pressure medication, and PSG11, indicating their potential roles as risk factors for the development of maternal sepsis. Conversely, those factor with OR < 1, including HDL cholesterol, TG, TC, IL-1Ra levels, IFN-γ levels, PSG9, and number of pregnancy terminations, emerged as potential protective influences against the development of maternal sepsis.  Analysis of other maternal infections revealed distinct causal profiles, as detailed in Table 3. Statistically significant causal relationships (P < 0.05) were identified between multiple factors and other maternal infections, including spontaneous miscarriage or termination, BMI, CRP, LDL cholesterol, TC, TG, IL-13 levels, IL-2Ra levels, NT-proBNP, cholesterol-lowering medications, blood pressure medication, and vitamin D levels. These findings indicated their potential causal roles in the development of other maternal infections. Using the IVW method, factors with OR > 1 were classified as potential risk factors of other maternal infections, such as spontaneous miscarriage or termination, CRP, IL-13 levels, NT-proBNP, blood pressure medication, and vitamin D levels. Conversely, the IVW method found that BMI, LDL cholesterol, total cholesterol, triglycerides, IL-2Ra levels, and cholesterol-lowering medication (all with OR < 1) emerged as potential protective factors, suggesting a negative association with infection risk.  All instrumental variables showed F-statistics greater than 10 (S7 Table), indicating a low risk of weak instrument bias. The results of harmonization analyses in the Mendelian randomization analysis were available in S8 Table. The results of causal effects using five Mendelian randomization methods were summarized in S9 Table. The validity of MR estimates was systematically evaluated through pleiotropy (S10 Table), heterogeneity (S11 Table), and directionality tests (S12 Table). As summarized in Table 4, all Cochran’s Q statistics returned P-values greater than 0.05, indicating negligible heterogeneity among instrumental variables and supporting the use of fixed-effects IVW models. Similarly, pleiotropy assessment via MR-Egger and MR-PRESSO showed no evidence of horizontal pleiotropy (all P > 0.05, Table 5), confirming the robustness of causal estimates. The detailed results of MR-PRESSO global test were available in S13 Table. Finally, directionality testing verified the assumed causal direction, with outcome variance consistently lower than exposure variance across all analyses (Table 6) .The results of the sensitivity analysis showed that no significantly deviating SNPs were found in this study (S5 Fig for maternal sepsis and S6 Fig for other maternal infections), indicating that the analysis results are stable and reliable.  LASSO regression retained key inflammatory biomarkers, including RANTES, IL-10, CRP, IL-13, and NT-proBNP, for subsequent multivariable MR analysis (MVMR). MVMR analysis was employed to evaluate the independent causal roles of specific inflammatory biomarkers in MSMI. For maternal sepsis, genetically predicted elevations in both RANTES and IL-10 levels demonstrated significant positive associations with disease risk (Table 7). Relatively, in the analysis of other maternal infections, higher levels of CRP, IL-13, and NT-proBNP were causally linked to increased infection risk (Table 7). These results highlight distinct inflammatory pathways contributing to different forms of maternal infection and underscore the potential value of these biomarkers in risk stratification. |
|  | b) | Report MR estimates of the relationship between exposure and outcome, and the measures of uncertainty from the MR analysis, on an interpretable scale, such as odds ratio or relative risk per SD difference | 36-44 | Table 2.  Table 3. |
|  | c) | If relevant, consider translating estimates of relative risk into absolute risk for a meaningful time period |  | not available |
|  | d) | Consider plots to visualize results (e.g. forest plot, scatterplot of associations between genetic variants and outcome versus between genetic variants and exposure) |  | not available |
| 12 | **Assessment of assumptions** |  |  |  |
|  | a) | Report the assessment of the validity of the assumptions | 14-15 | To investigate potential causal links, a range of variable factors were systematically evaluated in relation to MSMI. For maternal sepsis, multiple factors showed statistically significant associations (P < 0.05), as detailed in Table 2. These included lipid profiles, inflammatory markers, medication use, and pregnancy history variables. Using the inverse variance weighted (IVW) method, we identified several factors with odds ratios (OR) > 1, such as LDL cholesterol, previous smoking, RANTES levels, IL-13 levels, IL-10 levels, birth weight, cholesterol-lowering medications, blood pressure medication, and PSG11, indicating their potential roles as risk factors for the development of maternal sepsis. Conversely, those factor with OR < 1, including HDL cholesterol, TG, TC, IL-1Ra levels, IFN-γ levels, PSG9, and number of pregnancy terminations, emerged as potential protective influences against the development of maternal sepsis.  Analysis of other maternal infections revealed distinct causal profiles, as detailed in Table 3. Statistically significant causal relationships (P < 0.05) were identified between multiple factors and other maternal infections, including spontaneous miscarriage or termination, BMI, CRP, LDL cholesterol, TC, TG, IL-13 levels, IL-2Ra levels, NT-proBNP, cholesterol-lowering medications, blood pressure medication, and vitamin D levels. These findings indicated their potential causal roles in the development of other maternal infections. Using the IVW method, factors with OR > 1 were classified as potential risk factors of other maternal infections, such as spontaneous miscarriage or termination, CRP, IL-13 levels, NT-proBNP, blood pressure medication, and vitamin D levels. Conversely, the IVW method found that BMI, LDL cholesterol, total cholesterol, triglycerides, IL-2Ra levels, and cholesterol-lowering medication (all with OR < 1) emerged as potential protective factors, suggesting a negative association with infection risk. |
|  | b) | Report any additional statistics (e.g., assessments of heterogeneity across genetic variants, such as *I^2^*, Q statistic or E-value) | 15 | All instrumental variables showed F-statistics greater than 10 (S7 Table), indicating a low risk of weak instrument bias. The results of harmonization analyses in the Mendelian randomization analysis were available in S8 Table. The results of causal effects using five Mendelian randomization methods were summarized in S9 Table. The validity of MR estimates was systematically evaluated through pleiotropy (S10 Table), heterogeneity (S11 Table), and directionality tests (S12 Table). As summarized in Table 4, all Cochran’s Q statistics returned P-values greater than 0.05, indicating negligible heterogeneity among instrumental variables and supporting the use of fixed-effects IVW models. Similarly, pleiotropy assessment via MR-Egger and MR-PRESSO showed no evidence of horizontal pleiotropy (all P > 0.05, Table 5), confirming the robustness of causal estimates. The detailed results of MR-PRESSO global test were available in S13 Table. Finally, directionality testing verified the assumed causal direction, with outcome variance consistently lower than exposure variance across all analyses (Table 6) . |
| 13 | **Sensitivity analyses and additional analyses** |  |  |  |
|  | a) | Report any sensitivity analyses to assess the robustness of the main results to violations of the assumptions | 15 | The validity of MR estimates was systematically evaluated through pleiotropy (S10 Table), heterogeneity (S11 Table), and directionality tests (S12 Table). As summarized in Table 4, all Cochran’s Q statistics returned P-values greater than 0.05, indicating negligible heterogeneity among instrumental variables and supporting the use of fixed-effects IVW models. Similarly, pleiotropy assessment via MR-Egger and MR-PRESSO showed no evidence of horizontal pleiotropy (all P > 0.05, Table 5), confirming the robustness of causal estimates. The detailed results of MR-PRESSO global test were available in S13 Table. Finally, directionality testing verified the assumed causal direction, with outcome variance consistently lower than exposure variance across all analyses (Table 6) . |
|  | b) | Report results from other sensitivity analyses or additional analyses | 15-16 | The results of the sensitivity analysis showed that no significantly deviating SNPs were found in this study (S5 Fig for maternal sepsis and S6 Fig for other maternal infections), indicating that the analysis results are stable and reliable. |
|  | c) | Report any assessment of direction of causal relationship (e.g., bidirectional MR) | 15 | The validity of MR estimates was systematically evaluated through pleiotropy (S10 Table), heterogeneity (S11 Table), and directionality tests (S12 Table). As summarized in Table 4, all Cochran’s Q statistics returned P-values greater than 0.05, indicating negligible heterogeneity among instrumental variables and supporting the use of fixed-effects IVW models. Similarly, pleiotropy assessment via MR-Egger and MR-PRESSO showed no evidence of horizontal pleiotropy (all P > 0.05, Table 5), confirming the robustness of causal estimates. The detailed results of MR-PRESSO global test were available in S13 Table. Finally, directionality testing verified the assumed causal direction, with outcome variance consistently lower than exposure variance across all analyses (Table 6) . |
|  | d) | When relevant, report and compare with estimates from non-MR analyses |  | not available |
|  | e) | Consider additional plots to visualize results (e.g., leave-one-out analyses) |  | not available |
|  | **DISCUSSION** |  |  |  |
| 14 | **Key results** | Summarize key results with reference to study objectives | 15-16 | Our Mendelian randomization analyses provide novel causal evidence linking specific biomarkers to MSMI risk. The identification of inflammatory markers (RANTES, IL-10, CRP) as risk factors reinforces the central role of dysregulated immunity in sepsis pathogenesis [36]. Meanwhile, the protective associations of HDL cholesterol and IL-1Ra suggest potential compensatory mechanisms that could be therapeutically targeted, aligning with emerging research on metabolic-inflammation crosstalk in infection susceptibility [37]. |
| 15 | **Limitations** | Discuss limitations of the study, taking into account the validity of the IV assumptions, other sources of potential bias, and imprecision. Discuss both direction and magnitude of any potential bias and any efforts to address them | 19-21 | Several limitations warrant consideration when interpreting our results. First, heterogeneity in diagnostic capacity and reporting systems across countries may affect the accuracy of MSMI burden estimates, particularly in low-SDI regions. In low-resource settings, limited laboratory capacity and incomplete surveillance systems may lead to underreporting and misclassification, especially for other maternal infections. Such systematic bias may result in underestimation of the true disease burden and may weaken the observed inverse relationship between SDI and MSMI burden. Despite robust quality control, heterogeneity in diagnostic capability and reporting completeness across countries may affect burden estimates, particularly in low-resource settings [56]. This concern is especially relevant given our finding that diagnostic quality varies with socioeconomic development (Fig. 9). Second, the our MR analyses assume linearity of causal effects and may not capture threshold or non-linear relationships. Although MR reduces confounding, it relies on key assumptions, including the absence of unmeasured confounding of the instrument–outcome association. For complex exposures such as inflammatory cytokines and behavioral traits, genetic instruments may influence outcomes through multiple biological pathways, introducing potential bias [57]. Therefore, despite the application of sensitivity analyses to detect pleiotropy, residual bias from unknown or unmeasured pathways cannot be completely excluded. This limitation has been widely recognized in MR studies involving complex traits. Additionally, the majority of genetic instruments were derived from European-ancestry populations, which may limit the generalizability of the findings to other populations with different genetic architectures[58]. Differences in environmental exposures and gene–environment interactions across populations may further influence the validity of causal estimates. This limitation is particularly relevant for high-burden regions such as Africa and South Asia, where the applicability of the findings may be reduced. The statistical power of the MR analyses may have been insufficient to detect small but clinically meaningful effects, particularly for exposures with a limited number of genome-wide significant SNPs. Although a strict F-statistic threshold (>10) was applied to ensure strong instruments and minimize weak instrument bias, it is important to note that MR studies are inherently constrained by the variance in the exposure explained by the genetic instruments. With a limited number of SNPs, the statistical power to detect modest causal effects is reduced. Thus, null findings should not be interpreted as definitive evidence of no causal relationship, but rather as an indication that any true effect may be smaller than the study was designed to detect. Future investigations leveraging expanded GWAS datasets or employing multi-variable MR approaches to aggregate genetic signals may help overcome these power limitations. It is acknowledged that overlap between exposure and outcome GWAS samples may introduce bias in two-sample MR analyses, particularly by inflating Type I error rates and underestimating standard errors. In this study, this risk was mitigated by selecting exposure and outcome datasets from independent, large-scale consortium efforts. Specifically, the use of UK Biobank-based GWAS for exposures and data from distinct sources (e.g., the FinnGen consortium for outcomes) represents a strategy to minimize overlap, given their differing study populations, ascertainment strategies, and geographic distributions. Therefore, substantial sample overlap is unlikely in this study. Nevertheless, the possibility of partial overlap due to shared control populations or unreported study affiliations cannot be entirely excluded. Future studies utilizing strictly non-overlapping datasets or applying methods to correct for sample overlap (e.g., MRlap, pseudo-IVW) will be valuable to further validate our findings. Third, the forecasting models, though statistically validated, rely on historical trends and may not account for future healthcare breakthroughs, emerging antimicrobial resistance, or climate change impacts on infection patterns [59]. |
| 16 | **Interpretation** |  |  |  |
|  | a) | Meaning: Give a cautious overall interpretation of results in the context of their limitations and in comparison with other studies | 17-18 | The MR analyses provide novel causal evidence linking specific biomarkers to MSMI risk. The identification of inflammatory markers (RANTES, IL-10, CRP) as risk factors reinforces the central role of dysregulated immunity in sepsis pathogenesis [39]. Importantly, the differential associations observed in the MR analysis may reflect distinct immunopathological mechanisms underlying MSMI. RANTES and IL-10, identified as independent risk factors for maternal sepsis, are closely involved in immune regulation and anti-inflammatory processes. Elevated IL-10 levels, in particular, may indicate an excessive compensatory anti-inflammatory response, leading to immune suppression and impaired pathogen clearance during sepsis progression [40]. Similarly, RANTES plays a key role in leukocyte recruitment and immune cell activation, and its dysregulation may contribute to maladaptive immune responses in severe infections [41]. In contrast, CRP and IL-13, which were associated with other maternal infections, are more strongly linked to systemic inflammatory responses and host defense mechanisms. CRP is a well-established marker of acute inflammation, reflecting the intensity of the innate immune response [42], while IL-13 is involved in Th2-mediated immune pathways and may influence susceptibility to certain infectious processes [43]. Although not retained as primary factors in the MVMR analysis, some protective biomarkers such as HDL and IL-1Ra may still possess potential translational relevance. HDL has been widely recognized for its anti-inflammatory and immunomodulatory properties [44], while IL-1Ra functions as a natural inhibitor of IL-1–mediated inflammatory signaling [45]. These findings suggest that modulation of inflammatory pathways may represent a potential avenue for prevention or intervention. Nevertheless, further studies are required to validate these effects in clinical settings. |
|  | b) | Mechanism: Discuss underlying biological mechanisms that could drive a potential causal relationship between the investigated exposure and the outcome, and whether the gene-environment equivalence assumption is reasonable. Use causal language carefully, clarifying that IV estimates may provide causal effects only under certain assumptions | 17-18 | The MR analyses provide novel causal evidence linking specific biomarkers to MSMI risk. The identification of inflammatory markers (RANTES, IL-10, CRP) as risk factors reinforces the central role of dysregulated immunity in sepsis pathogenesis [39]. Importantly, the differential associations observed in the MR analysis may reflect distinct immunopathological mechanisms underlying MSMI. RANTES and IL-10, identified as independent risk factors for maternal sepsis, are closely involved in immune regulation and anti-inflammatory processes. Elevated IL-10 levels, in particular, may indicate an excessive compensatory anti-inflammatory response, leading to immune suppression and impaired pathogen clearance during sepsis progression [40]. Similarly, RANTES plays a key role in leukocyte recruitment and immune cell activation, and its dysregulation may contribute to maladaptive immune responses in severe infections [41]. In contrast, CRP and IL-13, which were associated with other maternal infections, are more strongly linked to systemic inflammatory responses and host defense mechanisms. CRP is a well-established marker of acute inflammation, reflecting the intensity of the innate immune response [42], while IL-13 is involved in Th2-mediated immune pathways and may influence susceptibility to certain infectious processes [43]. Although not retained as primary factors in the MVMR analysis, some protective biomarkers such as HDL and IL-1Ra may still possess potential translational relevance. HDL has been widely recognized for its anti-inflammatory and immunomodulatory properties [44], while IL-1Ra functions as a natural inhibitor of IL-1–mediated inflammatory signaling [45]. These findings suggest that modulation of inflammatory pathways may represent a potential avenue for prevention or intervention. Nevertheless, further studies are required to validate these effects in clinical settings. |
|  | c) | Clinical relevance: Discuss whether the results have clinical or public policy relevance, and to what extent they inform effect sizes of possible interventions | 19 | Finally, expanding genomic studies to include underrepresented populations will improve the generalizability of causal inferences and help advance precision public health approaches to maternal infection prevention. Longitudinal studies tracking how changing SDI modifies MSMI burden could also provide valuable insights for targeting interventions most effectively. |
| 17 | **Generalizability** | Discuss the generalizability of the study results (a) to other populations, (b) across other exposure periods/timings, and (c) across other levels of exposure | 20 | Additionally, the majority of genetic instruments were derived from European-ancestry populations, which may limit the generalizability of the findings to other populations with different genetic architectures[58]. Differences in environmental exposures and gene–environment interactions across populations may further influence the validity of causal estimates. This limitation is particularly relevant for high-burden regions such as Africa and South Asia, where the applicability of the findings may be reduced. The statistical power of the MR analyses may have been insufficient to detect small but clinically meaningful effects, particularly for exposures with a limited number of genome-wide significant SNPs. Although a strict F-statistic threshold (>10) was applied to ensure strong instruments and minimize weak instrument bias, it is important to note that MR studies are inherently constrained by the variance in the exposure explained by the genetic instruments. With a limited number of SNPs, the statistical power to detect modest causal effects is reduced. Thus, null findings should not be interpreted as definitive evidence of no causal relationship, but rather as an indication that any true effect may be smaller than the study was designed to detect. |
|  | **OTHER INFORMATION** |  |  |  |
| 18 | **Funding** | Describe sources of funding and the role of funders in the present study and, if applicable, sources of funding for the databases and original study or studies on which the present study is based | 22 | This work was supported by no funding. |
| 19 | **Data and data sharing** | Provide the data used to perform all analyses or report where and how the data can be accessed, and reference these sources in the article. Provide the statistical code needed to reproduce the results in the article, or report whether the code is publicly accessible and if so, where | 4-22 | Fig1  Table1 |
| 20 | **Conflicts of Interest** | All authors should declare all potential conflicts of interest | 22 | The authors declare no conflict of interest. |

This checklist is copyrighted by the Equator Network under the Creative Commons Attribution 3.0 Unported (CC BY 3.0) license.

1. Skrivankova VW, Richmond RC, Woolf BAR, Yarmolinsky J, Davies NM, Swanson SA, et al. Strengthening the Reporting of Observational Studies in Epidemiology using Mendelian Randomization (STROBE-MR) Statement. JAMA. 2021;under review.

2. Skrivankova VW, Richmond RC, Woolf BAR, Davies NM, Swanson SA, VanderWeele TJ, et al. Strengthening the Reporting of Observational Studies in Epidemiology using Mendelian Randomisation (STROBE-MR): Explanation and Elaboration. BMJ. 2021;375:n2233.
