# Supplementary material for: Global burden, projections, and causal factors of maternal sepsis and other maternal infections: A comprehensive epidemiological and mendelian randomization study
Source: PLoS Negl Trop Dis. 2026 May 27;20(5):e0014374. doi: 10.1371/journal.pntd.0014374 (PMC13229374; doi:10.1371/journal.pntd.0014374)
Supplement: Attachment — Submitted filename: Response to Reviewers.docx [file pntd.0014374.s022.docx]

**Dear Editor and Reviewers,**

We sincerely thank you for your valuable comments and constructive suggestions regarding our manuscript entitled *“Global Burden, Projections, and Causal Factors of Maternal Sepsis and Other Maternal Infections: A Comprehensive Epidemiological and Mendelian Randomization Study”* (Manuscript ID: PNTD-D-25-02202). These insightful comments have significantly contributed to improving the quality of our manuscript.

We have carefully revised the manuscript in accordance with all reviewers’ comments and provided point-by-point responses to each issue raised. All modifications in the revised manuscript have been clearly highlighted in color to facilitate review. We hope that the revised version meets the publication standards of the journal.

We respectfully submit this revised manuscript for your consideration. Below, we provide detailed responses to the reviewers’ comments.

**Response to Reviewer 1**

We sincerely thank the reviewer for the thoughtful and constructive comments. We have carefully considered each suggestion and have made corresponding revisions to address all raised concerns.

**Response 1:**

We thank the reviewer. We have added model diagnostic information. AIC and BIC were used for ARIMA model selection, and residual tests confirmed model adequacy. For the BAPC model, posterior predictive checks were added to ensure robustness. We have supplemented descriptions of BIC and INLA in the Materials and Methods section (line 255-256,260-261), and further explored the reliability of the two models in the Discussion section (line 577-584).

**Response 2:**

We thank the reviewer for this valuable suggestion. We have now added Spearman correlation analysis to formally assess the relationship between SDI and MSMI burden (line 206-218). Since the SDI is a composite index, we further performed a decomposition analysis to investigate the relative contributions of its individual components (line 218-231), which could provide more precise insights into social determinants. In addition, we included a brief discussion on the components of SDI, indicating that education level and fertility may play more important roles. These revisions have been added to Materials and Methods (line 206-231), the Results (line 337-340, 343-376) and Discussion sections(line 504-509).

**Response 3:**

We thank the reviewer for this valuable suggestion. We have added a quantitative estimate of the contribution of population growth to the increase in MSMI cases in low-SDI regions. The results show that population growth accounts for the majority of the increase in absolute case numbers. These results have been added to the Results (line 398-410) and Discussion sections(line 519-522).

**Response 4**

We thank the reviewer for this valuable suggestion. We have added country-specific examples in the Discussion to better explain the observed heterogeneity across countries. These examples provide real-world context and improve the interpretability of our global findings (line 496-501).

**Response 5**

We thank the reviewer for this important comment. We have expanded the Discussion to better address potential misclassification and underreporting in low-resource settings. We further clarified that such bias may lead to underestimation of MSMI burden and may attenuate the observed association between SDI and disease burden (line 600-607).

**Response 6**

We thank the reviewer for this important comment. We have now added the formula for F-statistic calculation in the Methods section (line 202-204). In addition, we reported the F-statistics for all instrumental variables, which were all above the conventional threshold of 10 in the Results section (line 461-462), indicating low risk of weak instrument bias. A summary of F-statistics for all exposures was summarized in S7 Table.

**Response 7**

We thank the reviewer for this important comment. We have clarified the LASSO procedure in the Methods section, including the selection of the tuning parameter λ using 10-fold cross-validation (line 298-301). We have also explicitly reported the variables retained after LASSO selection in the Results section (line 478-480). These revisions improve the transparency and reproducibility of the variable selection process.

**Response 8**

We thank the reviewer for this insightful comment. We have expanded the Discussion to acknowledge that Mendelian randomization relies on key assumptions, including the absence of unmeasured genetic confounding (line 612-619). We further noted that for complex exposures such as inflammatory cytokines and behavioral traits, residual bias due to unknown or pleiotropic pathways may still exist, despite the use of sensitivity analyses (line 612-619).

**Response 9**

We thank the reviewer for this important comment. We have expanded the Discussion to further address the limitation related to the predominance of European-ancestry data in our MR analysis (line 619-625). We clarified that differences in genetic architecture, environmental exposures, and gene–environment interactions across populations may limit the generalizability of our findings, particularly in high-burden regions such as Africa and South Asia (line 619-625).

**Response 10**

We thank the reviewer for this insightful comment. We acknowledge that statistical power is an important consideration in Mendelian randomization analyses, particularly for exposures with a limited number of SNPs. Due to data constraints, we did not perform formal power calculations. However, we restricted our analyses to instrumental variables with F-statistics greater than 10 to reduce weak instrument bias. We have now clarified in the Discussion that limited statistical power may reduce the ability to detect small but clinically meaningful effects, and that null findings should be interpreted with caution (line 625-636).

**Response 11**

We thank the reviewer for this important comment. We acknowledge that sample overlap between exposure and outcome GWAS may introduce bias in Mendelian randomization analyses. In our study, we carefully selected exposure and outcome datasets from independent large-scale genome-wide association studies to minimize potential overlap (line 158-184). Therefore, the risk of bias due to sample overlap is expected to be limited. We have also added this as a limitation in the Discussion section (line 636-649).

**Response 12**

We sincerely thank the reviewer for this important comment. We acknowledge that the number of instrumental variables for several exposures was limited, which may reduce statistical power and increase uncertainty in causal estimates. However, to ensure the validity of the Mendelian randomization assumptions, we selected SNPs using a stringent genome-wide significance threshold (P < 5 × 10⁻⁸) and strict linkage disequilibrium criteria (line 191-193). Relaxing these criteria may introduce weak or invalid instruments, thereby increasing the risk of bias and violating the core assumptions of MR analysis. Therefore, we chose to prioritize instrument validity over quantity. In addition, all included instruments demonstrated adequate strength (F-statistics > 10), suggesting a low likelihood of weak instrument bias(line 461-462).

**Response 13**

We sincerely thank the reviewer for this insightful comment. We agree that stronger integration between the epidemiological findings and the Mendelian randomization (MR) results would improve the overall coherence of the manuscript. In response, we have revised the Discussion section to explicitly link the observed global burden patterns with the identified causal risk factors. Specifically, we incorporated a new paragraph (lines 549-565) highlighting that regions with a high MSMI burden, particularly low- and middle-SDI countries in South Asia and sub-Saharan Africa, are often characterized by increased infection exposure, poor nutritional status, and limited healthcare access, which may promote chronic low-grade inflammation. We further discussed that previous epidemiological evidence has reported elevated levels of inflammatory markers in populations under such conditions, which is consistent with our MR findings identifying CRP, IL-13, and RANTES as causal risk factors.

This added discussion provides a biologically plausible link between adverse socioeconomic environments, systemic inflammation, and increased MSMI risk, thereby improving the integration between the descriptive epidemiological analysis and causal inference. We believe this revision substantially enhances the interpretability and coherence of the manuscript.

**Response 14**

Thank you for this insightful comment. We agree that the biological interpretation of the Mendelian randomization findings required further clarification. In the revised manuscript, we have expanded the Discussion to provide a mechanistic explanation for the differential roles of inflammatory biomarkers in MSMI subtypes. Specifically, we discuss how RANTES and IL-10, which are involved in immune regulation and anti-inflammatory responses, may reflect dysregulated immune suppression during sepsis progression, whereas CRP and IL-13 are more closely linked to systemic inflammation and host defense responses in non-sepsis maternal infections. These findings suggest that distinct immunopathological mechanisms may underlie maternal sepsis and other maternal infections. The corresponding text has been added to the Discussion section (line 528-541).

**Response 15**

Thank you for this insightful suggestion. We agree that the protective factors identified in our analysis may have potential translational implications. In the revised manuscript, we have prioritized the discussion of key biomarkers identified in the multivariable Mendelian randomization analysis, including RANTES, IL-10, IL-13 and CRP, to maintain a clear and focused interpretation of the main findings (line 528-541). At the same time, we have added a concise discussion on the potential translational relevance of protective factors such as HDL and IL-1Ra. These factors were not the primary focus of the multivariable analysis but may still provide useful insights into potential preventive or therapeutic strategies (line 541-548). The corresponding text has been added to the Discussion section (line 528-548).

**Response 16**

Thank you for this insightful suggestion. We agree that the original abstract and conclusion were primarily descriptive and did not sufficiently emphasize the integrative analytical framework and its scientific contribution. In response, we have rewritten both the Abstract section(line 30-49) and Conclusions section (line 653-664) to clearly highlight that this study is, to our knowledge, the first to integrate global burden of disease trend analysis with multivariable Mendelian randomization. This combined approach provides complementary epidemiological and causal inference evidence, offering a more robust basis for identifying potential risk factors and informing precision public health strategies. These revisions strengthen the novelty and translational significance of our study.

**Response 17**

Thank you for this constructive suggestion. We agree that the presentation of global maps with numerous countries may reduce visual clarity and obscure key findings. In response, we have improved the visualization strategy by incorporating regional-level summaries to complement country-level maps and by highlighting countries with significant increasing trends in Figure 10. In addition, all figure titles and legends have been revised to ensure they are self-contained and interpretable without reference to the main text. These revisions enhance the clarity and interpretability of the visual results while preserving the completeness of the global analysis.

**Response 18**

Thank you for this valuable comment. We agree that consistency in the use of abbreviations is essential for clarity. Accordingly, we have revised the manuscript to ensure that the full term “maternal sepsis and other maternal infections” is defined at its first occurrence, and the abbreviation “MSMI” is subsequently used consistently throughout the text. In addition, we carefully reviewed the entire manuscript and identified other instances of inconsistent or non-standard abbreviation usage, all of which have been corrected to improve clarity and consistency.

**Response 19**

Thank you for this valuable comment. In response, we have added the results of harmonization and other sensitivity analyses in the Mendelian randomization analysis to the supplementary materials (Table S8-S13). Specifically, we performed a leave-one-out sensitivity analysis and have incorporated the corresponding descriptions into the Methods (line 284-289), Results (line 474-477), and the supplementary materials.

**Response 20**

Thank you for this valuable suggestion. We agree that clear definitions of all column variables are essential for improving readability and interpretability of the Supplementary Tables.

In response, we have added detailed descriptions for all column indicators in each Supplementary Table, including definitions of effect estimates, confidence intervals, P values, and instrumental variable metrics where applicable. These clarifications have been incorporated directly into the Supplementary Materials to ensure transparency and improve usability for readers.

**Response 21**

Thank you for this constructive suggestion. We fully agree that clarity, conciseness, and linguistic accuracy are essential for improving the overall quality of the manuscript.

In response, the entire manuscript has been carefully revised and professionally edited to correct grammatical errors, improve sentence structure, and enhance clarity and readability. We have also reduced sentence redundancy and simplified overly complex expressions to improve the overall flow and precision of the text.

**Reviewer 2:**

We sincerely thank Reviewer 2 for the time and effort in evaluating our manuscript. No additional comments were provided for revision.

We would like to thank the Editor and all reviewers once again for their time, effort, and constructive comments, which have significantly improved the quality of our manuscript. We hope that the revised version is now suitable for publication in *PLOS Neglected Tropical Diseases*.
